# Supplementary material for: Enantiopure synthesis of [5]helicene based molecular lemniscates and their use in chiroptical materials
Source: Nat Commun. 2025 Mar 22;16:2837. doi: 10.1038/s41467-025-58162-1 (PMC11929746; doi:10.1038/s41467-025-58162-1)
Supplement: Supplementary file 1 — Supplementary Information [file 41467_2025_58162_MOESM1_ESM.pdf]

Supplementary Information

Enantiopure Synthesis of [5]Helicene Based Molecular Lemniscates and Their Use in Chiroptical Materials

Leah E. M. White,[1] Tiberiu-M. Gianga,[2] Fabienne Pradaux-Caggiano,[1] Chiara Faverio,[1] Andrea Taddeucci,[2,3] Henry S. Rzepa,[4] Christian Jonhannesen,[5] Lauren E. Hatcher,[6] Giuliano Siligardi,[2] David R. Carbery,[1] G. Dan Pantoș[1]*

[1]Department of Chemistry, University of Bath, Bath BA2 7AY, UK
[2]B23 Beamline, Diamond Light Source Ltd., Didcot OX11 0DE, UK
[3]Department of Chemistry and Industrial Chemistry, University of Pisa, Via Giuseppe Moruzzi 13, Pisa 56124, Italy
[4]Department of Chemistry, Imperial College London MSRH, White City Campus, 80 Wood Lane, London W12 0BZ, UK,
[5]Department of Chemistry, University of Antwerp, 2020 Antwerp, Belgium
[6]School of Chemistry, Cardiff University, Park Place, Cardiff, CF10 3AT, UK

*Correspondence: g.d.pantos@bath.ac.uk

Table of Contents

1. *Supplementary Synthetic Procedures*............3
2. *Supplementary NMR Spectra*............14
3. *Supplementary Single-Crystal X-Ray Diffraction Data*............29
4. *Supplementary Computational Methods*............31
5. *Supplementary Data: Conformational Stability of Helicenoids 6 and 7*............32
6. *Supplementary Data: Self-Sorting Studies*............35
7. *Supplementary MMP spectra and maps*............36
8. *Supplementary CPL spectra*............48
9. *Supplementary CD and Variable Temperature CD spectra*............51
10. *Supplementary Emission spectra*............53
11. *References*............55

1. Supplementary Synthetic Procedures

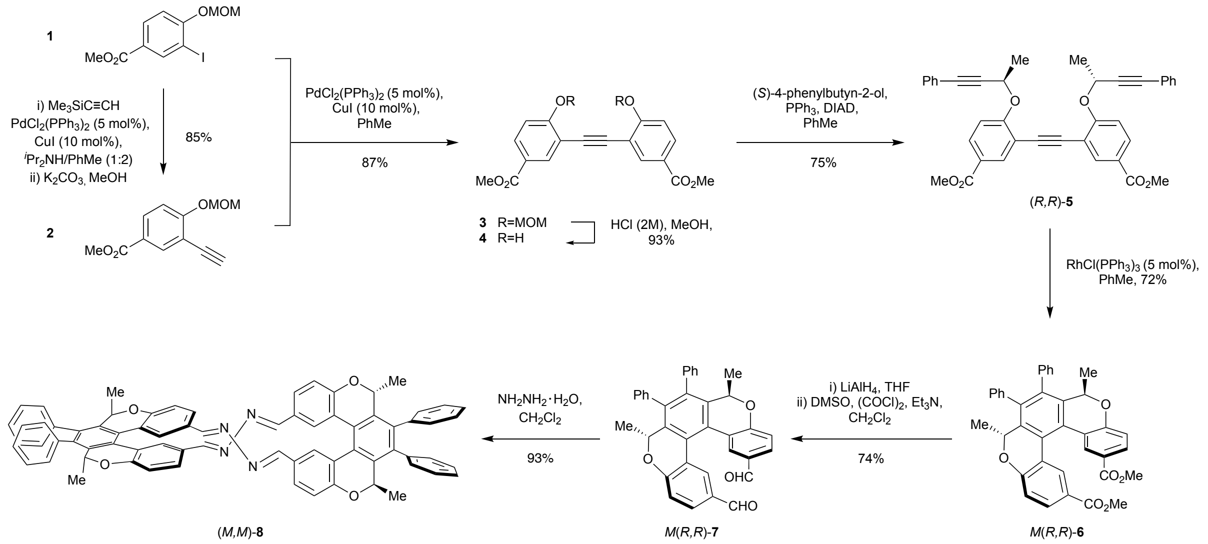

Supplementary Figure 1. Synthetic route to lemniscate (*M,M*)-8. Enantiomeric lemniscate (*P,P*)-8 was synthesised in an identical synthetic sequence using (*R*)-4-phenylbutyn-2-ol in step 4 (see Figure 1).

Synthesis of 1

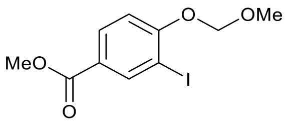

Under an atmosphere of N_2, anhydrous CH_2Cl_2 (30 mL) was added to methyl 4-hydroxy-3-iodobenzoate (3.00 g, 10.8 mmol, 1.0 equiv). The resulting suspension was cooled to 0 °C and diisopropylamine (2.0 mL) was added to form a clear solution. To this, chloromethyl methyl ether (0.90 mL, 11.9 mmol, 1.1 equiv) was added dropwise at 0 °C. The reaction mixture was stirred for 1 h and then quenched by addition of a saturated ammonium chloride solution (20 mL). The product was extracted with dichloromethane (3 x 20 mL) and the combined organic extracts were dried over $MgSO_4$ and filtered. Solvents were removed *in vacuo* yielding the desired product **1** as a white solid (3.35 g, 10.4 mmol, 96%). No purification was required. Mp: 92-94 °C.

^{1}H NMR (400 MHz, $CDCl_3$) δ_H 8.46 (d, J = 2.1 Hz, 1H, Ar-CH), 7.97 (dd, J = 8.7, 2.1 Hz, 1H, Ar-CH), 7.07 (d, J = 8.7 Hz, 1H, Ar-CH), 5.29 (s, 2H, CH_2), 3.88 (s, 3H, CH_3), 3.50 (s, 3H, CH_3). ^{13}C NMR (75 MHz, $CDCl_3$) δ_C 165.6, 159.7, 141.2, 131.5, 125.4, 113.5, 94.8, 86.4, 56.8, 52.3. ν_{max} (cm^{-1}): 2959, 2927, 2828, 1709, 1698. HR-MS (ESI) *m/z* calculated for $C_{10}H_{11}IO_4Na$ $[M+Na]^+$ 344.9599, found 344.9594.

Synthesis of S1

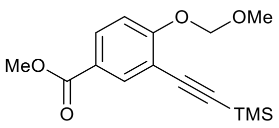

Compound **1** (1.88 g, 5.83 mmol, 1.0 equiv), bis(triphenylphosphine)palladium(II) dichloride (204 mg, 0.29 mmol, 5 mol%) and copper(I) iodide (111 mg, 0.58 mmol, 10 mol%) were combined under an N_2 atmosphere and dissolved in an anhydrous toluene/diisopropylamine mixture (24 mL/ 12 mL) to form a dark orange solution. Trimethylsilylacetylene (1.07 mL, 7.6 mmol, 1.2 eq.) was added dropwise at RT and the resulting dark brown solution was stirred for 2 h. Solvents were removed *in vacuo* prior to purification by flash chromatography (petroleum ether:EtOAc, (10:0 – 8:2 *v/v* ratio)), yielding the desired product **S1** as a white solid (1.63 g, 5.57 mmol, 96%). Mp: 59-60 °C.

^{1}H NMR (400 MHz, $CDCl_3$) δ 8.13 (d, *J* = 2.2 Hz, 1H, Ar-CH), 7.93 (dd, *J* = 8.8, 2.2 Hz, 1H, Ar-CH), 7.11 (d, *J* = 8.8 Hz, 1H, Ar-CH), 5.29 (s, 2H, CH_2), 3.89 (s, 3H, CH_3), 3.53 (s, 3H, CH_3), 0.26 (s, 9H, 3 x CH_3). ^{13}C NMR (75 MHz, $CDCl_3$) δ_C 166.3, 161.6, 135.9, 131.6, 123.7, 114.3, 113.8, 100.1, 99.5, 94.9, 56.6, 52.2, 0.1. ν_{max} (cm^{-1}): 2955, 2159, 1720, 1602, 1262. HR-MS (ESI) *m/z* calculated for $C_{15}H_{21}O_4Si$ $[M+H]^+$ 293.1209, found 293.1211.

Synthesis of 2

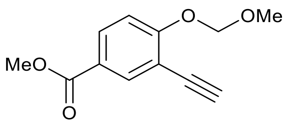

Methanol (58 mL) was added to compound **S1** (1.63 g, 5.57 mmol, 1.0 equiv) and the resulting suspension was cooled to 0 °C. To this, potassium carbonate (1.93 g, 13.9 mmol, 2.5 equiv) was added and the reaction mixture was stirred for 20 min at 0 °C. The reaction mixture was then quenched with water (30 mL) and the aqueous phase was extracted with EtOAc (3 x 20 mL). The combined organic extracts were washed with a saturated sodium chloride solution, dried over $MgSO_4$ and filtered. Solvents were removed *in vacuo* prior to purification by flash chromatography (petroleum ether:EtOAc, (10:0 – 8:2 *v/v* ratio)), yielding the desired product **2** as a brown solid (1.12 g, 5.09 mmol, 92%). Mp: 79-81 °C.

^{1}H NMR (400 MHz, $CDCl_3$) δ 8.16 (d, *J* = 2.2 Hz, 1H, Ar-CH), 7.98 (dd, *J* = 8.8, 2.2 Hz, 1H, Ar-CH), 7.17 (d, *J* = 8.8 Hz, 1H, Ar-CH), 5.32 (s, 2H, CH_2), 3.89 (s, 3H, CH_3), 3.52 (s, 3H, CH_3), 3.30 (s, 1H, CH). ^{13}C NMR (75 MHz, $CDCl_3$) δ_C 166.1, 161.8, 136.0, 132.0, 123.7, 114.0, 112.4, 94.7,

81.9, 79.1, 56.6, 52.3. ν_{max} (cm^{-1}): 3238, 2962, 2928, 2829, 1714, 1702. HR-MS (ESI) *m/z* calculated for $C_{12}H_{12}O_4Na$ $[M+Na]^+$ 243.0633, found 243.0628.

Synthesis of 3

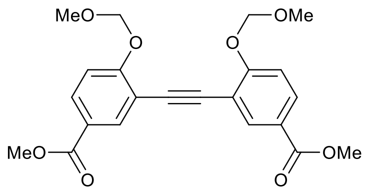

Compound **1** (3.07 g, 9.53 mmol, 1.0 equiv), tetrakis(triphenylphosphine)palladium(0) (551 mg, 0.48 mmol, 5 mol%), and copper(I) iodide (181 mg, 0.95 mmol, 10 mol%) were combined under an N_2 atmosphere and dissolved in anhydrous toluene (120 mL). To this, a solution of compound **2** (2.10 g, 9.53 mmol, 1.0 equiv) in toluene (30 mL) was added. Diisopropylamine (56 mL) was subsequently added slowly over 5 min and the resulting mixture was stirred for 1 h at RT. Solvents were removed *in vacuo* prior to purification by flash chromatography (petroleum ether:EtOAc, (6:4 – 4:6 *v/v* ratio)), yielding the desired product **3** as a light yellow solid (3.43 g, 8.28 mmol, 87%). Mp: 117-119 °C.

^1H NMR (400 MHz, $CDCl_3$) δ_H 8.22 (d, *J* = 2.2 Hz, 2H, 2 x Ar-CH), 7.97 (dd, *J* = 8.8, 2.2 Hz, 2H, 2 x Ar-CH), 7.18 (d, *J* = 8.8 Hz, 2H, 2 x Ar-CH), 5.35 (s, 4H, 2 x CH_2), 3.91 (s, 6H, 2 x CH_3), 3.55 (s, 6H, 2 x CH_3). ^{13}C NMR (75 MHz, $CDCl_3$) δ_C 166.2, 161.2, 135.3, 131.5, 123.7, 114.2, 113.8, 94.8, 89.3, 56.6, 52.2. ν_{max} (cm^{-1}): 2955, 2839, 1718, 1237. HR-MS (ESI) *m/z* calculated for $C_{22}H_{22}O_8Na$ $[M+Na]^+$ 437.1212, found 437.1247.

Synthesis of 4

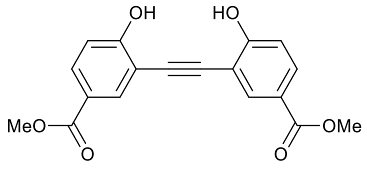

Compound **3** (1.0 g, 2.41 mmol, 1.0 equiv) was suspended in methanol (50 mL). To this, $HCl_{(aq)}$ 2M (5.82 mL) was added and the resulting mixture was immediately heated at 90 °C for 20 min. The reaction mixture was then removed from the heat, cooled to RT and diluted in H_2O (50 mL) to form a white precipitate. The aqueous phase was extracted with CH_2Cl_2 (3 x 50 mL) and the combined organic extracts were dried over $MgSO_4$ and filtered. Solvents were removed *in vacuo*, yielding the desired product **4** as an off-white solid (734 mg, 2.25 mmol, 93%). No purification was required. Mp: 249-250 °C.

^{1}H NMR (400 MHz, acetone-d_6) δ_H 9.48 (br s, 2H, 2 x OH), 8.13 (d, J = 2.2 Hz, 2H, 2 x Ar-CH), 7.91 (dd, J = 8.6, 2.2 Hz, 2H, 2 x Ar-CH), 7.07 (d, J = 8.6 Hz, 2H, 2 x Ar-CH), 3.86 (s, 6H, 2 x CH_3). ^{13}C NMR (101 MHz, acetone-d_6) δ_C 166.4, 162.6, 135.1, 132.5, 123.0, 116.5, 111.3, 90.3, 52.2. ν_{max} (cm^{-1}): 3337, 2948, 2204, 1714, 1687, 1244. HR-MS (ESI) *m/z* calculated for $C_{18}H_{14}O_6Na$ $[M+Na]^+$ 349.0688, found 349.0685.

Synthesis of (*S*)-4-phenylbutyn-2-ol

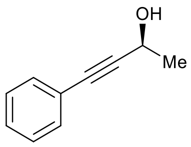

Bis(triphenylphosphine)palladium(II) dichloride (211 mg, 0.30 mmol, 0.7 mol%) and copper(I) iodide (115 mg, 0.60 mmol, 1.4 mol%) were combined under an N_2 atmosphere and dissolved in an anhydrous toluene/ diisopropylamine mixture (78 mL/ 78 mL). Iodobenzene (5.28 mL, 47.1 mmol, 1.1 equiv) was added and the reaction mixture was cooled to 0 °C. To this, (*S*)-but-3-yn-2-ol (3.41 mL, 42.8 mmol, 1.0 equiv) was added dropwise forming a bright orange solution. The reaction mixture was left to warm to RT over 2 h with stirring. Upon completion of the reaction, solvents were removed *in vacuo* prior to purification by flash chromatography (petroleum ether:EtOAc, (10:0 – 8:2 *v/v* ratio)), yielding the desired product (*S*)-4-phenylbutyn-2-ol as a brown oil (6.03 g, 41.2 mmol, 96%).

^{1}H NMR (400 MHz, $CDCl_3$) δ_H 7.45 – 7.40 (m, 2H, 2 x Ar-CH), 7.33 – 7.28 (m, 3H, 3 x Ar-CH), 4.76 (q, J = 6.6 Hz, 1H, CH), 1.56 (d, J = 6.6 Hz, 3H, CH_3). ^{13}C NMR (75 MHz, $CDCl_3$) δ_C 131.8, 128.5, 128.4, 122.7, 91.1, 84.2, 59.0, 24.5. Data in accordance with literature values.[1]

Synthesis of (*R*)-4-phenylbutyn-2-ol

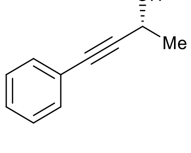

Following the procedure described above with bis(triphenylphosphine)palladium(II) dichloride (147 mg, 0.21 mmol, 0.7 mol%), copper(I) iodide (80 mg, 0.42 mmol, 1.4 mol%), iodobenzene (3.70 mL, 33.0 mmol, 1.1 equiv) and (*R*)-but-3-yn-2-ol (2.39 mL, 30.0 mmol, 1.0 equiv) in toluene/diisopropylamine (56 mL/ 56 mL), the expected product (*R*)-4-phenylbutyn-2-ol was

obtained as a brown oil (4.56 mg, 31.2 mmol, 95%). As expected, the NMR was analogous to that reported above for (*S*)-4-phenylbutyn-2-ol.

^{1}H NMR (400 MHz, $CDCl_3$) δ_H 7.48 – 7.38 (m, 2H, 2 x Ar-CH), 7.33 – 7.28 (m, 3H, 3 x Ar-CH), 4.76 (q, *J* = 6.6 Hz, 1H, CH), 1.56 (d, *J* = 6.6 Hz, 3H, CH_3). ^{13}C NMR (75 MHz, $CDCl_3$) δ_C 131.8, 128.5, 128.4, 122.7, 91.1, 84.2, 59.0, 24.5.

Synthesis of (*S,S*)-5

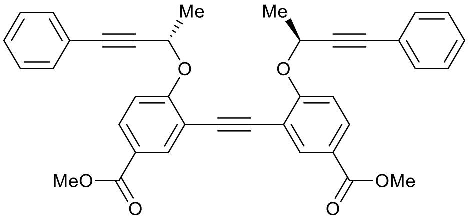

Compound **4** (341 mg, 1.05 mmol, 1.0 equiv) and triphenylphosphine (608 mg, 2.31 mmol, 2.2 equiv) were combined under an N_2 atmosphere and suspended in anhydrous toluene (20 mL). (*R*)-4-phenylbutyn-2-ol (368 mg, 2.52 mmol, 2.4 equiv) was added and the mixture was cooled to 0 °C. DIAD (0.50 mL, 2.52 mmol, 2.4 equiv) diluted in anhydrous toluene (5 mL) was then added dropwise and the resulting mixture was left to warm to RT over 2 h with stirring. Saturated NH_4Cl solution (20 mL) was added and the aqueous phase was extracted with EtOAc (3 x 20 mL). The combined organic extracts were washed with a saturated sodium chloride solution, dried over $MgSO_4$ and filtered. Solvents were removed *in vacuo* prior to purification by flash chromatography (toluene:EtOAc, (10:0 – 9:1 *v/v* ratio)), yielding the desired product (*S*,*S*)-**5** as a light yellow solid (396 mg, 0.68 mmol, 65%). Mp: 83-86 °C.

^{1}H NMR (400 MHz, $CDCl_3$) δ_H 8.24 (d, *J* = 2.2 Hz, 2H, 2 x Ar-CH), 8.01 (dd, *J* = 8.7, 2.2 Hz, 2H, 2 x Ar-CH), 7.40 – 7.35 (m, 4H, 4 x Ar-CH), 7.31 – 7.23 (m, 8H, 8 x Ar-CH), 5.25 (q, *J* = 6.5 Hz, 2H, 2 x CH), 3.90 (s, 6H, 2 x CH_3), 1.88 (d, *J* = 6.5 Hz, 6H, 2 x CH_3). ^{13}C NMR (101 MHz, $CDCl_3$) δ_C 166.4, 161.5, 135.5, 131.9, 131.4, 128.8, 128.4, 123.3, 122.2, 114.0, 113.9, 89.8, 87.4, 86.8, 65.7, 52.2, 22.5. ν_{max} (cm^{-1}): 2992, 2951, 2235, 1898, 1715. HR-MS (ESI) *m/z* calculated for $C_{38}H_{30}O_6Na$ $[M+Na]^+$ 605.1940, found 605.1959.

Synthesis of (*R,R*)-5

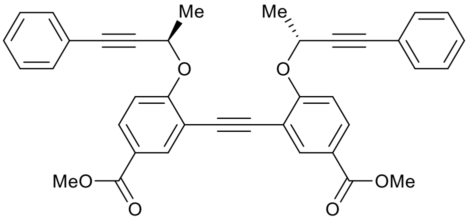

Following the procedure described above with **4** (734 mg, 2.25 mmol, 1.0 equiv), triphenylphosphine (1.30 g, 4.95 mmol, 2.2 equiv), (*S*)-4-phenylbutyn-2-ol (724 mg, 4.95 mmol, 2.2 equiv) and DIAD (1.07 mL, 5.40 mmol, 2.4 equiv) in anhydrous toluene (55 mL), the expected product (*R*,*R*)-**5** was obtained as a light yellow solid (946 mg, 1.62 mmol, 72%). As expected, the NMR was analogous to that reported above for (*S*,*S*)-**5**. Mp: 84-86 °C.

^{1}H NMR (400 MHz, $CDCl_3$) δ_H 8.24 (d, *J* = 2.2 Hz, 2H, 2 x Ar-CH), 8.01 (dd, *J* = 8.7, 2.2 Hz, 2H, 2 x Ar-CH), 7.42 – 7.34 (m, 4H, 4 x Ar-CH), 7.32 – 7.22 (m, 8H, 8 x Ar-CH), 5.25 (q, *J* = 6.5 Hz, 2H, 2 x CH), 3.90 (s, 6H, 2 x CH_3), 1.88 (d, *J* = 6.5 Hz, 6H, 2 x CH_3). ^{13}C NMR (75 MHz, $CDCl_3$) δ_C 166.3, 161.4, 135.4, 131.8, 131.3, 128.7, 128.3, 123.2, 122.1, 113.9, 113.8, 89.7, 87.3, 86.7, 65.6, 52.1, 22.4. ν_{max} (cm^{-1}): 2992, 2949, 2234, 1715, 1231. HR-MS (ESI) *m/z* calculated for $C_{38}H_{31}O_6$ [M+H]$^+$ 583.2121, found 583.2111.

Synthesis of *P*(*S,S*)-6

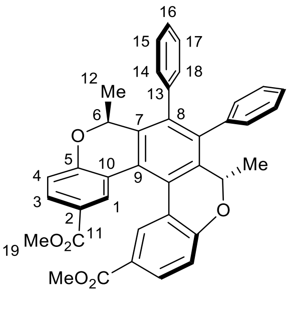

A round-bottomed flask equipped with a condenser was charged with (*S*,*S*)-**5** (394 mg, 0.68 mmol, 1.0 equiv) and tris(triphenylphosphine)rhodium(I) chloride (32 mg, 0.034 mmol, 5 mol%) and placed under an Ar atmosphere. The reagents were dissolved in anhydrous toluene (45 mL) and the resulting mixture was heated at reflux (115 °C) for 24 h. On completion of the reaction, the mixture was cooled and the solvent was removed *in vacuo* prior to purification by flash chromatography (petroleum ether:EtOAc, (10:0 – 8:2 *v/v* ratio)), yielding the desired product *P*(*S*,*S*)-**6** as an off white solid (336 mg, 0.58 mmol, 85%). Mp: 288-290 °C.

^{1}H NMR (400 MHz, $CDCl_3$) δ_H 8.13 (d, J = 2.0 Hz, 2H, H1), 7.90 (dd, J = 8.4, 2.0 Hz, 2H, H3), 7.35 – 7.22 (m, 4H, H17,18), 7.14 (tt, J = 7.4, 1.4 Hz, 2H, H16), 7.10 – 7.03 (m, 4H, H4,15), 6.79 (dt, J = 7.4, 1.4 Hz, 2H, H14), 5.34 (q, J = 6.7 Hz, 2H, H6), 3.70 (s, 6H, H19), 1.00 (d, J = 6.7 Hz, 6H, H12). ^{13}C NMR (101 MHz, $CDCl_3$) δ_C 166.6, 157.8, 138.6, 137.7, 137.5, 130.9, 130.9, 130.8, 129.2, 128.1, 128.0, 127.1, 124.8, 123.1, 122.3, 119.4, 73.5, 51.8, 18.7. ν_{max} (cm^{-1}): 3055, 3023, 2985, 2950, 1709, 1259. HR-MS (ESI) *m/z* calculated for $C_{38}H_{30}O_6Na$ $[M+Na]^+$ 605.1940, found 605.1942.

Synthesis of *M*(*R,R*)-6

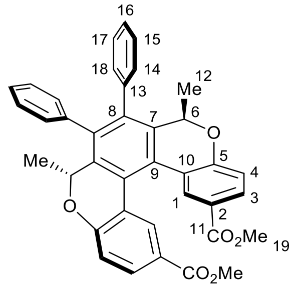

Following the procedure described above with (*R*,*R*)-**5** (900 mg, 1.54 mmol, 1.0 equiv) and tris(triphenylphosphine)rhodium(I) chloride (71 mg, 0.077 mmol, 5 mol%) in anhydrous toluene (100 mL), the expected product *M*(*R*,*R*)-**6** was obtained as an off white solid (652 mg, 1.12 mmol, 72%). As expected, the NMR was analogous to that reported above for *P*(*S*,*S*)-**6**. Mp: 290-292 °C.

^{1}H NMR (400 MHz, $CDCl_3$) δ_H 8.13 (d, J = 2.0 Hz, 2H, H1), 7.90 (dd, J = 8.4, 2.0 Hz, 2H, H3), 7.36 – 7.21 (m, 4H, H17,18), 7.14 (tt, J = 7.4, 1.4 Hz, 2H, H16), 7.10 – 7.03 (m, 4H, H4,15), 6.79 (dt, J = 7.4, 1.4 Hz, 2H, H14), 5.34 (q, J = 6.7 Hz, 2H, H6), 3.70 (s, 6H, H19), 1.00 (d, J = 6.7 Hz, 6H, H12). ^{13}C NMR (101 MHz, $CDCl_3$) δ_C 166.6, 157.8, 138.6, 137.7, 137.5, 131.0, 130.9, 130.8, 129.3, 128.1, 128.0, 127.1, 124.9, 123.1, 122.4, 119.4, 73.5, 51.8, 18.7. ν_{max} (cm^{-1}): 3054, 2985, 2949, 1709, 1260. HR-MS (ESI) *m/z* calculated for $C_{38}H_{31}O_6$ $[M+H]^+$ 583.2121, found 583.2094.

Synthesis of *P*(*S,S*)-S2

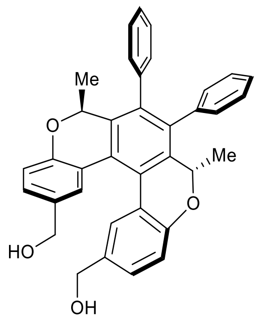

Compound *P*(*S,S*)-**6** (375 mg, 0.64 mmol, 1 equiv) was dissolved in anhydrous THF (6.5 mL). The resulting solution was cooled to 0 °C before $LiAlH_4$ (146 mg, 3.86 mmol, 6 equiv) was added in one portion. After 1 h the reaction was quenched *via* the Fieser workup. The reaction mixture was diluted in THF (6.5 mL) and cooled to 0 °C followed by sequential addition of H_2O (146 μL), 15% aq NaOH (146 μL) and H_2O (438 μL). The resultant mixture was warmed to RT and allowed to stir for 15 min. Na_2SO_4 was then added and left to stir for a further 15 min. Finally, the reaction mixture was filtered and concentrated *in vacuo* to yield the desired product *P*(*S,S*)-**S2** as a white solid (324 mg, 0.62 mmol, 96%). The product was subsequently reacted without further purification.

^{1}H NMR (500 MHz, $CDCl_3$) δ_H 7.56 (d, *J* = 1.4 Hz, 2H, 2 x Ar-CH), 7.31 – 7.24 (m, 4H, 4 x Ar-CH), 7.17 – 7.09 (m, 4H, 4 x Ar-CH), 7.05 (t, *J* = 7.6 Hz, 2H, 2 x Ar-CH), 7.00 (d, *J* = 8.1 Hz, 2H, 2 x Ar-CH), 6.79 (d, *J* = 7.6, 2H, 2 x Ar-CH), 5.28 (q, *J* = 6.7 Hz, 2H, 2 x CH), 4.44 (d, *J* = 11.7 Hz, 2H, 2 x CH-*H*), 4.37 (d, *J* = 11.7 Hz, 2H, 2 x CH-*H*), 0.97 (d, *J* = 6.7 Hz, 6H, 2 x CH_3). ^{13}C NMR (126 MHz, $CDCl_3$) δ_C 153.2, 138.9, 137.9, 137.3, 133.2, 131.0, 129.4, 129.3, 129.3, 128.0, 127.9, 126.9, 125.5, 123.1, 119.4, 73.0, 65.2, 18.5. ν_{max} (cm^{-1}): 3303, 3057, 2982, 2927, 2874, 1603, 1441, 1221. HR-MS (ESI) *m/z* calculated for $C_{36}H_{30}O_4Na$ [M+Na]$^+$ 549.2042, found 549.2033.

Synthesis of *M*(*R,R*)-S2

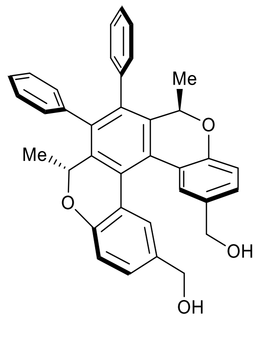

Following the procedure described above with *M*(*R,R*)-**6** (582 mg, 1.00 mmol, 1.0 equiv) and $LiAlH_4$ (228 mg, 6.00 mmol, 6 equiv) in anhydrous THF (10 mL), the expected product *M*(*R,R*)-

S2 was obtained as a white solid (495 mg, 0.94 mmol, 94%). As expected, the NMR was analogous to that reported above for *P*(*S,S*)-**S2**.

^{1}H NMR (400 MHz, $CDCl_3$) δ_H 7.55 (d, *J* = 1.4 Hz, 2H, 2 x Ar-CH), 7.32 – 7.26 (m, 4H, 4 x Ar-CH), 7.17 – 7.10 (m, 4H, 4 x Ar-CH), 7.05 (t, *J* = 7.6 Hz, 2H, 2 x Ar-CH), 7.00 (d, *J* = 8.1 Hz, 2H, 2 x Ar-CH), 6.78 (dt, *J* = 7.6, 1.5 Hz, 2H, 2 x Ar-CH), 5.27 (q, *J* = 6.7 Hz, 2H, 2 x CH), 4.45 (d, *J* = 11.7 Hz, 2H, 2 x CH-*H*), 4.38 (d, *J* = 11.7 Hz, 2H, 2 x CH-*H*), 0.96 (d, *J* = 6.7 Hz, 6H, 2 x CH_3). ^{13}C NMR (126 MHz, $CDCl_3$) δ_C 153.2, 138.9, 137.9, 137.3, 133.2, 131.0, 129.4, 129.3, 129.2, 128.0, 127.9, 126.9, 125.5, 123.1, 119.4, 73.1, 65.2, 18.5. ν_{max} (cm^{-1}): 3324, 3054, 2978, 2927, 2871, 1602, 1441, 1221. HR-MS (ESI) *m/z* calculated for $C_{36}H_{30}O_4Na$ [M+Na]$^+$ 549.2042, found 549.2028.

Synthesis of *P*(*S,S*)-7

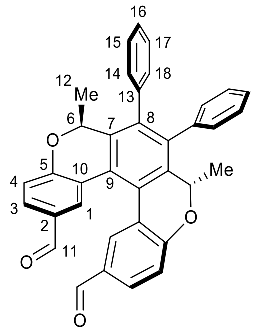

Oxalyl chloride (514 µL, 6.08 mmol, 4.0 equiv) was dissolved in anhydrous CH_2Cl_2 (14 mL) under an N_2 atmosphere and cooled to -78 °C. DMSO (867 µL,12.2 mmol, 8 equiv) was then added dropwise. To this, a solution of *P*(*S,S*)-**6** (803 mg, 1.52 mmol, 1 equiv) in anhydrous CH_2Cl_2/ DMSO (3.6 mL/ 1.2 mL) was added dropwise. The reaction mixture was stirred for 15 min before anhydrous Et_3N (3.82 mL, 27.4 mmol, 18 equiv) was added slowly. The mixture was stirred for a further 5 min at -78 °C and subsequently left to warm to RT over 1 h with stirring. Upon completion of the reaction, the mixture was diluted in H_2O (20 mL) and separated. The aqueous phase was extracted with CH_2Cl_2 (5 x 20 mL) and the combined organic extracts were dried over Na_2SO_4, filtered and concentrated *in vacuo*. The product was purified by flash chromatography (petroleum ether:EtOAc, (9:1 – 8:2 *v/v* ratio) yielding *P*(*S,S*)-**7** as a pale yellow solid (639 mg, 1.22 mmol, 80%).

^{1}H NMR (500 MHz, $CDCl_3$) δ_H 9.62 (s, 2H, H11), 7.89 (d, *J* = 1.9 Hz, 2H, H1), 7.75 (dd, *J* = 8.3, 1.9 Hz, 2H, H3), 7.32 (t, *J* = 7.5, 2H, H17), 7.29 – 7.26 (m, 2H, H18), 7.21 – 7.12 (m, 4H, H4,16), 7.07 (t, *J* = 7.5, 2H, H15), 6.78 (d, *J* = 7.5, 2H, H14), 5.39 (q, *J* = 6.7 Hz, 2H, H6), 1.00 (d, *J* = 6.7 Hz, 6H, H12). ^{13}C NMR (126 MHz, $CDCl_3$) δ_C 190.4, 159.5, 138.9, 138.0, 137.3, 131.2, 131.1, 130.9, 130.3, 129.2, 128.2, 128.1, 127.2, 124.6, 123.1, 120.5, 73.9, 18.8. ν_{max} (cm^{-1}): 2983, 2839,

1693, 1598, 1226. HR-MS (ESI) *m/z* calculated for $C_{36}H_{26}O_4Na$ $[M+Na]^+$ 545.1729, found 545.1721.

Synthesis of *M*(*R,R*)-7

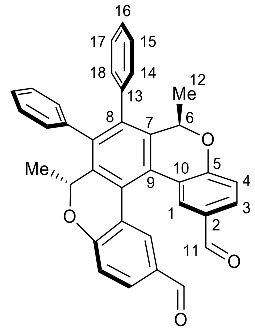

Following the procedure described above with oxalyl chloride (345 μL, 4.08 mmol, 4.0 equiv) dissolved in CH_2Cl_2 (9 mL), DMSO (580 μL, 8.16 mmol, 8 equiv), *M*(*R,R*)-**6** (537 mg, 1.02 mmol, 1 equiv) in CH_2Cl_2/ DMSO (2.4 mL/ 0.8 mL) and Et_3N (2.56 mL, 18.4 mmol, 18 equiv), the expected product *M*(*R,R*)-**7** was obtained as a pale yellow solid (420 mg, 0.80 mmol, 79%). As expected, the NMR was analogous to that reported above for *P*(*S,S*)-**7**.

^{1}H NMR (500 MHz, $CDCl_3$) δ_H 9.62 (s, 2H, H11), 7.89 (d, *J* = 1.9 Hz, 2H, H1), 7.75 (dd, *J* = 8.3, 1.9 Hz, 2H, H3), 7.32 (td, *J* = 7.5, 1.4 Hz, 2H, H17), 7.29 – 7.26 (m, 2H, H18), 7.19 (d, *J* = 8.3 Hz, 2H, H4), 7.15 (tt, *J* = 7.5, 1.4 Hz, 2H, H16), 7.08 (td, *J* = 7.5, 1.4 Hz, 2H, H15), 6.78 (dt, *J* = 7.5, 1.4 Hz, 2H, H14), 5.39 (q, *J* = 6.7 Hz, 2H, H6), 1.00 (d, *J* = 6.7 Hz, 6H, H12). ^{13}C NMR (126 MHz, $CDCl_3$) δ_C 190.4, 159.5, 138.9, 138.0, 137.3, 131.2, 131.1, 130.9, 130.3, 129.2, 128.2, 128.1, 127.2, 124.6, 123.1, 120.5, 73.9, 18.8. ν_{max} (cm^{-1}): 2927, 2853, 1695, 1599, 1264. HR-MS (ESI) *m/z* calculated for $C_{36}H_{26}O_4Na$ $[M+Na]^+$ 545.1729, found 545.1718.

Synthesis of (*P,P*)-8

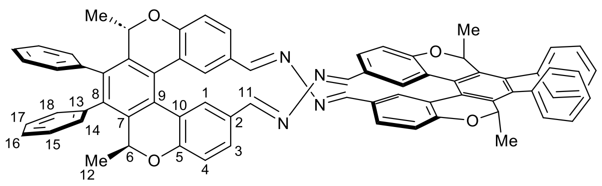

Compound *P*(*S,S*)-**7** (20 mg, 0.038 mmol, 1.0 equiv) was dissolved in CH_2Cl_2 (3 mL). To this, hydrazine monohydrate (2.0 μL, 0.038 mmol, 1.0 equiv) was added and the reaction mixture was left to stir at room temperature. After 2 h, the reaction mixture was analysed by TLC (petroleum ether:EtOAc, (7:3 *v/v* ratio)):

- If a single spot with R_f 0.58 was present, the mixture was subsequently concentrated and dried *in vacuo* yielding *P*(*S,S*)-**8** as an off-white amorphous solid (19 mg, 0.018 mmol, 95%). No further purification was carried out.
- If unreacted aldehyde (R_f 0.36) was detected, 0.25 equiv of hydrazine monohydrate was added and the reaction mixture was stirred for an additional hour.
- If spots with R_f 0.13 or R_f 0.02 were detected, the mixture was dried *in vacuo* and dissolved in 0.8 mL of $CDCl_3$. The reaction mixture was transferred to a NMR tube and solid aldehyde *P*(*S,S*)-**7** was added directly in tube to achieve full conversion (reaction monitored by ^1H NMR).

^1H NMR (400 MHz, $CDCl_3$) δ_H 8.06 (s, 4H, H11), 7.93 (dd, *J* = 8.5, 1.9 Hz, 4H, H3), 7.50 (d, *J* = 1.8 Hz, 4H, H1), 7.35 – 7.23 (m, 8H, H17,18), 7.19 – 7.03 (m, 12H, H4,15,16), 6.81 (d, *J* = 7.5 Hz, 4H, H14), 5.33 (q, *J* = 6.6 Hz, 4H, H6), 1.05 (d, *J* = 6.7 Hz, 12H, H12). ^{13}C NMR (126 MHz, $CDCl_3$) δ_C 159.4, 156.4, 138.6, 137.7, 137.6, 132.6, 130.9, 129.3, 128.1, 128.0, 127.2, 127.1, 127.0, 124.7, 122.3, 120.2, 73.5, 18.7. ν_{max} (cm^{-1}): 2927, 2165, 1603, 1486, 1432, 1225. HR-MS (ESI) *m/z* calculated for $C_{72}H_{53}N_4O_4$ $[M+H]^+$ 1037.4067, found 1037.4050.

Synthesis of (*M,M*)-8

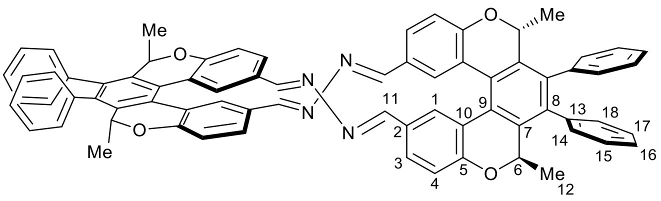

Following the procedure described above with *M*(*R,R*)-**7** (30 mg, 0.057 mmol, 1.0 equiv) and hydrazine monohydrate (3.0 µL, 0.057 mmol, 1.0 equiv) in CH_2Cl_2 (4.5 mL), the expected product (*M,M*)-**8** was obtained as an off-white solid (27 mg, 0.027 mmol, 93%). As expected, the NMR was analogous to that reported above for (*P,P*)-**8**.

^1H NMR (500 MHz, $CDCl_3$) δ_H 8.06 (s, 4H, H11), 7.93 (dd, *J* = 8.4, 1.9 Hz, 4H, H3), 7.50 (d, *J* = 1.9 Hz, 4H, H1), 7.34 – 7.23 (m, 8H, H17,18), 7.18 – 7.04 (m, 12H, H4,15,16), 6.81 (d, *J* = 7.5 Hz, 4H, H14), 5.33 (q, *J* = 6.6 Hz, 4H, H6), 1.05 (d, *J* = 6.7 Hz, 12H, H12). ^{13}C NMR (126 MHz, $CDCl_3$) δ_C 159.4, 156.4, 138.6, 137.7, 137.6, 132.6, 130.9, 129.3, 128.1, 128.0, 127.2, 127.1, 127.0, 124.7, 122.3, 120.2, 73.5, 18.7. ν_{max} (cm^{-1}): 2926, 2161, 1602, 1484, 1442, 1223. HR-MS (ESI) *m/z* calculated for $C_{72}H_{53}N_4O_4$ $[M+H]^+$ 1037.4067, found 1037.4064.

2. Supplementary NMR Spectra

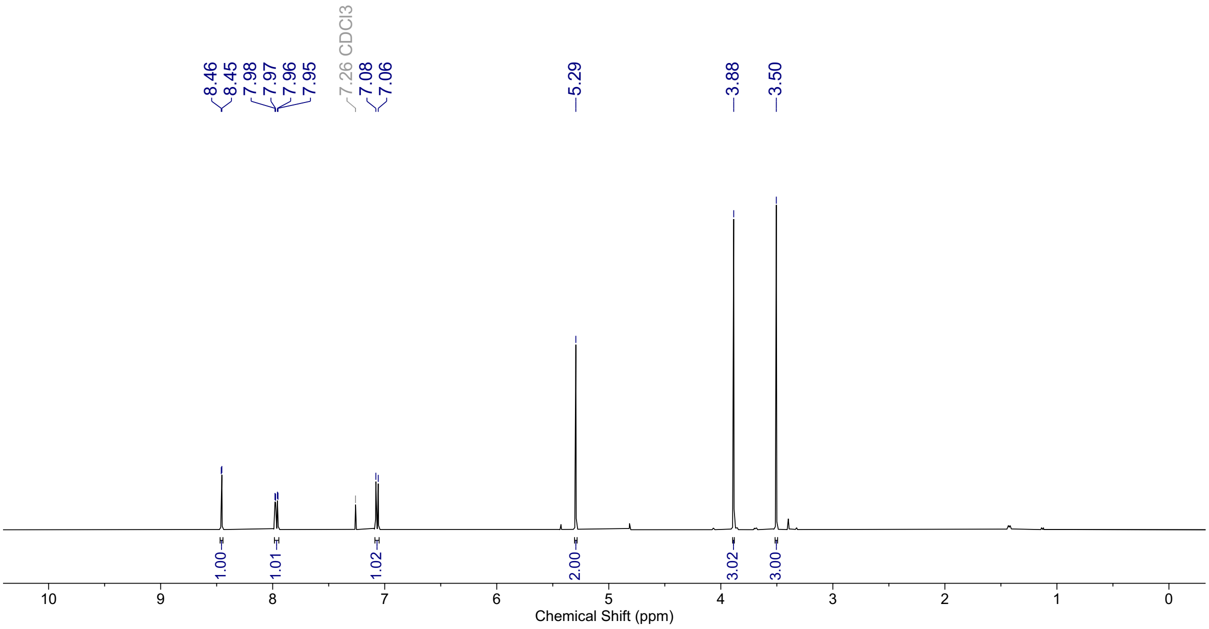

Supplementary Figure 2. ^{1}H NMR spectrum (400 MHz, 298 K) in $CDCl_3$ of 1.

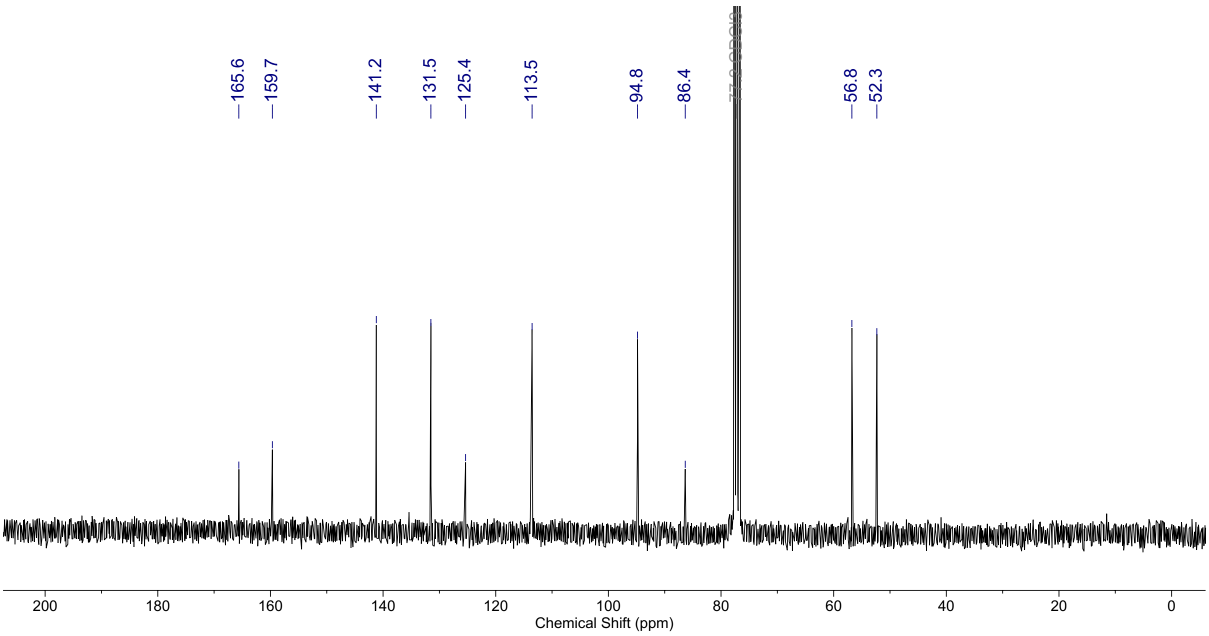

Supplementary Figure 3. ^{13}C NMR spectrum (75 MHz, 298 K) in $CDCl_3$ of 1.

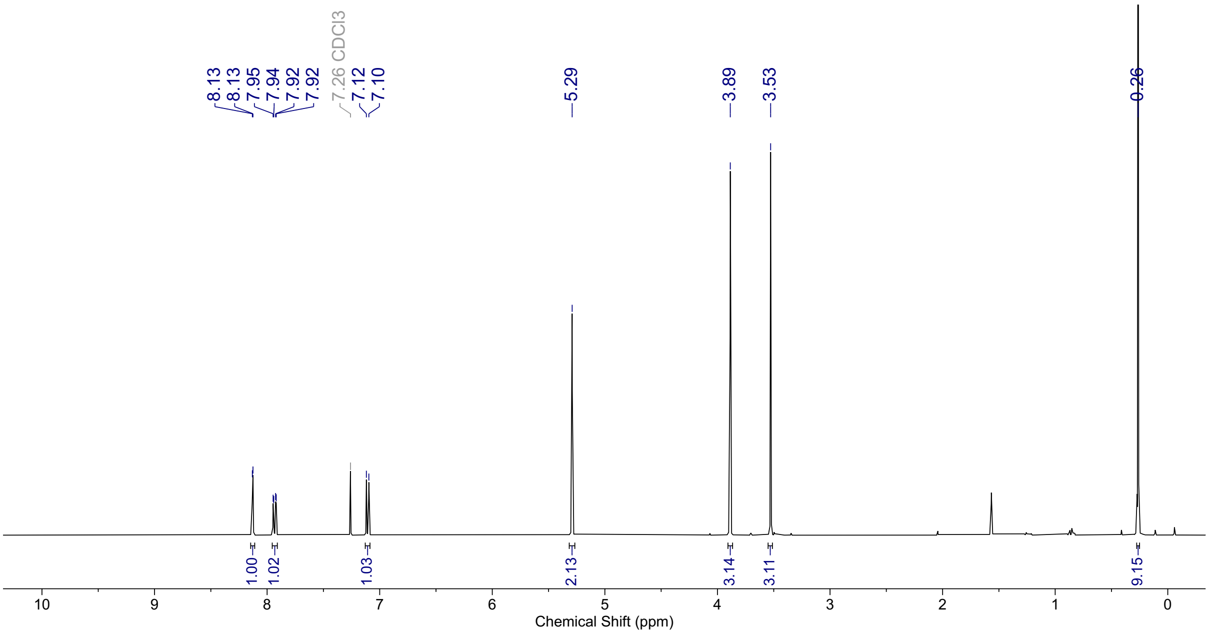

Supplementary Figure 4. ^{1}H NMR spectrum (400 MHz, 298 K) in $CDCl_3$ of S1.

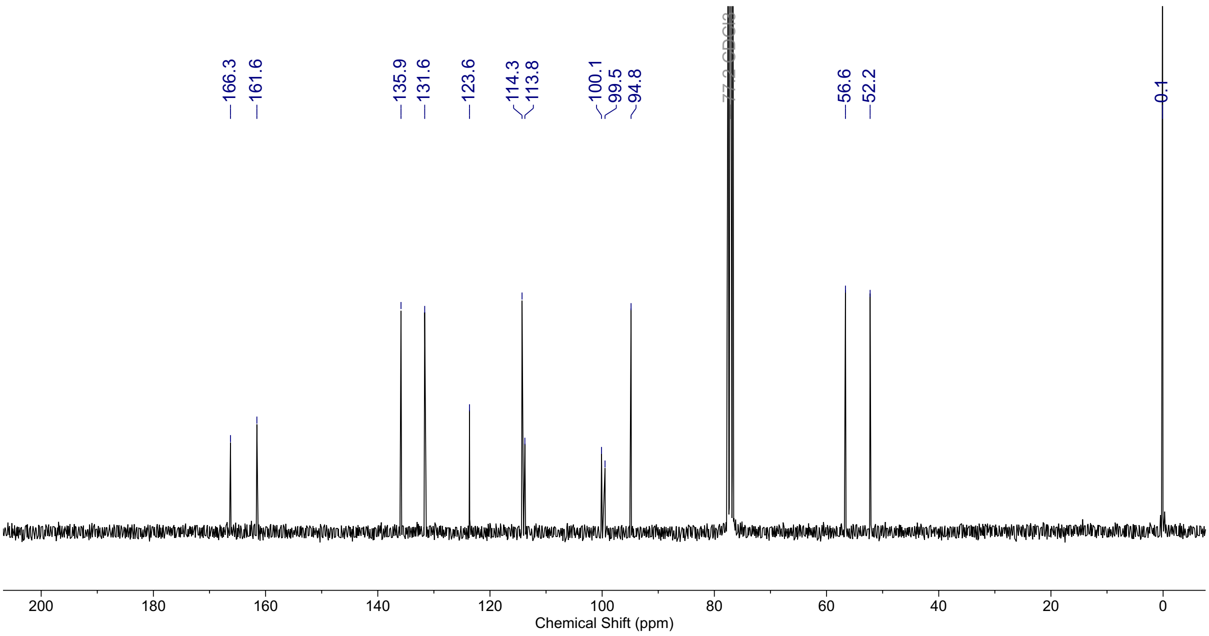

Supplementary Figure 5. ^{13}C NMR spectrum (75 MHz, 298 K) in $CDCl_3$ of S1.

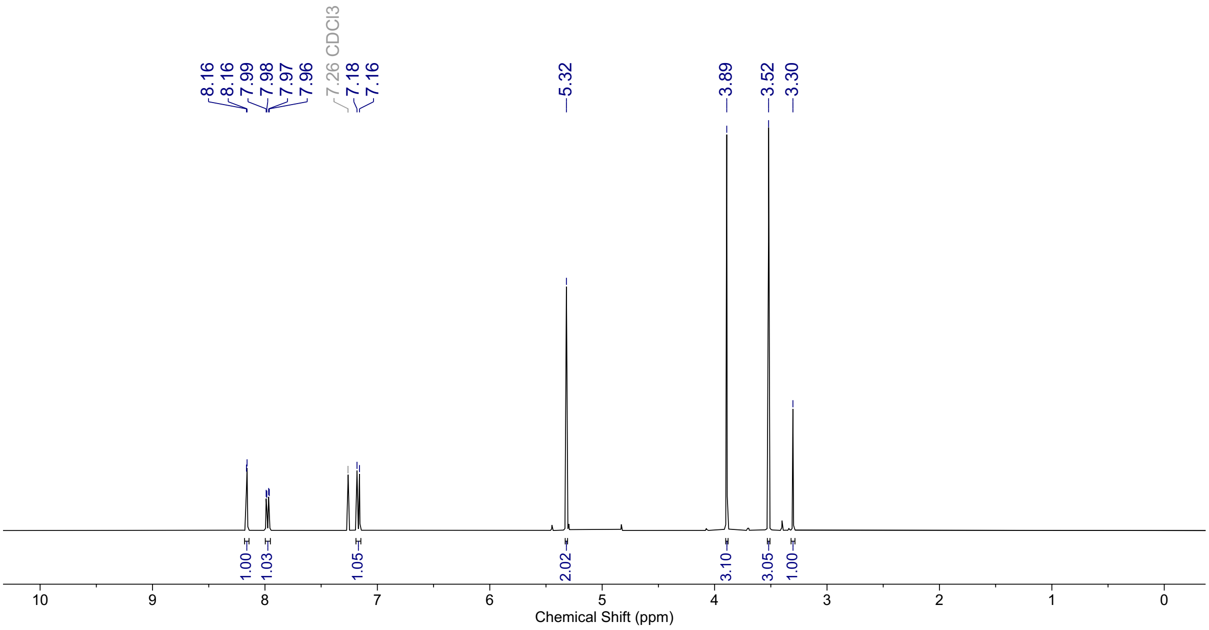

Supplementary Figure 6. ^{1}H NMR spectrum (400 MHz, 298 K) in $CDCl_3$ of 2.

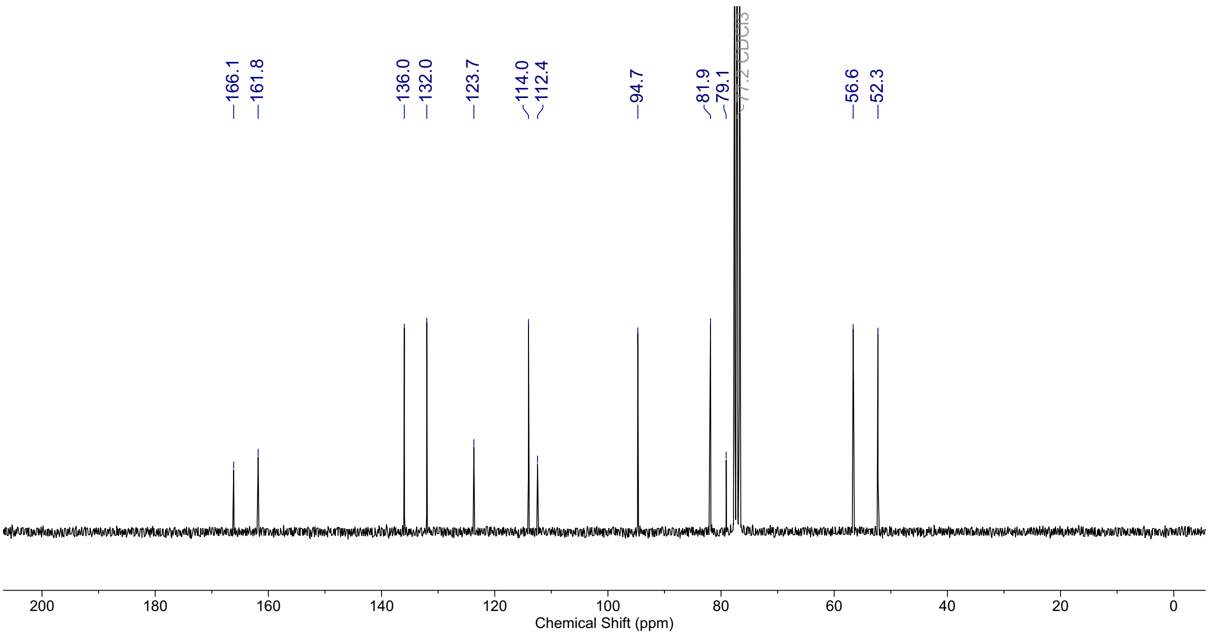

Supplementary Figure 7. ^{13}C NMR spectrum (75 MHz, 298 K) in $CDCl_3$ of 2.

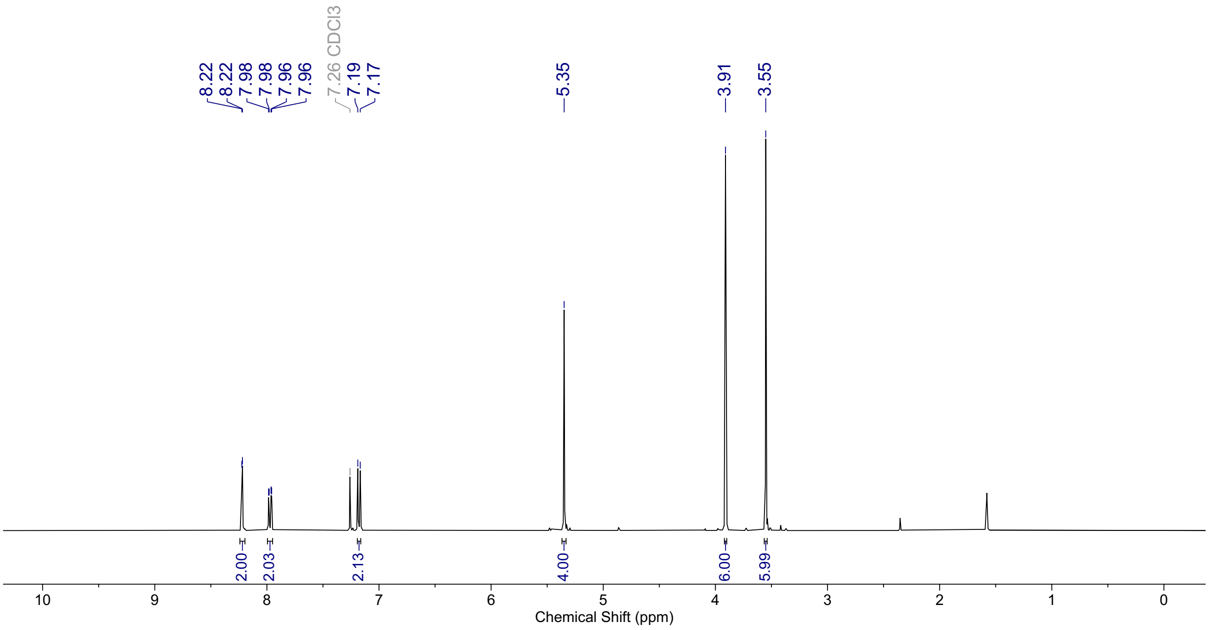

Supplementary Figure 8. ^{1}H NMR spectrum (400 MHz, 298 K) in $CDCl_3$ of 3.

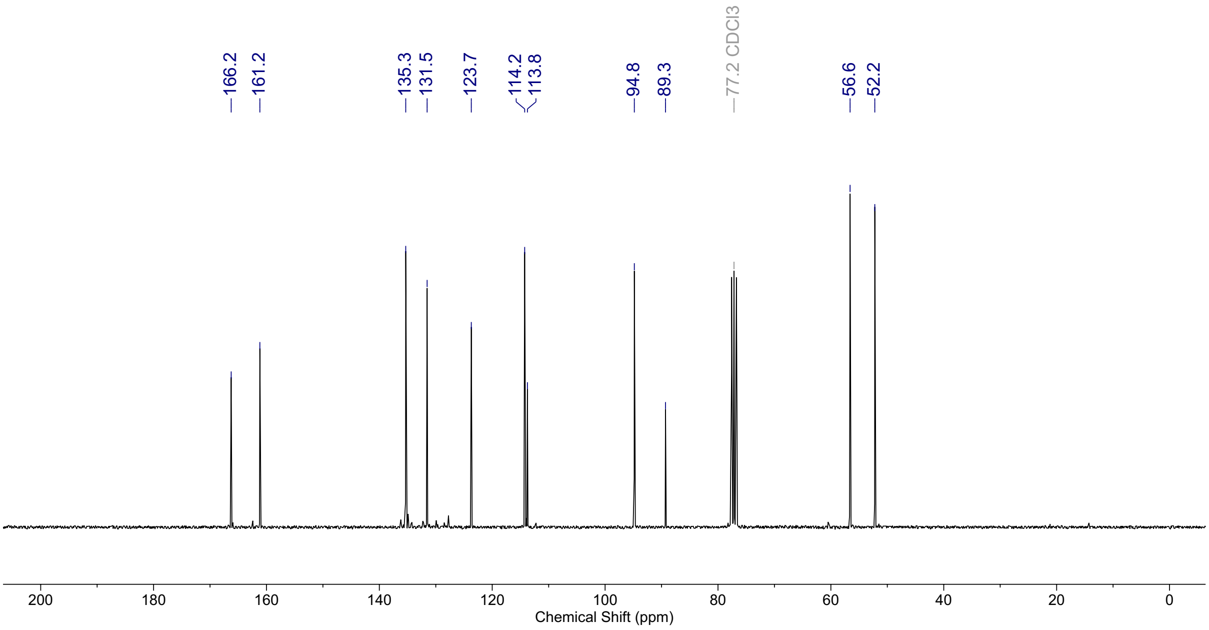

Supplementary Figure 9. ^{13}C NMR spectrum (75 MHz, 298 K) in $CDCl_3$ of 3.

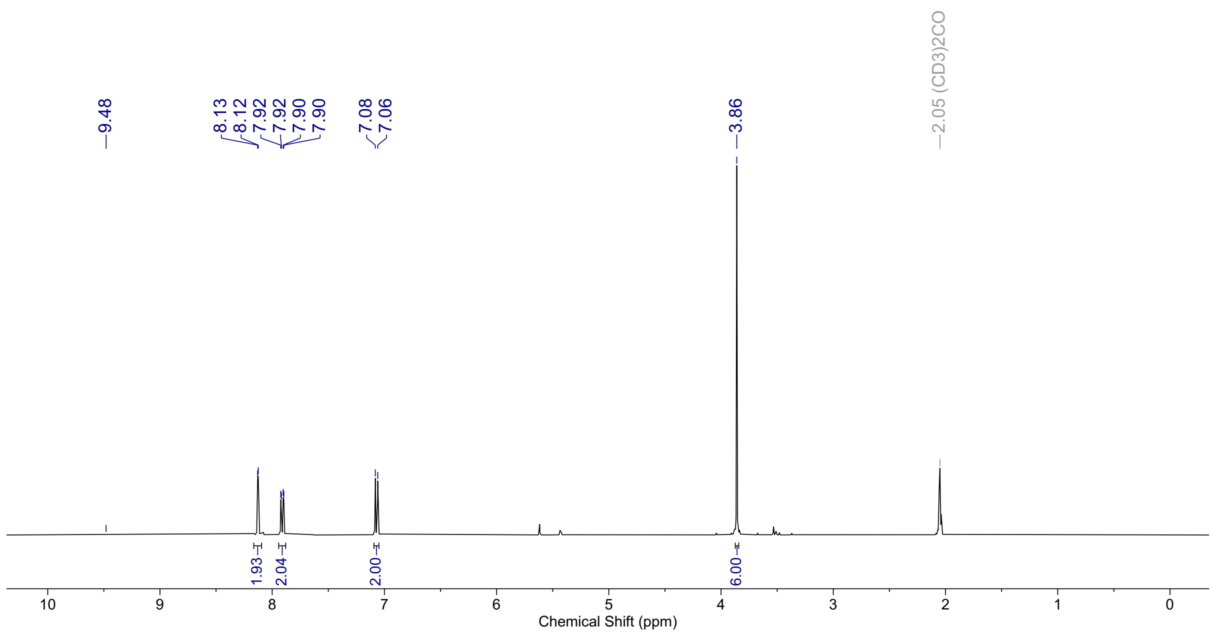

Supplementary Figure 10. ^{1}H NMR spectrum (400 MHz, 298 K) in acetone-d_6 of 4.

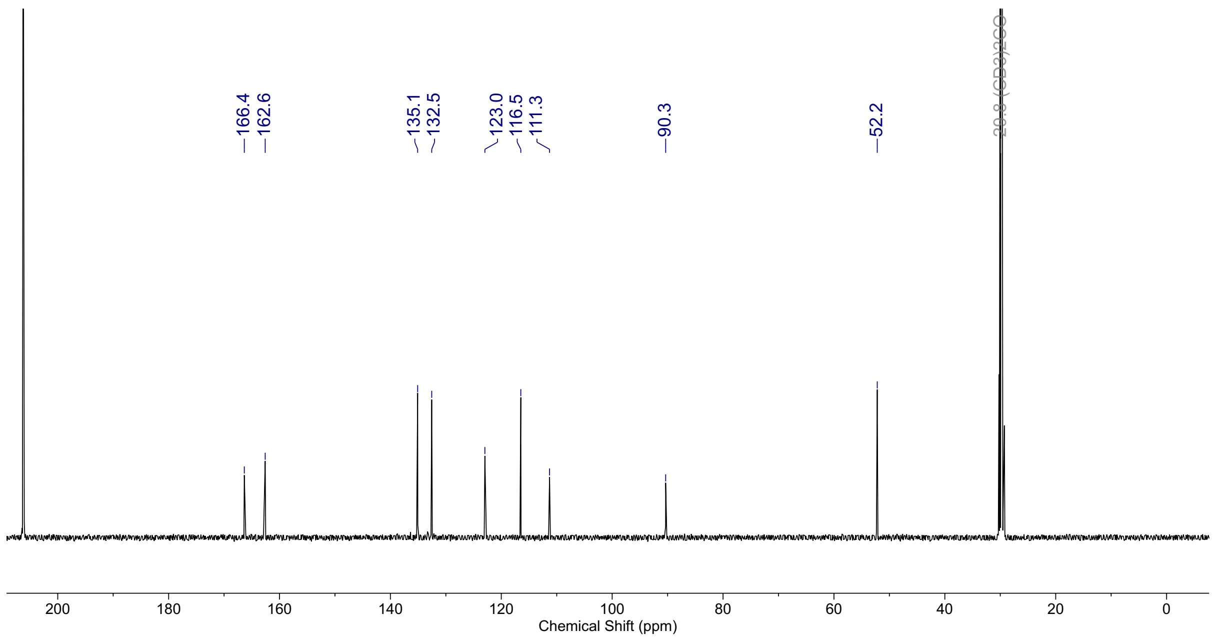

Supplementary Figure 11. ^{13}C NMR spectrum (101 MHz, 298 K) in acetone-d_6 of 4.

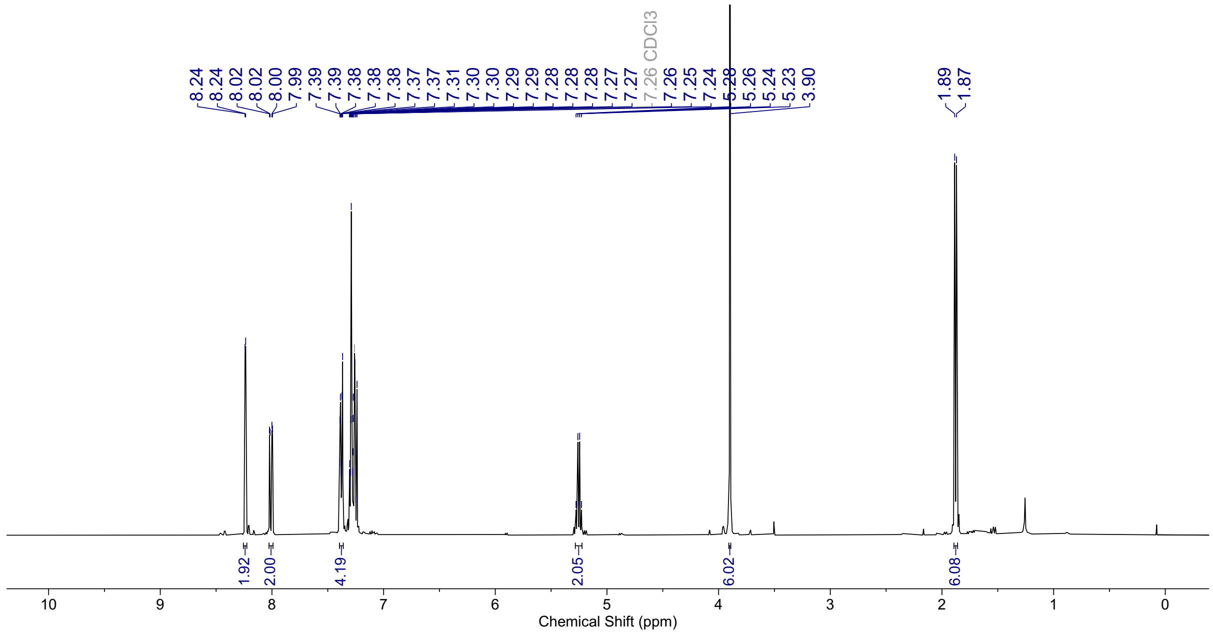

Supplementary Figure 12. ^{1}H NMR spectrum (400 MHz, 298 K) in $CDCl_3$ of (*S,S*)-5.

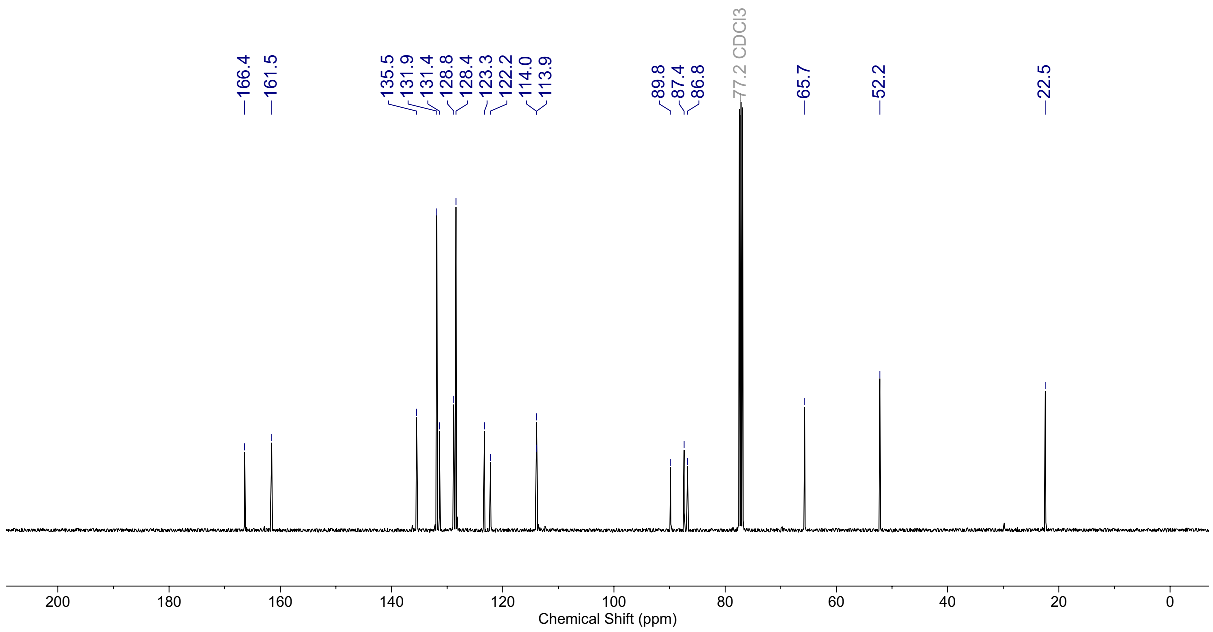

Supplementary Figure 13. ^{13}C NMR spectrum (101 MHz, 298 K) in $CDCl_3$ of (*S,S*)-5.

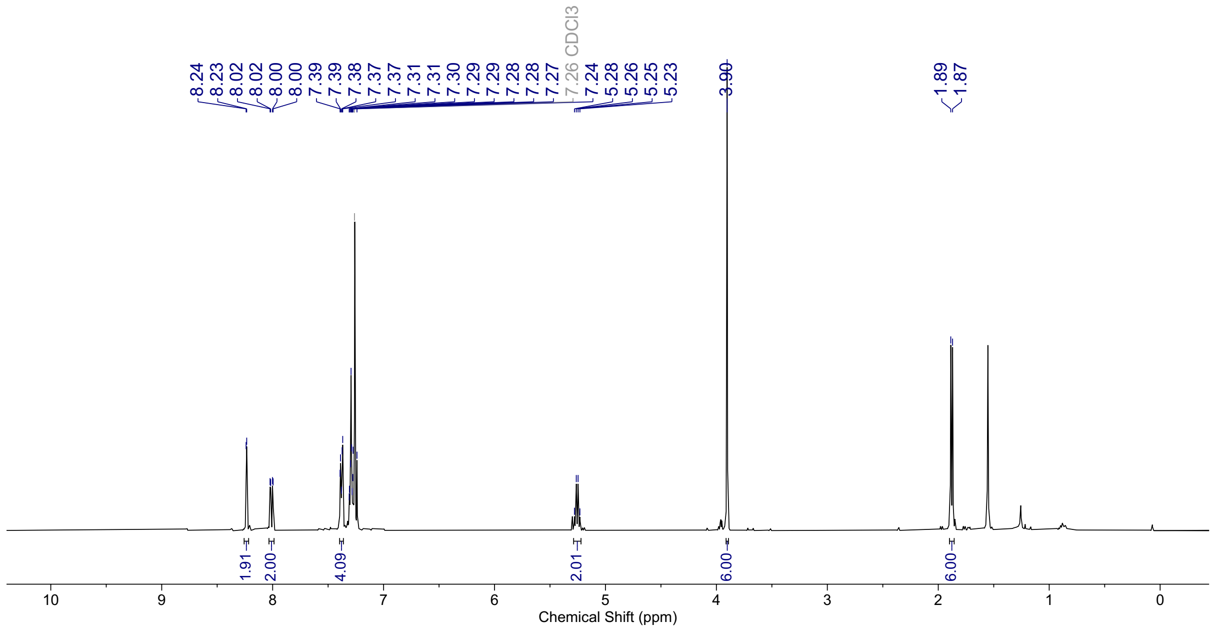

Supplementary Figure 14. ^{1}H NMR spectrum (400 MHz, 298 K) in $CDCl_3$ of (*R,R*)-5.

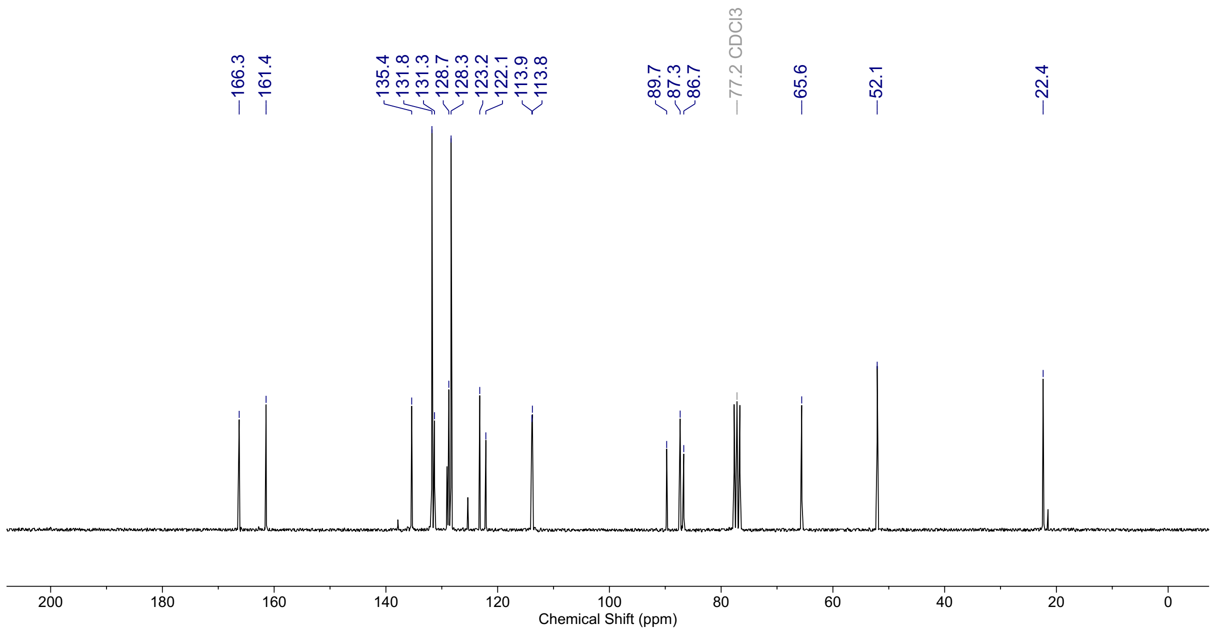

Supplementary Figure 15. ^{13}C NMR spectrum (75 MHz, 298 K) in $CDCl_3$ of (*R,R*)-5.

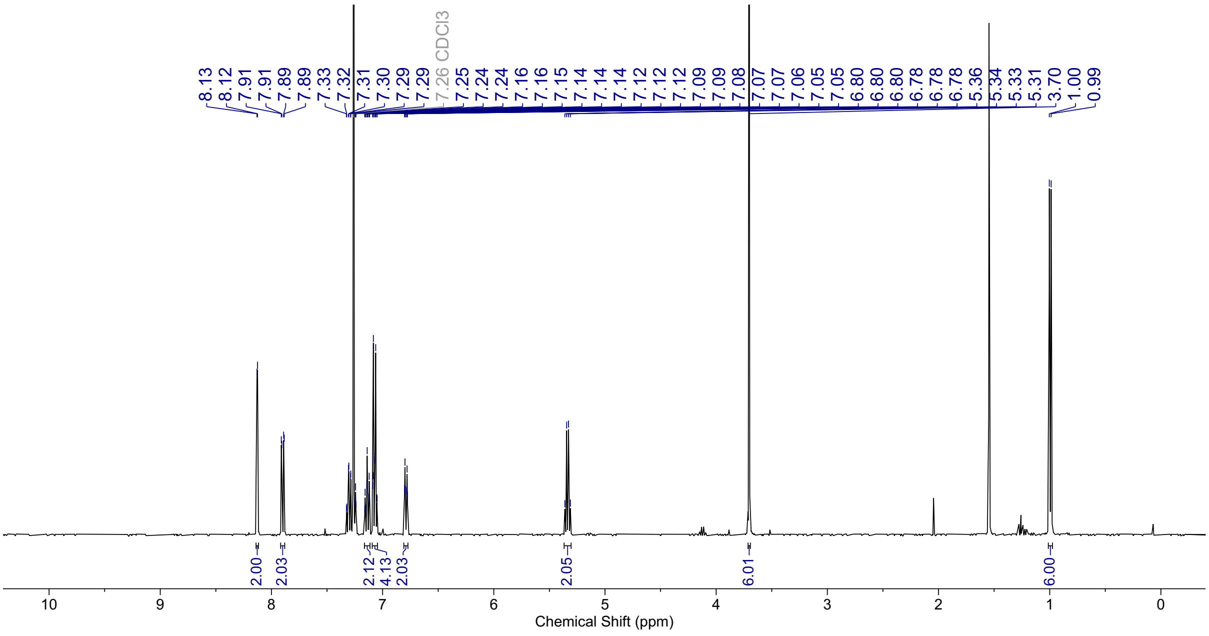

Supplementary Figure 16. ^{1}**H NMR spectrum (400 MHz, 298 K) in** $CDCl_3$ **of** ***P*(*S,S*)-6.**

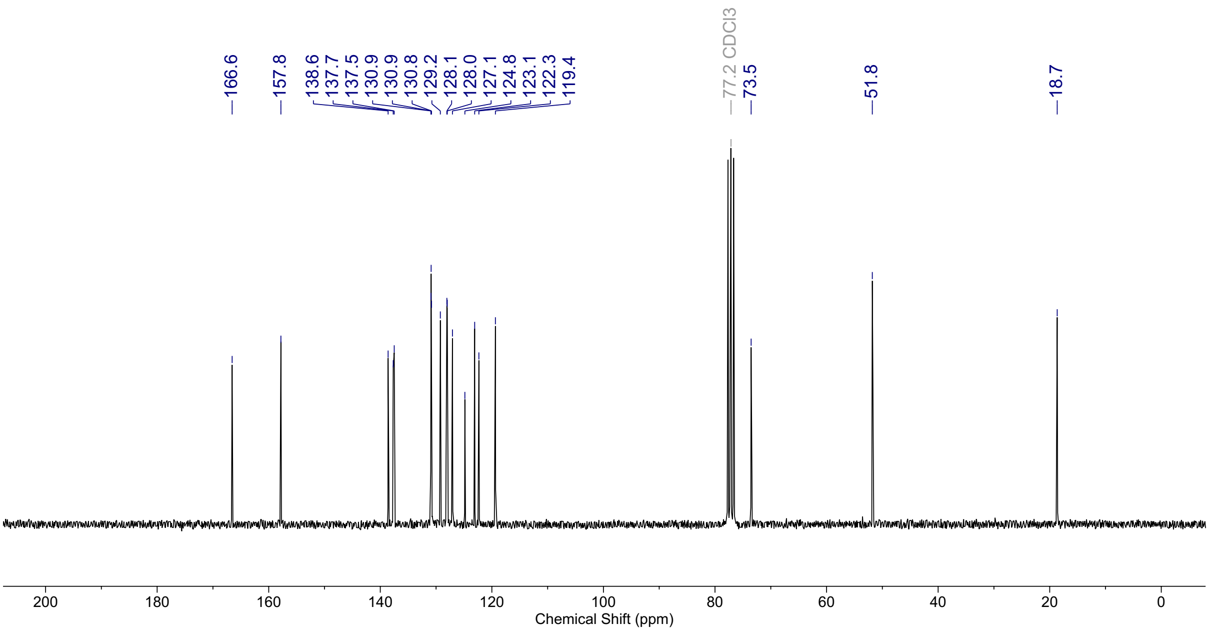

Supplementary Figure 17. ^{13}**C NMR spectrum (101 MHz, 298 K) in** $CDCl_3$ **of** ***P*(*S,S*)-6.**

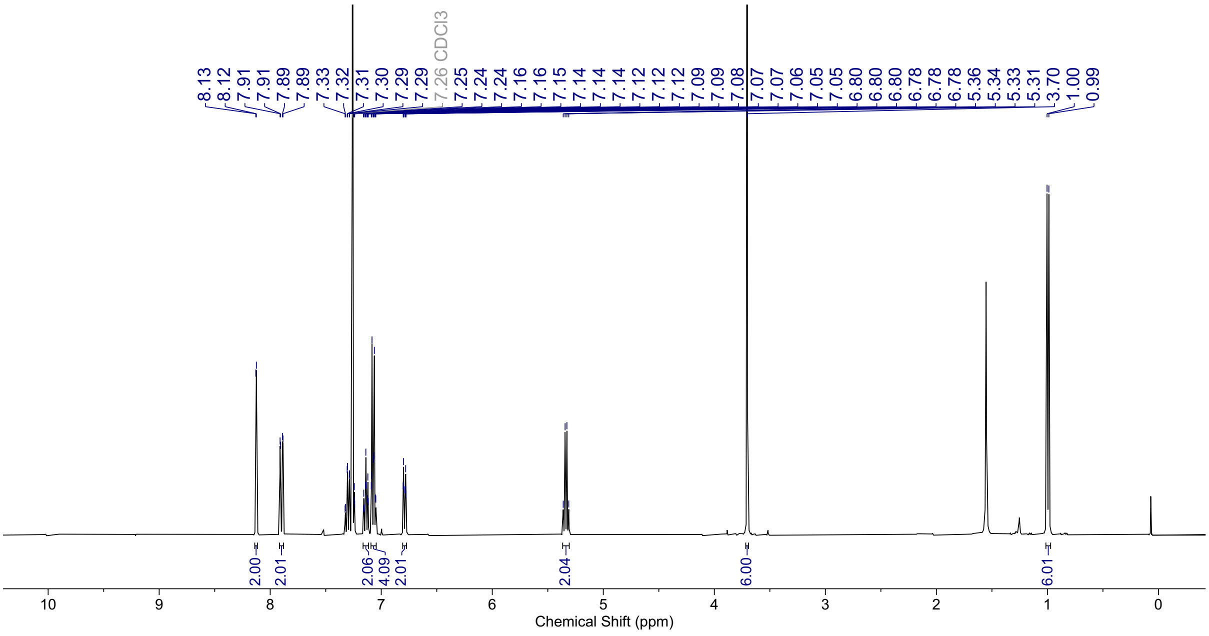

Supplementary Figure 18. ^{1}H NMR spectrum (400 MHz, 298 K) in $CDCl_3$ of *M*(*R,R*)-6.

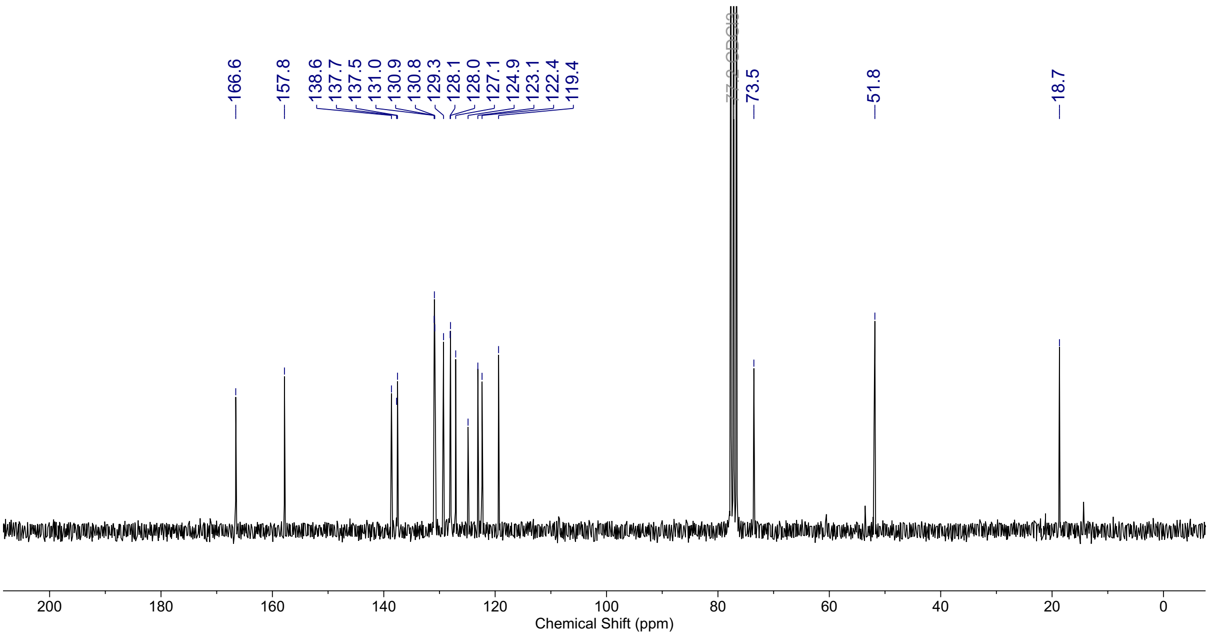

Supplementary Figure 19. ^{13}C NMR spectrum (101 MHz, 298 K) in $CDCl_3$ of *M*(*R,R*)-6.

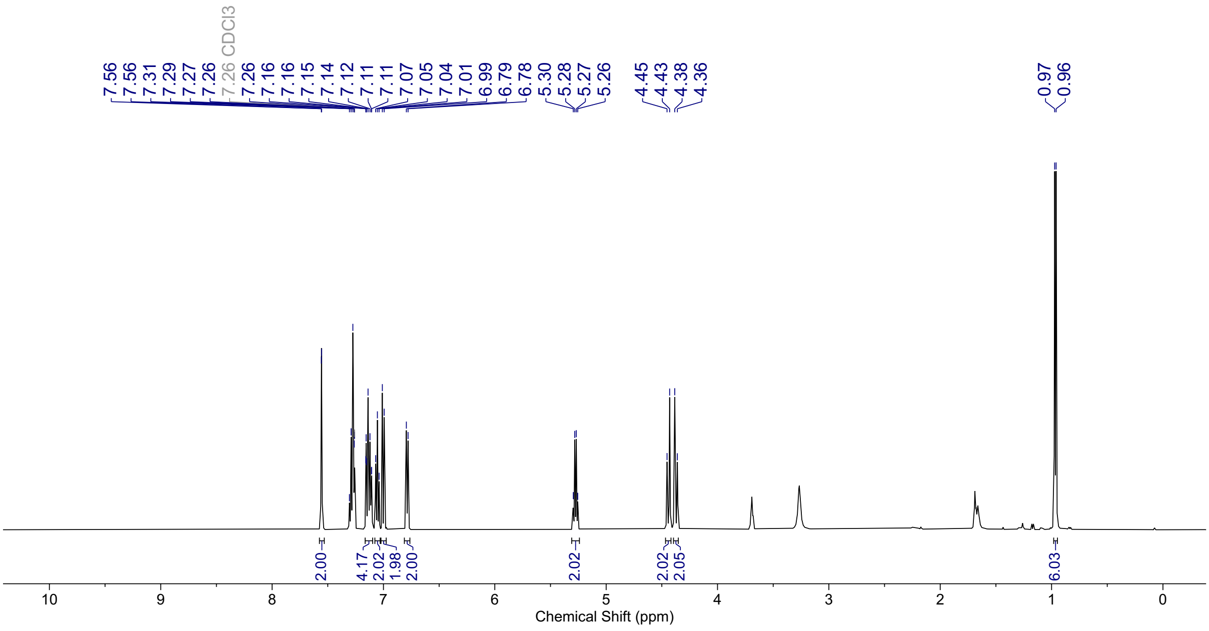

Supplementary Figure 20. **^{1}H NMR spectrum (500 MHz, 298 K) in $CDCl_3$ of *P*(*S,S*)-S2.**

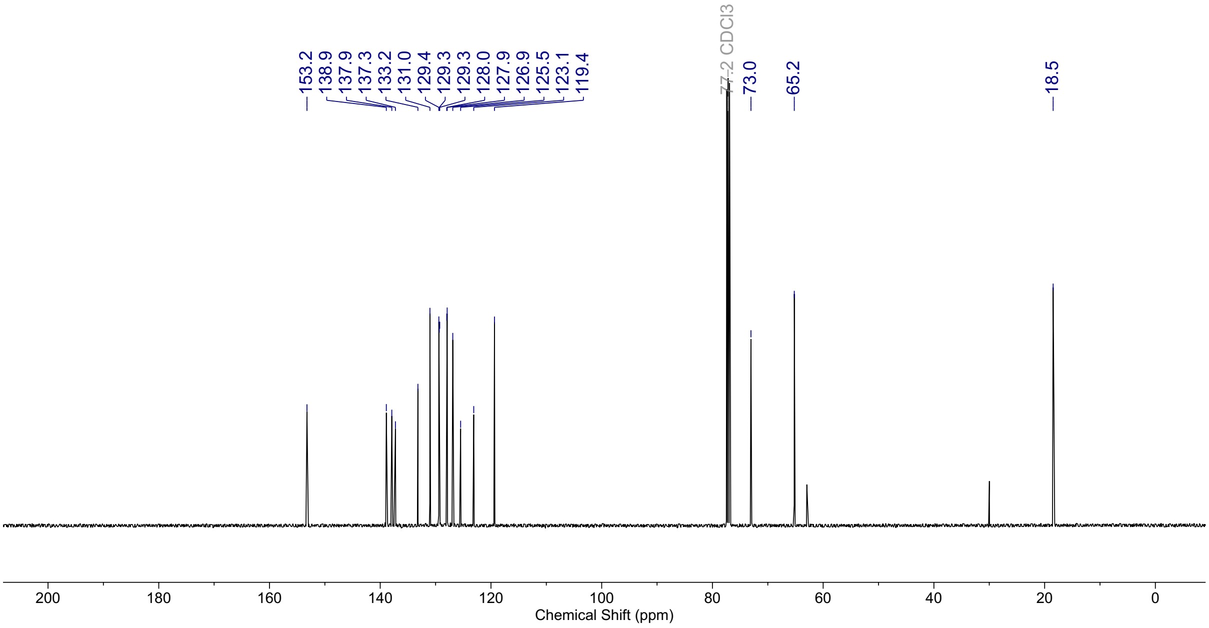

Supplementary Figure 21. **^{13}C NMR spectrum (126 MHz, 298 K) in $CDCl_3$ of *P*(*S,S*)-S2.**

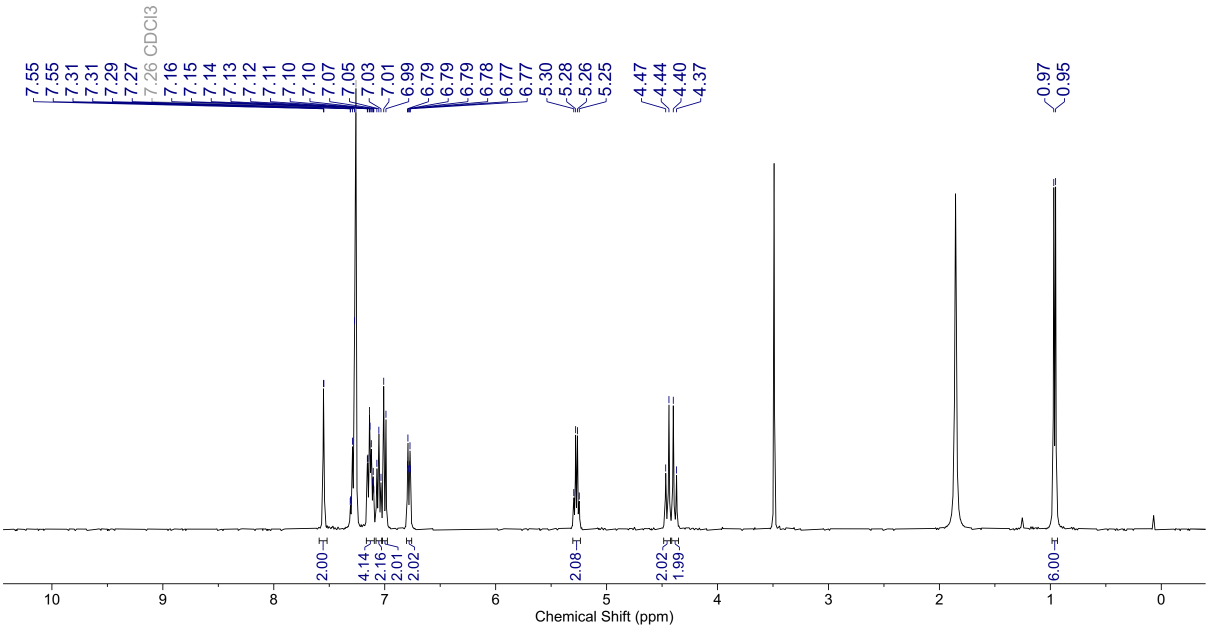

Supplementary Figure 22. ^{1}H NMR spectrum (400 MHz, 298 K) in $CDCl_3$ of *M*(*R,R*)-S2.

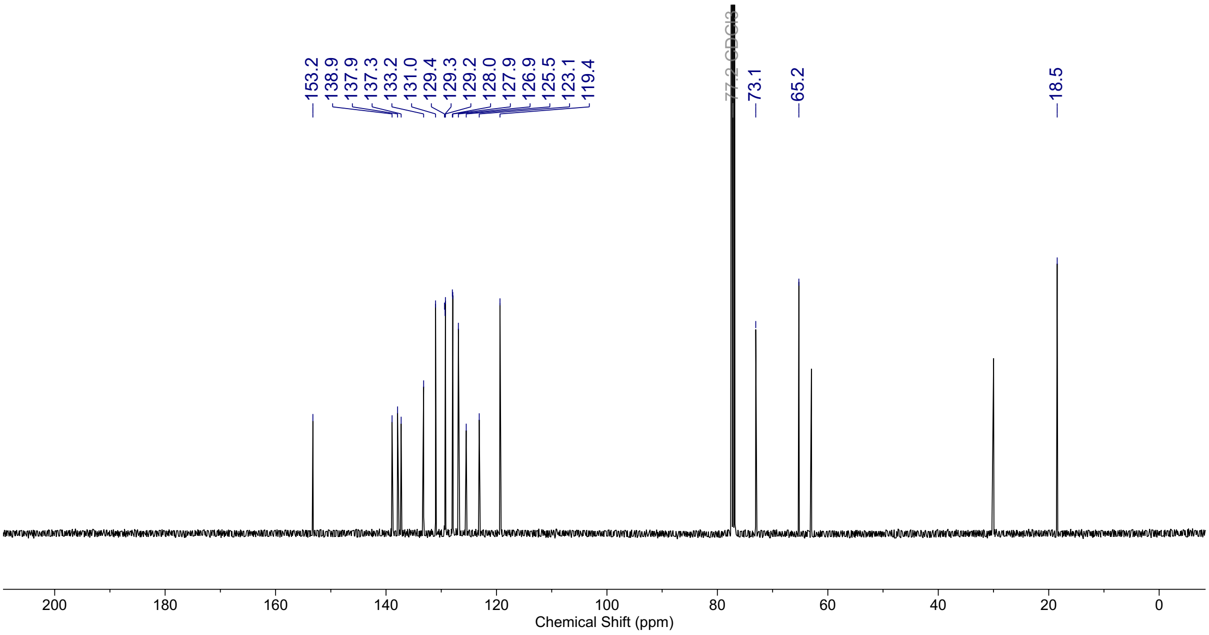

Supplementary Figure 23. ^{13}C NMR spectrum (126 MHz, 298 K) in $CDCl_3$ of *M*(*R,R*)-S2.

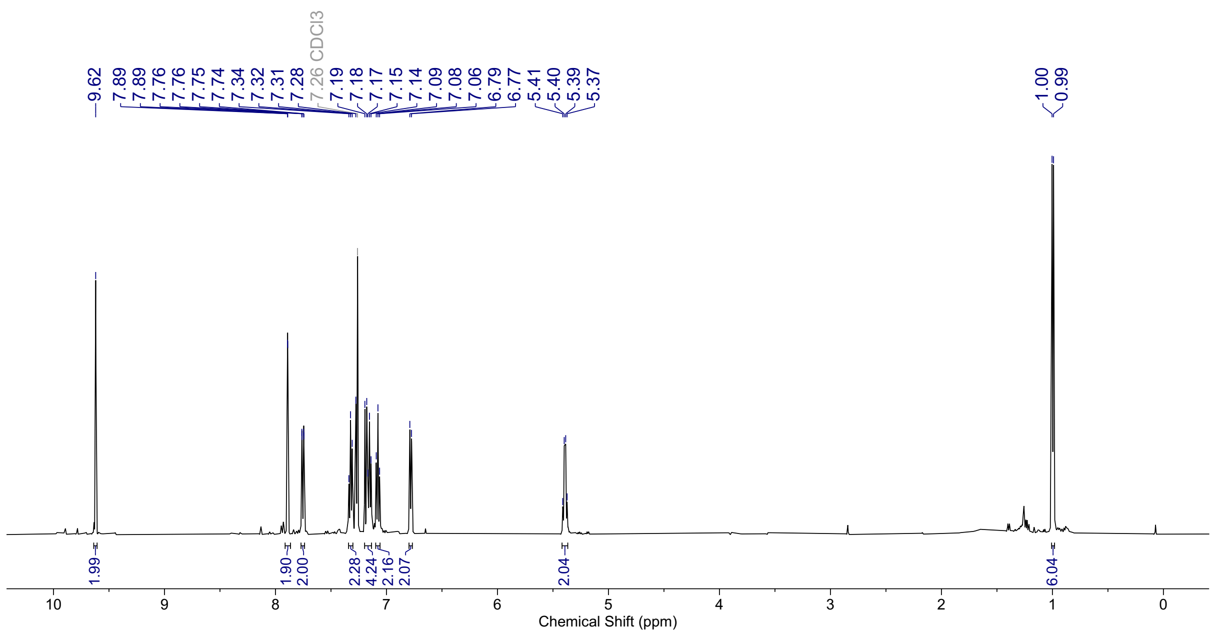

Supplementary Figure 24. ¹H NMR spectrum (500 MHz, 298 K) in CDCl₃ of *P*(*S,S*)-7.

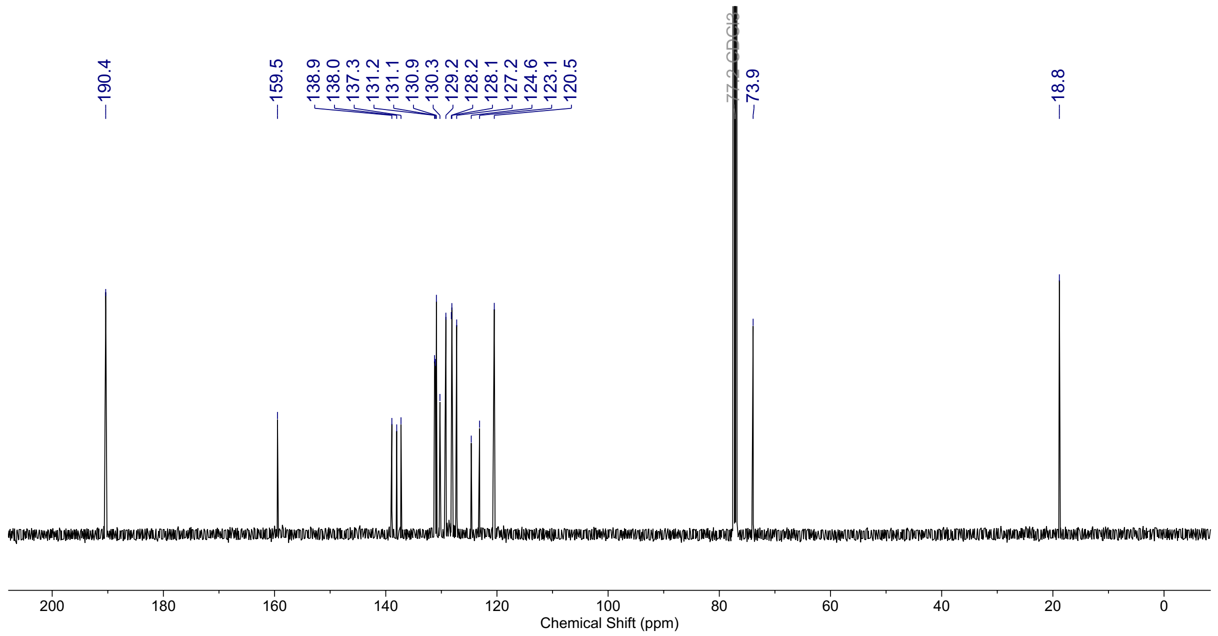

Supplementary Figure 25. ^{13}C NMR spectrum (126 MHz, 298 K) in $CDCl_3$ of *P*(*S,S*)-7.

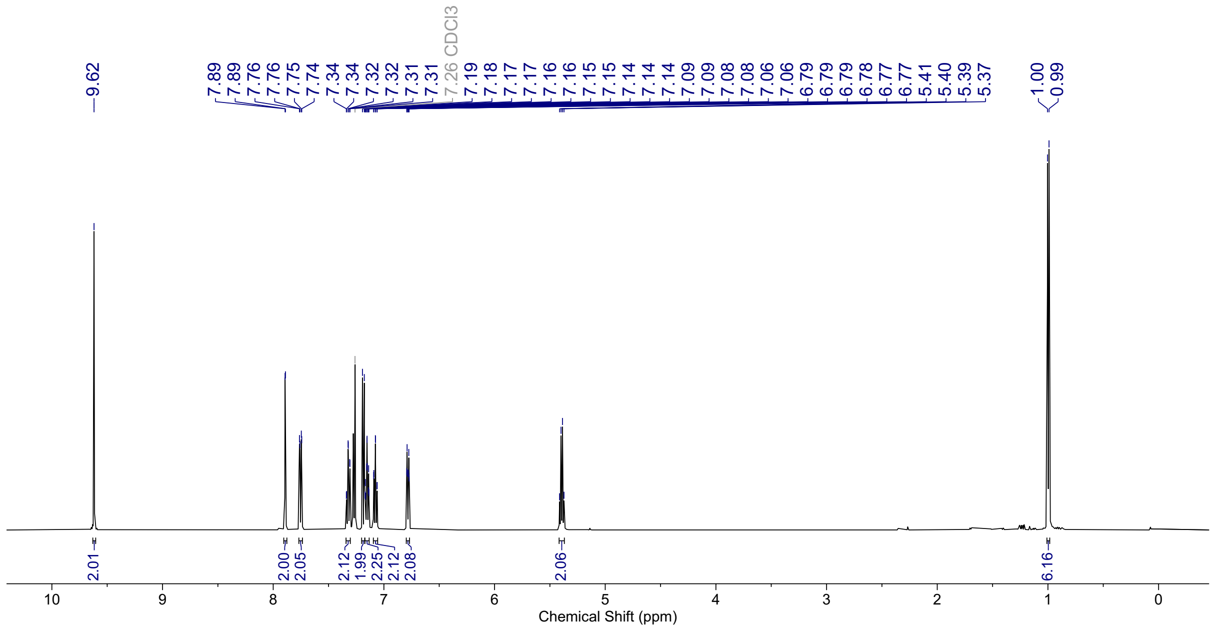

Supplementary Figure 26. ^{1}**H NMR spectrum (500 MHz, 298 K) in CDCl**$_{3}$ **of** ***M*(*R,R*)-7.**

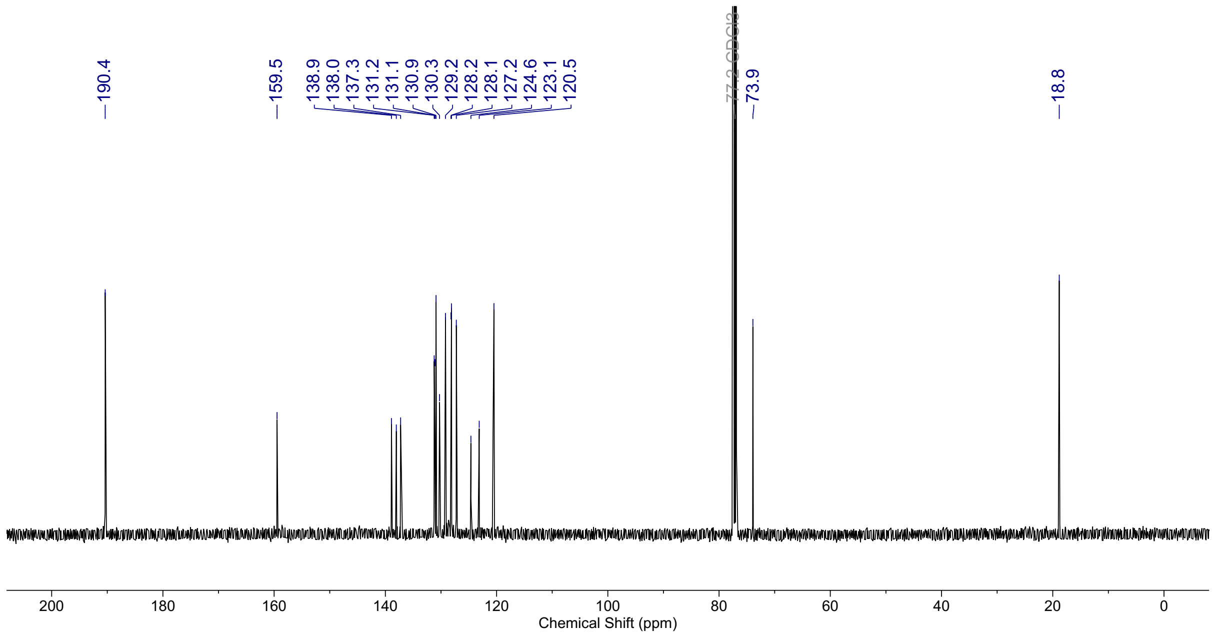

Supplementary Figure 27. ^{13}**C NMR spectrum (126 MHz, 298 K) in CDCl**$_{3}$ **of** ***M*(*R,R*)-7.**

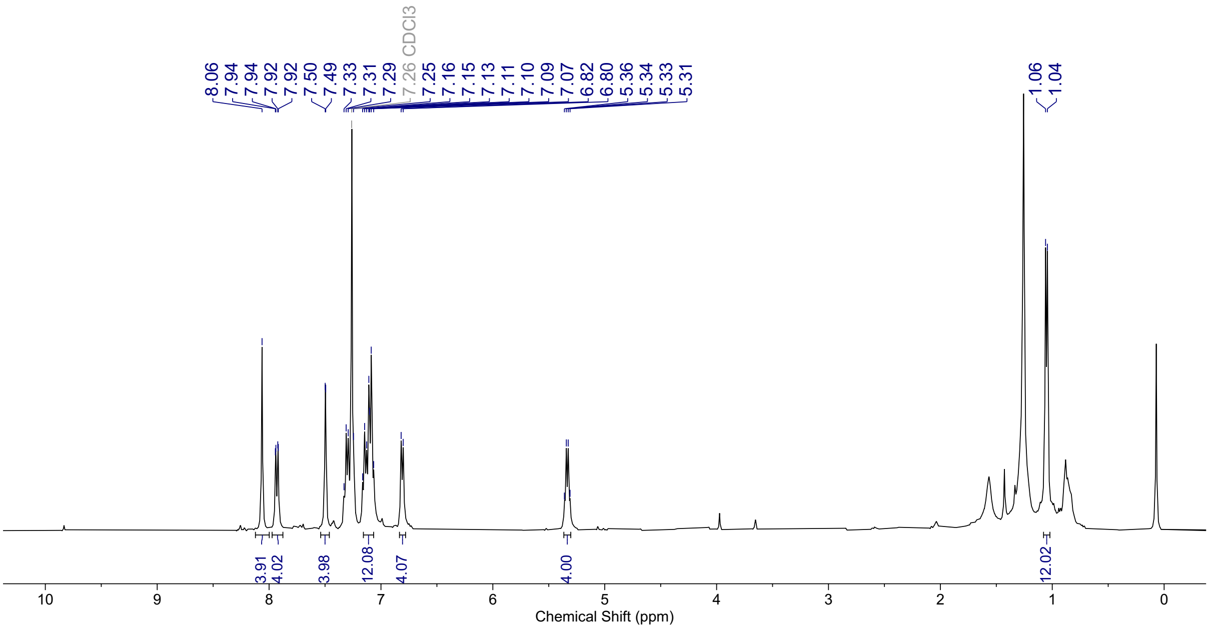

Supplementary Figure 28. ^{1}H NMR spectrum (400 MHz, 298 K) in $CDCl_3$ of (*P,P*)-8.

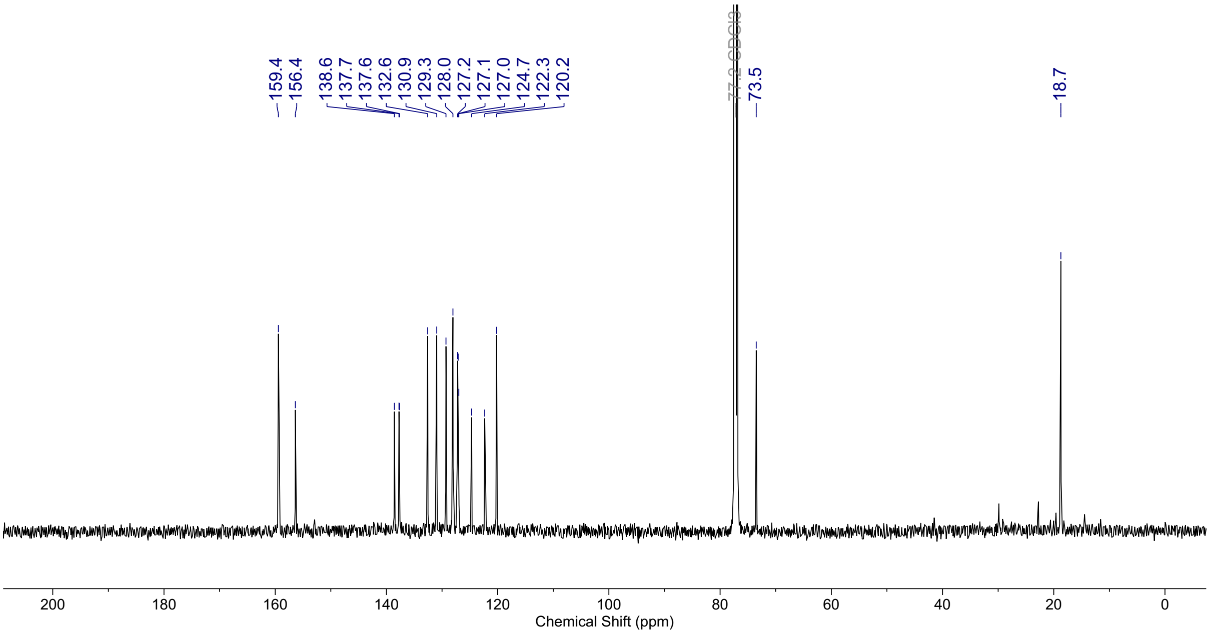

Supplementary Figure 29. ^{13}C NMR spectrum (126 MHz, 298 K) in $CDCl_3$ of (*P,P*)-8.

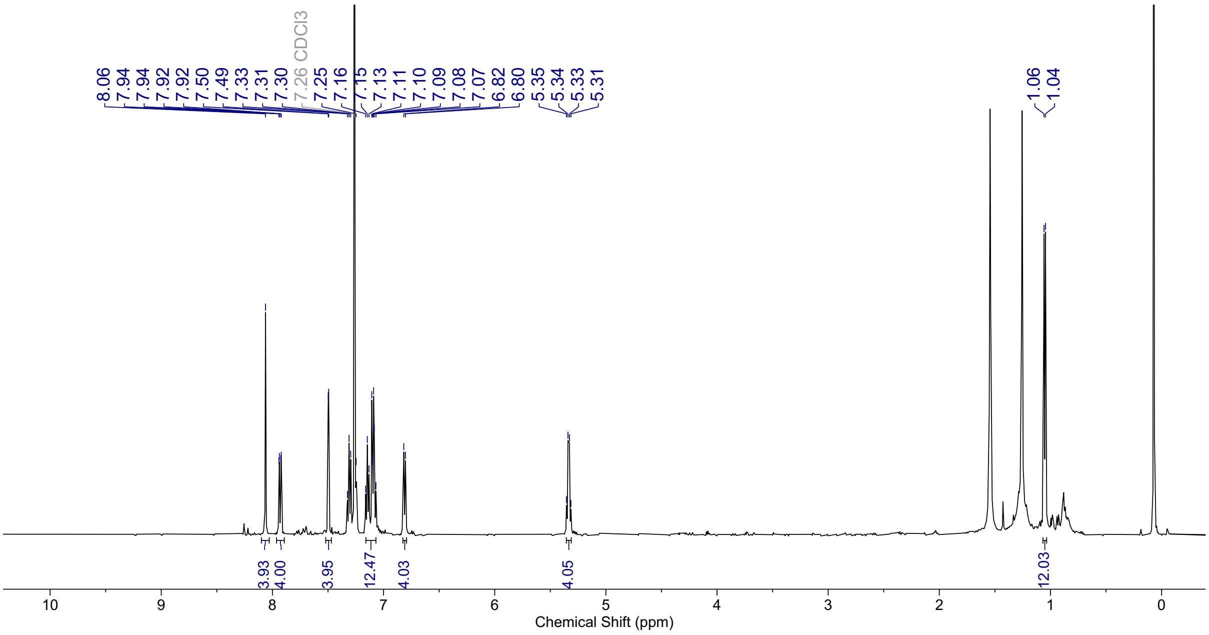

Supplementary Figure 30. ^{1}**H NMR spectrum (500 MHz, 298 K) in CDCl**$_3$ **of (*M,M*)-8.**

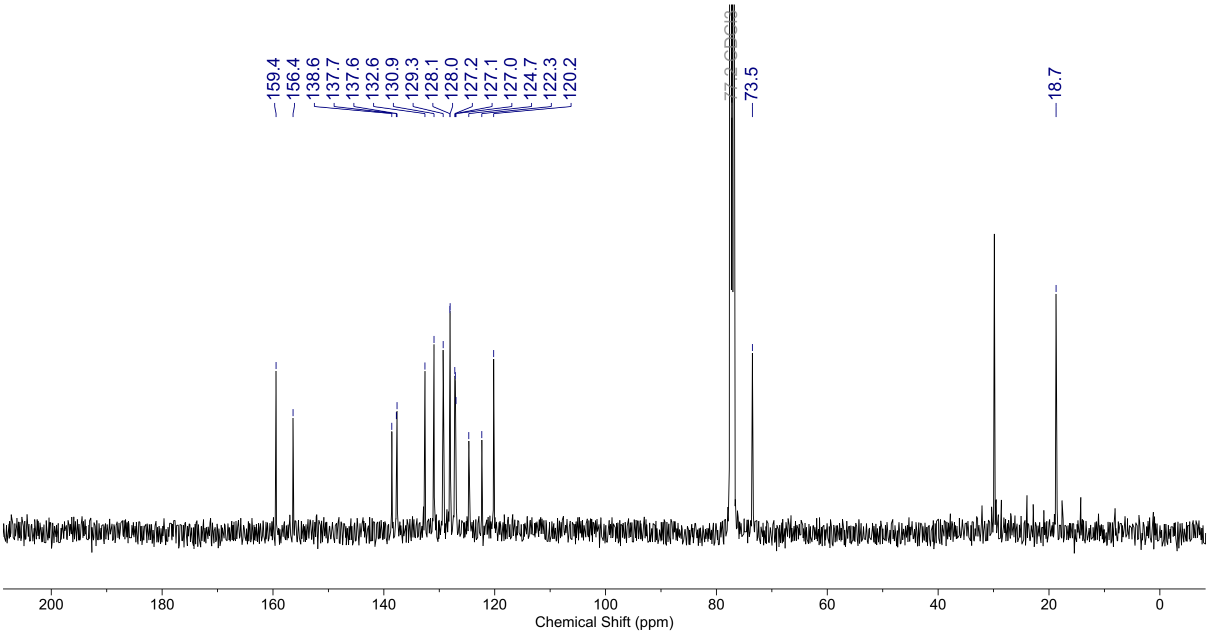

Supplementary Figure 31. ^{13}**C NMR spectrum (126 MHz, 298 K) in CDCl**$_3$ **of (*M,M*)-8.**

3. Supplementary Single-Crystal X-Ray Diffraction Data

Supplementary Table 1. Crystal data and structure refinement parameters for compound *P*(*S,S*)-6.

**CCDC Number**	2328087
**Empirical formula**	$C_{38}H_{30}O_6$
**Formula weight**	582.62
**Temperature / K**	100(2)
**Crystal system**	Orthorhombic
**Space group**	$P2_12_12_1$
***a* / Å**	8.9996(3)
***b* / Å**	17.9430(7)
***c* / Å**	18.7236(7)
***α* / °**	90
***β* / °**	90
***γ* / °**	90
**Volume / Å^3**	3023.48(19)
Z	4
**ρ_{calc} / g/cm^3**	1.280
**μ/mm^{-1}**	0.086
***F*(000)**	1224
**Radiation**	MoKα (λ = 0.71073)
**2Θ range for data collection/°**	6.912 to 50.698
**Index ranges**	-10 ≤ h ≤ 10, -21 ≤ k ≤ 21, -22 ≤ l ≤ 22
**Reflections collected**	22355
**Independent reflections**	5516 [R_{int} = 0.0720, R_{sigma} = 0.0708]
**Data/restraints/parameters**	5516/0/397
**Goodness-of-fit on *F*^{2}**	1.037
**Final *R* indexes [I>=2σ(I)]**	R_1 = 0.04769, wR_2 = 0.0992
**Final *R* indexes [all data]**	R_1 = 0.0691, wR_2 = 0.1095
**Largest diff. peak/hole / e Å^{-3}**	0.21/-0.22
**Flack parameter**	-0.4(8)

CheckCIF:

Alert level B

PLAT910_ALERT_3_B Missing # of FCF Reflection(s) Below Theta(Min).

110, 020, 120, 101, 011, 111, 021, 121, 002, 102, 012, 112, 0 2 2.

Response: The unit cell is large, causing reflections affected by the beamstop

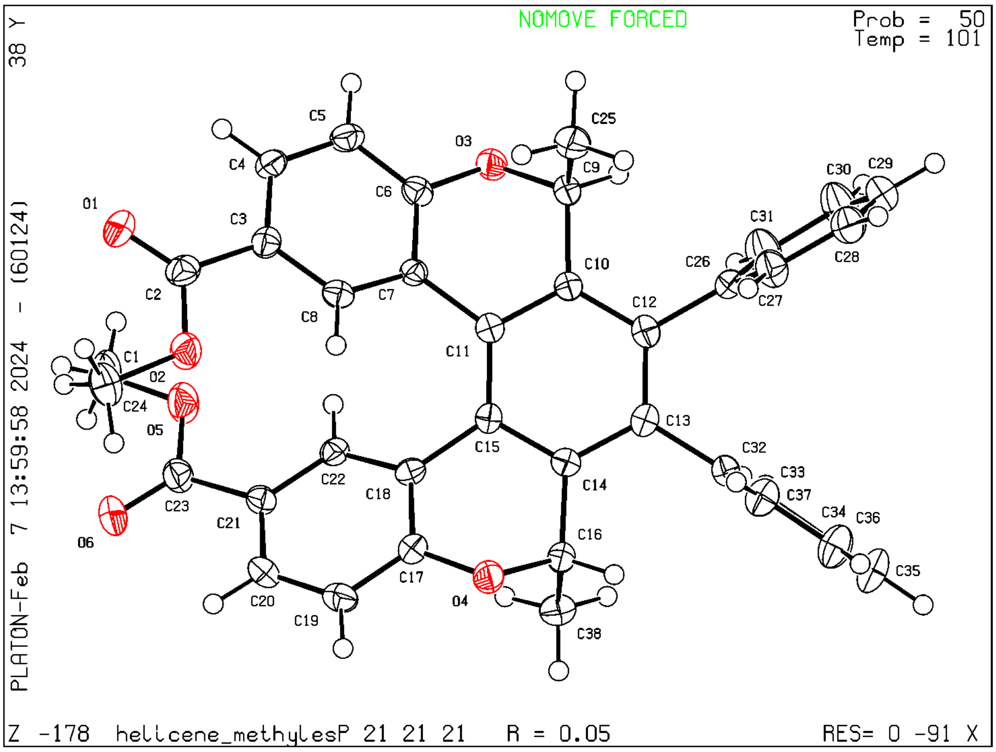

Supplementary Figure 32. ORTEP representation of [5]helicenoid *P*(*S,S*)-6. The thermal ellipsoids are drawn at 50% probability level.

4. Supplementary Computational Methods

Molecular modelling was carried out using Orca 5.0.2 or 5.0.3[2] or Gaussian 16 Rev A.03.[3] All structures were optimised using DFT with the X-ray data as the starting geometry. The DFT method used B3LYP or cam-B3LYP with Grimme's 3rd generation atom-pairwise dispersion correction with the Becke-Johnson damping scheme (D3BJ)[4] using def2-TZVP, def2-TZVP(-f) or def2-QZVP basis set[5] and def2/J auxiliary basis set.[6] The geometry optimisation and vibrational frequencies calculations were performed using either the SMD[7] solvent ($CHCl_3$) effect model or the CPCM model.[8] The sTDDFT[9] rotational barrier calculations were performed without a solvent model. A FAIR data archive for the calculations is available.[10]

The dissymmetry factor (g_{abs}) was calculated using SpecDis[11] 1.71 from the predicted UV & CD transitions using the sTDDFT method described above.

Supplementary Table 2. Calculated dissymmetry factors (g_{abs}) for compounds *P*(*S,S*)-6, *P*(*S,S*)-7 and (*M,M*)-8.

Compound	Wavelength (nm)	Dissymmetry factor, *g*
P(*S,S*)-**6**	248	-0.0018
P(*S,S*)-**7**	257	-0.0019
(*M,M*)-**8**	344	-0.0071

5. Supplementary Data: Conformational Stability of Helicenoids 6 and 7

The conformational stability of [5]helicenoids **6** and **7** was investigated both experimentally and computationally. Given that the helicenoid structure contains two stereogenic elements (point and helical chirality), inversion of the helicity results in the formation of the diastereomer and not the enantiomer *i.e.* *P*(*S,S*)-**6** inverts to *M*(*S,S*)-**6** and not *M*(*R,R*)-**6**. As such, this is formally an epimerisation process.

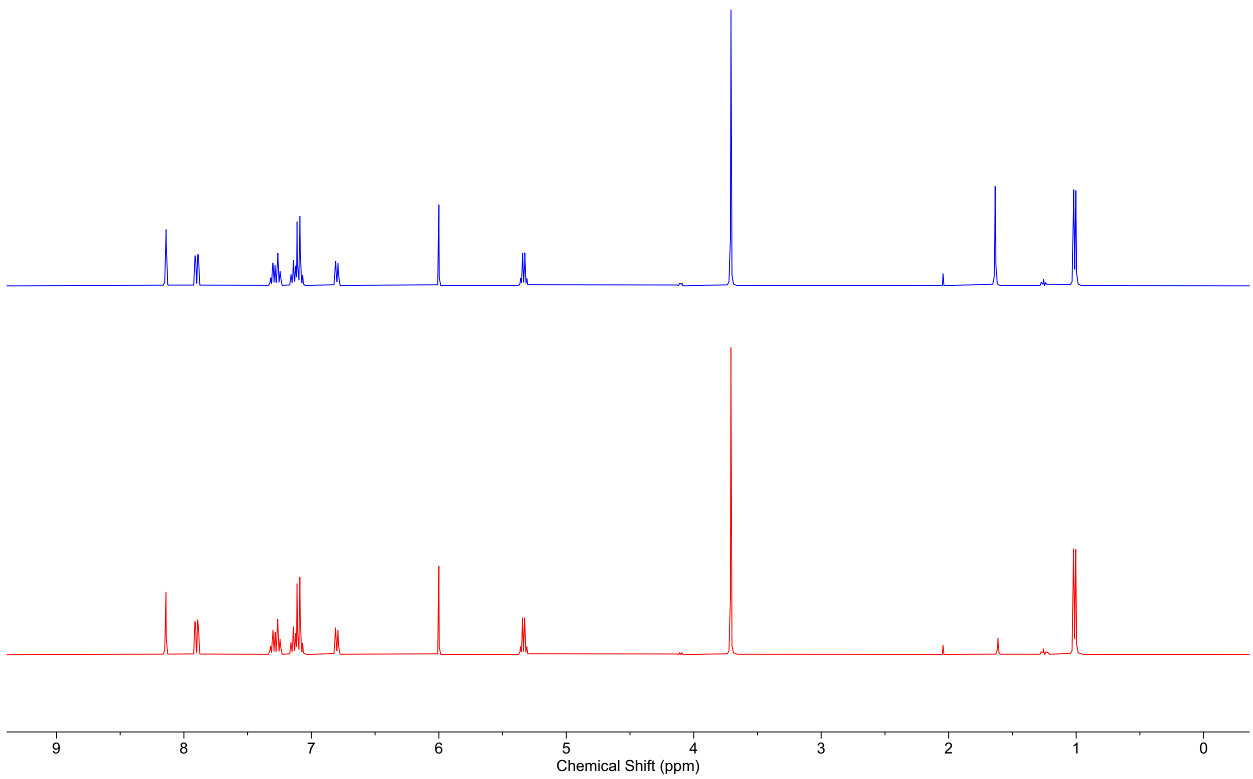

Supplementary Figure 33. **^{1}H NMR spectra (400 MHz, 298 K) in TCE-*d*$_2$ of *P*(*S,S*)-6 acquired pre (blue) and post (red) heating at 140 °C for 24 h. Spectra are unchanged indicating no epimerisation has occurred.**

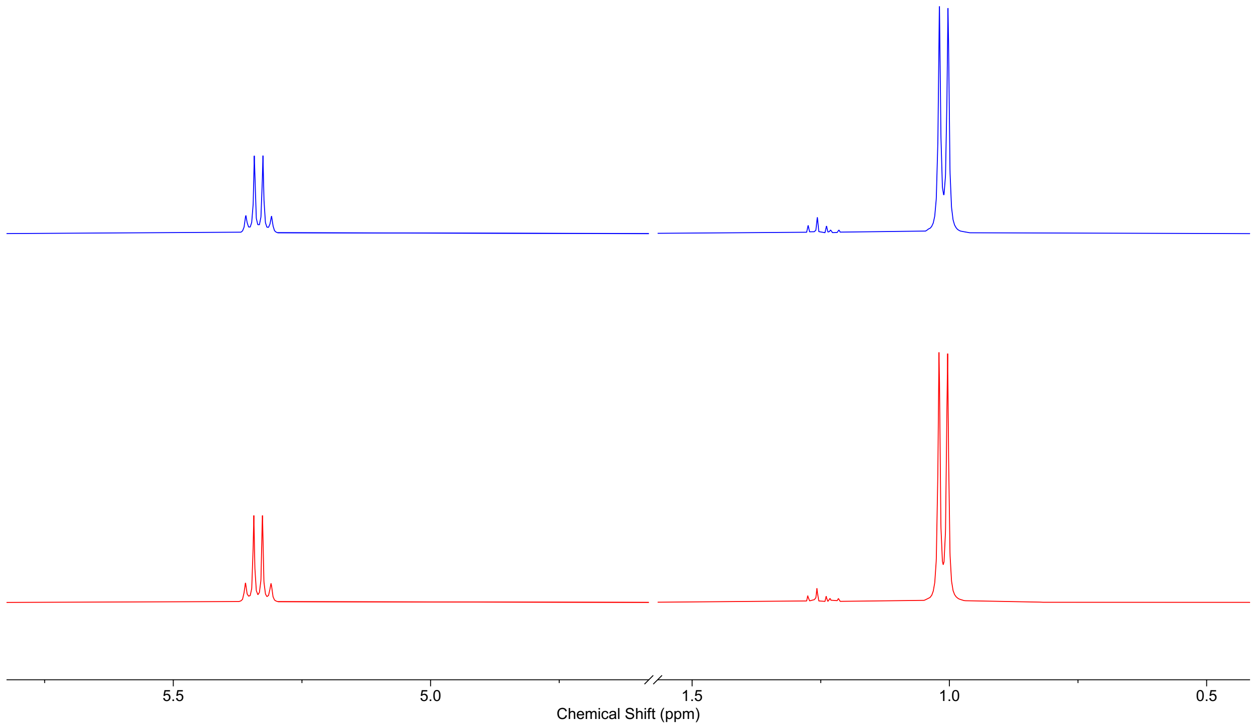

Supplementary Figure 34. Zoomed ^{1}H NMR spectra (400 MHz, 298 K) in TCE-*d*$_2$ of *P*(*S,S*)-6 acquired pre (blue) and post (red) heating at 140 °C for 24 h showing protons H6 (5.34 ppm) and H12 (1.01 ppm). Spectra are unchanged indicating no epimerisation has occurred.

To further investigate the conformational stability of helicenoids **6** and **7**, the epimerisation process was investigated computationally. It is understood that enantiomers of [*n*]helicenes interconvert *via* a conformational pathway, having been demonstrated for other helicene-based structures[12] and is therefore applied to the epimerisation of helicenoids **6** and **7**. The inversion of helicity is modelled by systematically varying the dihedral angle of the inner helix, highlighted in Figures S35 and S36. It is assumed that the highest energy geometry is representative of the transition state and therefore the barrier to epimerisation is calculated as the energy difference between the starting and highest energy geometries. Energy minimisation (DFT, B3LYP/D3BJ/def2TZVP) was performed for each geometry.

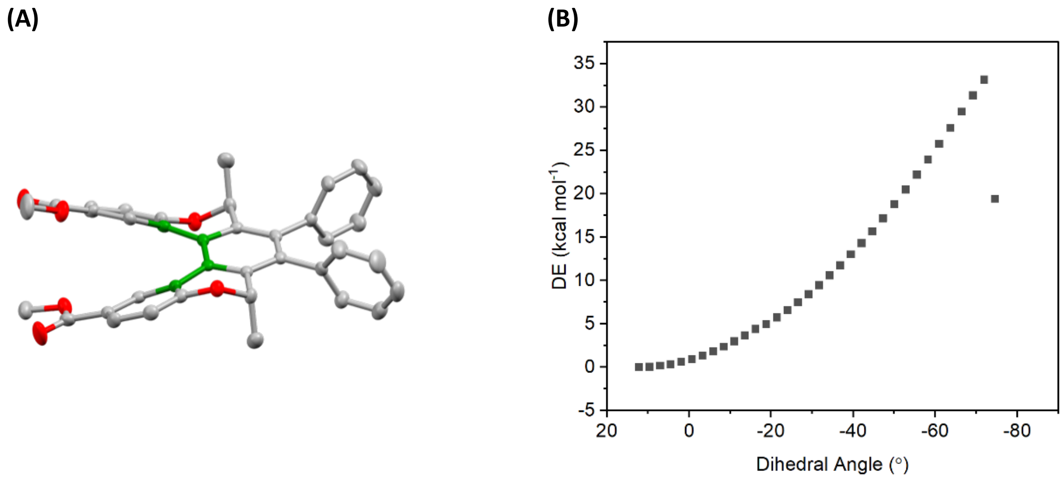

Supplementary Figure 35. A) X-ray structure of helicenoid *P*(*S,S*)-6 with highlighted dihedral angle; B) energy profile of the *P*(*S,S*)-6 to *M*(*S,S*)-6 epimerisation. Energy barrier was calculated as 33.2 kcal mol^{-1}.

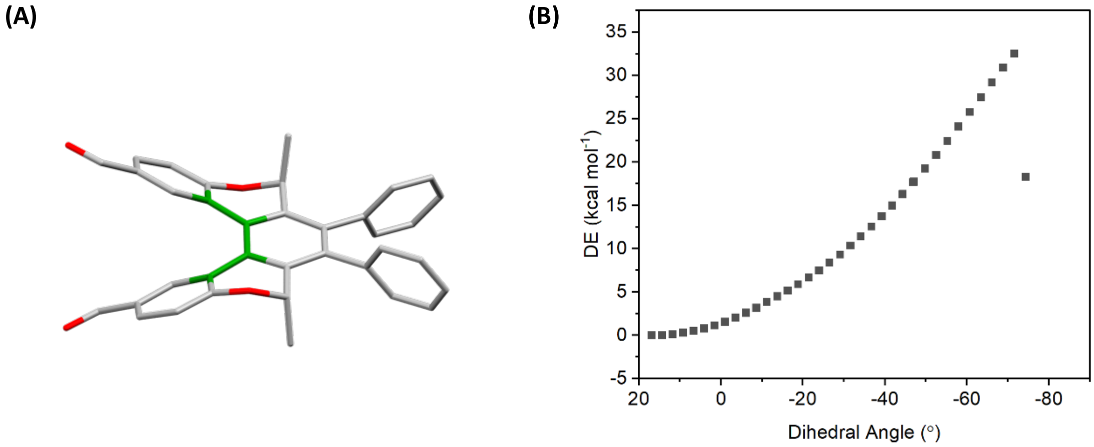

Supplementary Figure 36. A) Modelled structure of helicenoid *P*(*S,S*)-7 with highlighted dihedral angle; B) energy profile of the *P*(*S,S*)-7 to *M*(*S,S*)-7 epimerisation. Energy barrier calculated as 32.5 kcal mol^{-1}.

6. Supplementary Data: Self-Sorting Studies

DCL Preparation

Stock solutions of *P*(*S,S*)-**7** (5 mM) and *M*(*R,R*)-**7** (5 mM) in THF (10 mL) were prepared. Aliquots (0.5 mL) of these solutions were combined to prepare a racemic solution of compound **7** (5 mM), to which hydrazine monohydrate (2.4 µL, 0.050 mmol, 1.0 equiv) was added. The library was stirred in an air-capped vial and analysed over the duration of 1 week *via* HPLC, after which time complete self-sorting was observed.

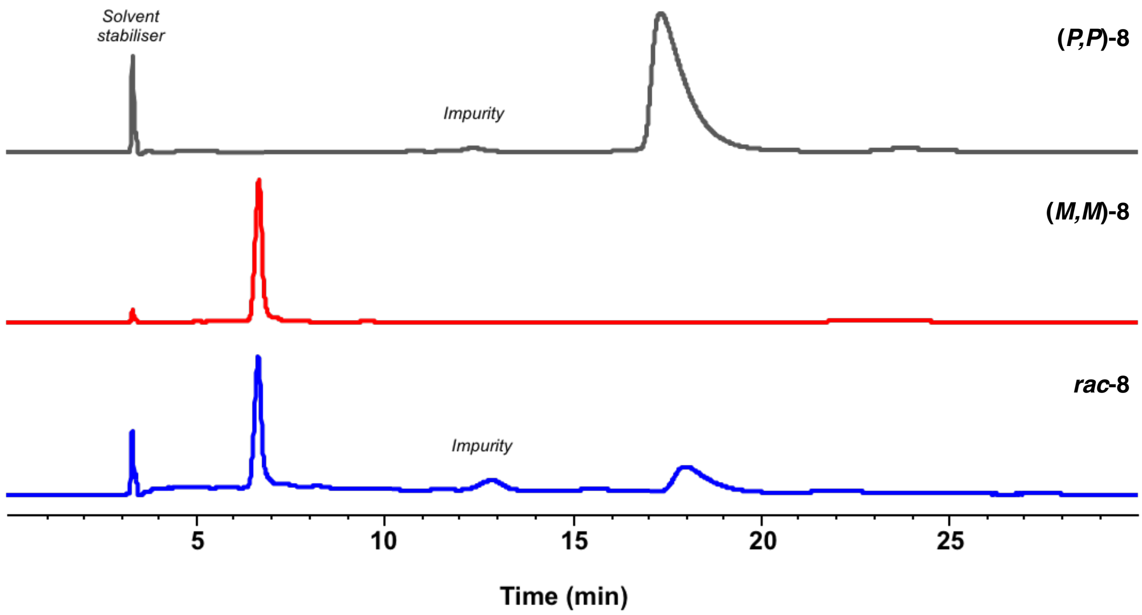

Supplementary Figure 37. HPLC chromatograms of enantiopure samples of (*P,P*)-8 (black) and (*M,M*)-8 (red) compared against the DCL containing *rac*-7 and hydrazine (*rac*-8, blue). Absorbance is recorded at 280 nm. Performed on a Daicel CHIRALPAK IB 5 µm, 4.6 x 250 mm column using 100% acetonitrile mobile phase.

The observed preference for homochiral over heterochiral self-assembly prompted further computational analysis. In agreement with the experimental evidence, comparison of the calculated energies for the energy minimised models of (*M,M*)-**8** and (*M,P*)-**8** reveals that (*M,M*)-**8** is more stable than the heterochiral system (*M,P*)-**8** by 6.20 kcal mol^{-1}.

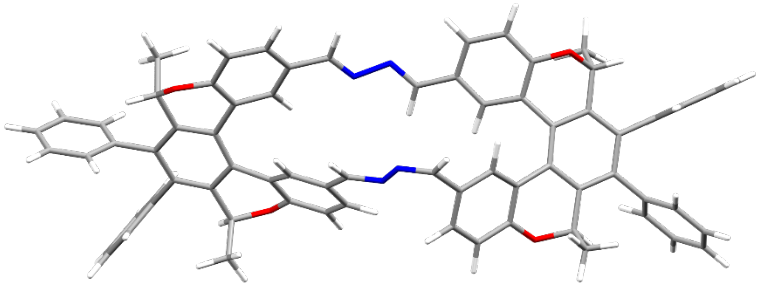

Supplementary Figure 38. Energy minimised model of the heterochiral macrocyclic dimer (*M,P*)-8.

7. Supplementary MMP spectra and maps

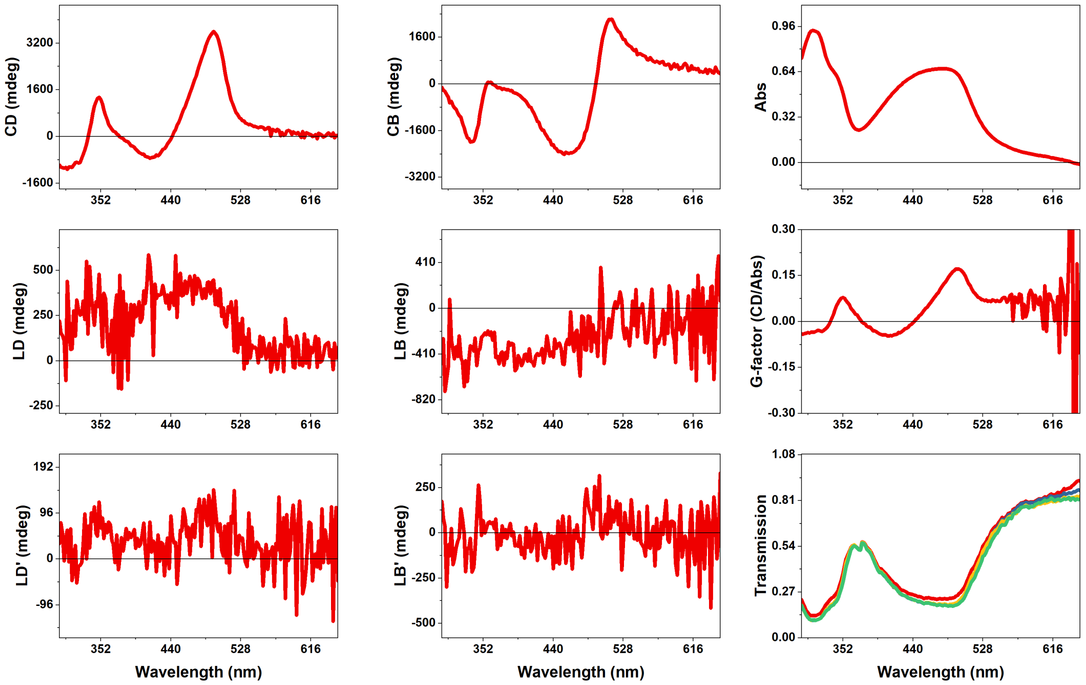

Supplementary Figure 39. CD, CB, absorption, LD, LB, g-factor, LD', LB' and transmission spectra of F8BT thin film with 10% *P*(*S*,*S*)-7.

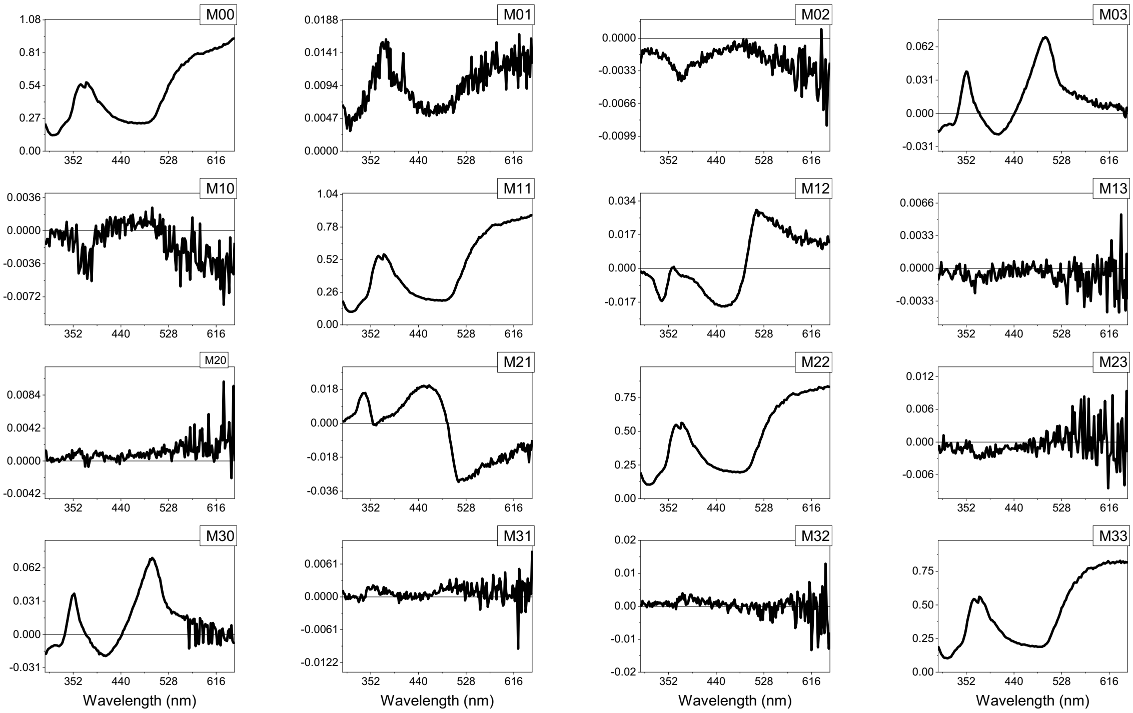

Supplementary Figure 40. Raw MMP elements spectra of F8BT thin film with 10% *P*(*S*,*S*)-7.

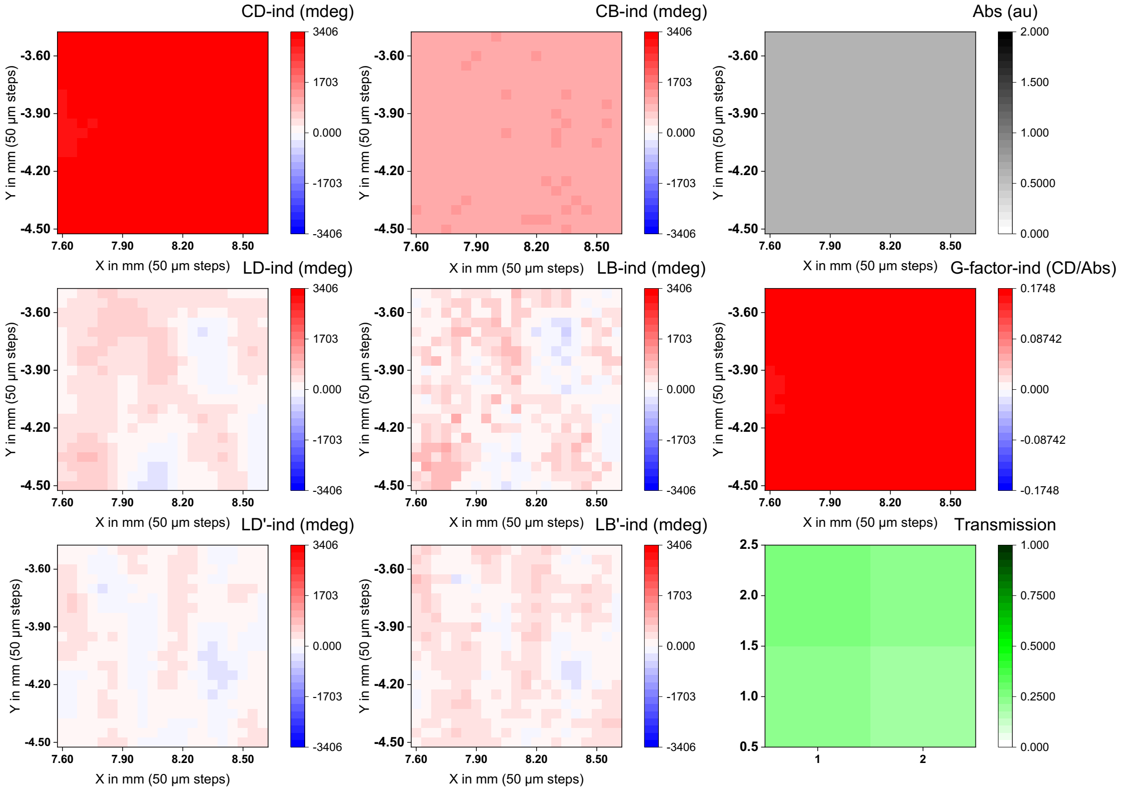

**Supplementary Figure 41. CD, CB, absorption, LD, LB, g-factor, LD', LB' and transmission maps at 500 nm (1x1 mm,
50 *μ*m spatial resolution) of F8BT thin film with 10% *P*(*S*,*S*)-7.**

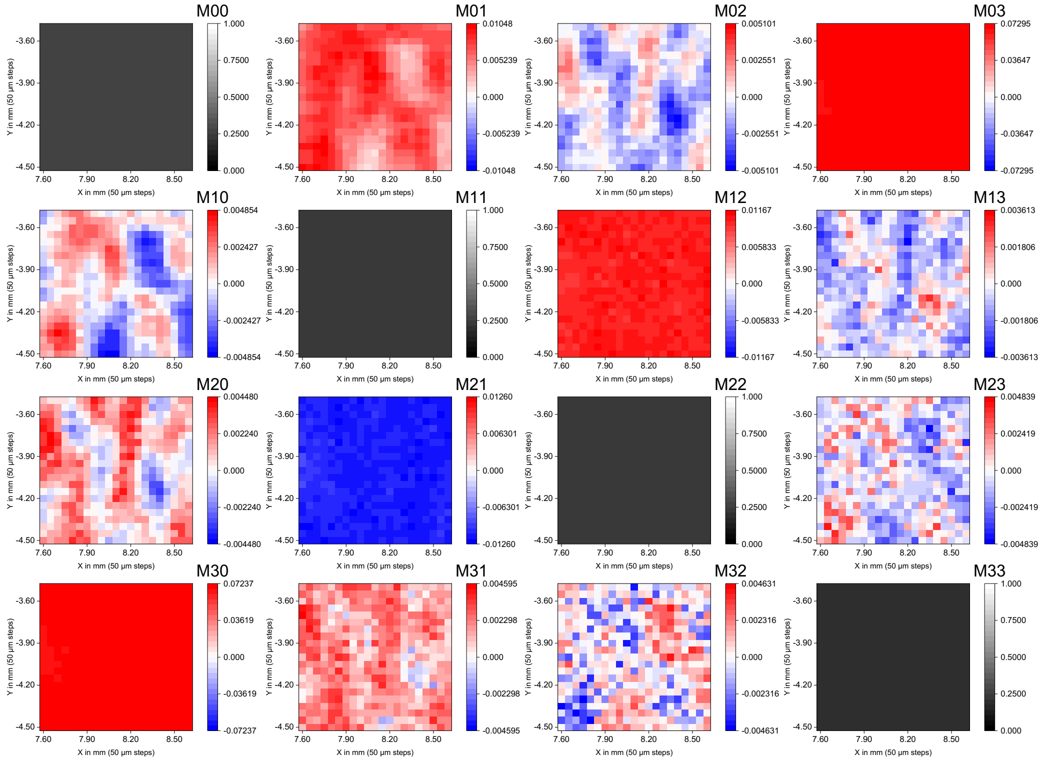

Supplementary Figure 42. Raw MMP elements maps at 500 nm (1x1 mm, 50 *μ*m spatial resolution) of F8BT thin film with 10% *P*(*S*,*S*)-7.

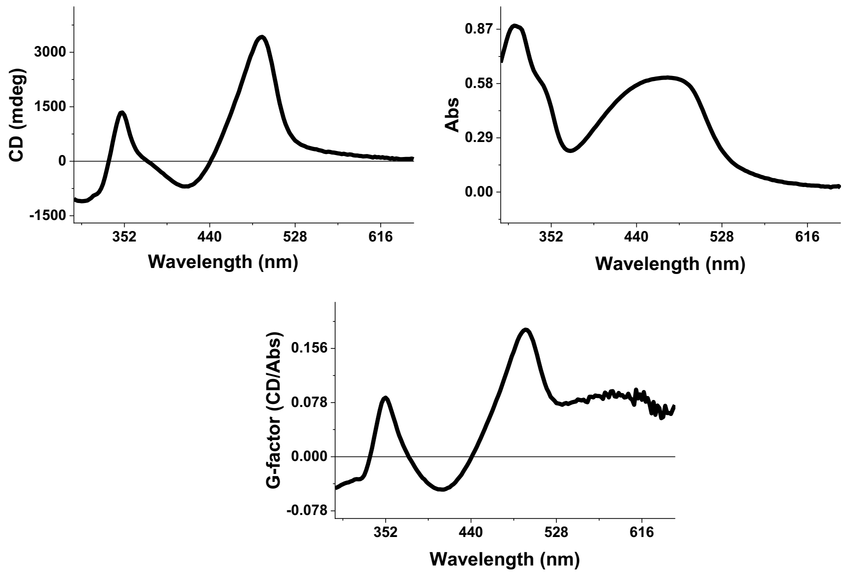

Supplementary Figure 43. CD, absorption and g-factor spectra of F8BT thin film with 10% *P*(*S*,*S*)-7 recorded using the CD-mode of the MMP instrument.

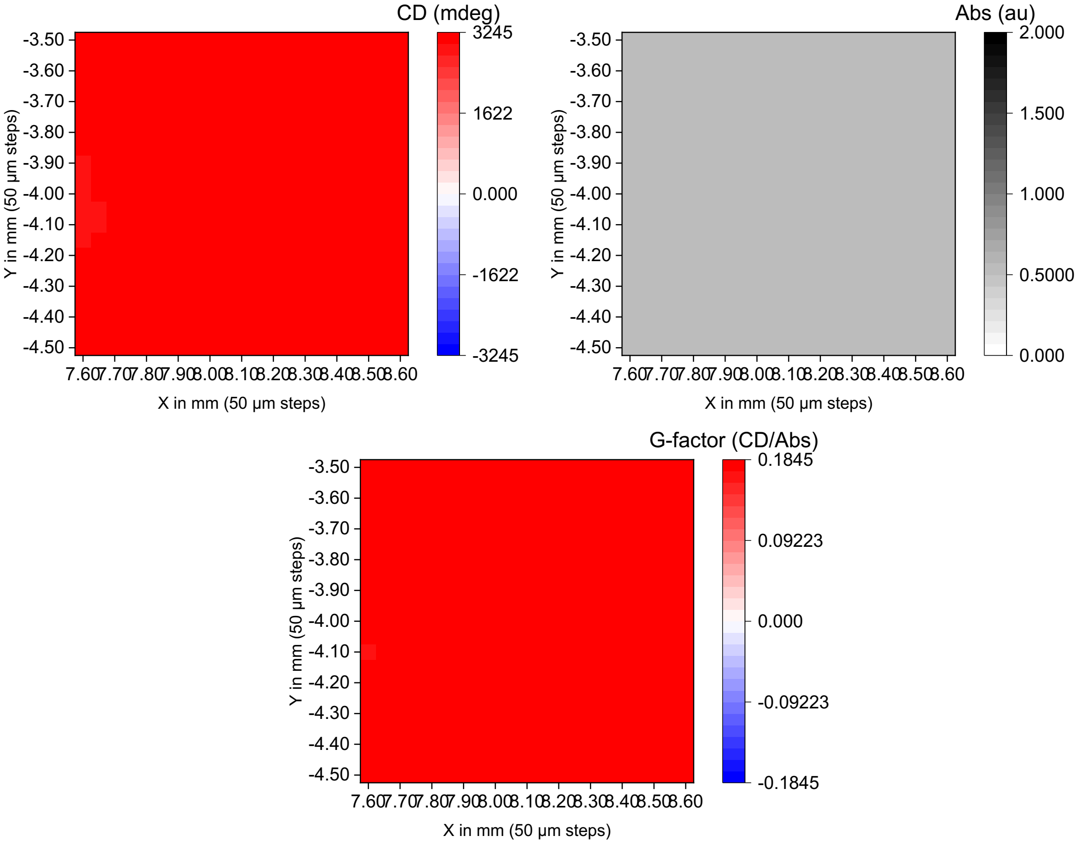

Supplementary Figure 44. CD, absorption and g-factor maps at 494 nm of F8BT thin film with 10% *P*(*S*,*S*)-7 recorded using the CD-mode of the MMP instrument.

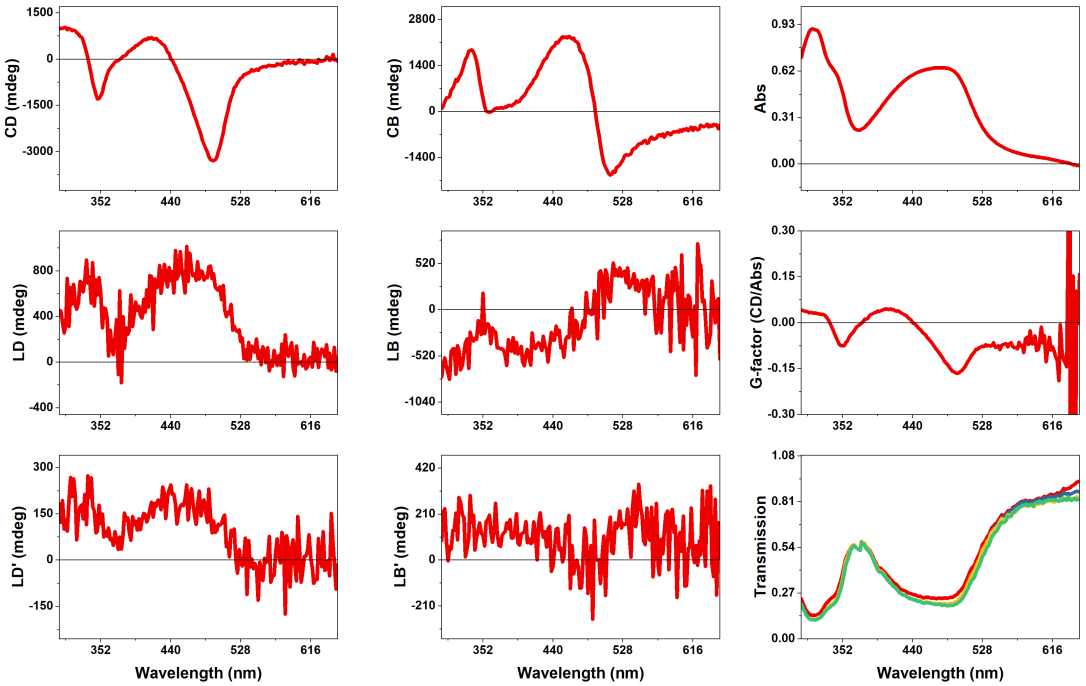

Supplementary Figure 45. CD, CB, absorption, LD, LB, g-factor, LD', LB' and transmission spectra of F8BT thin film with 10% *M*(*R*,*R*)-7.

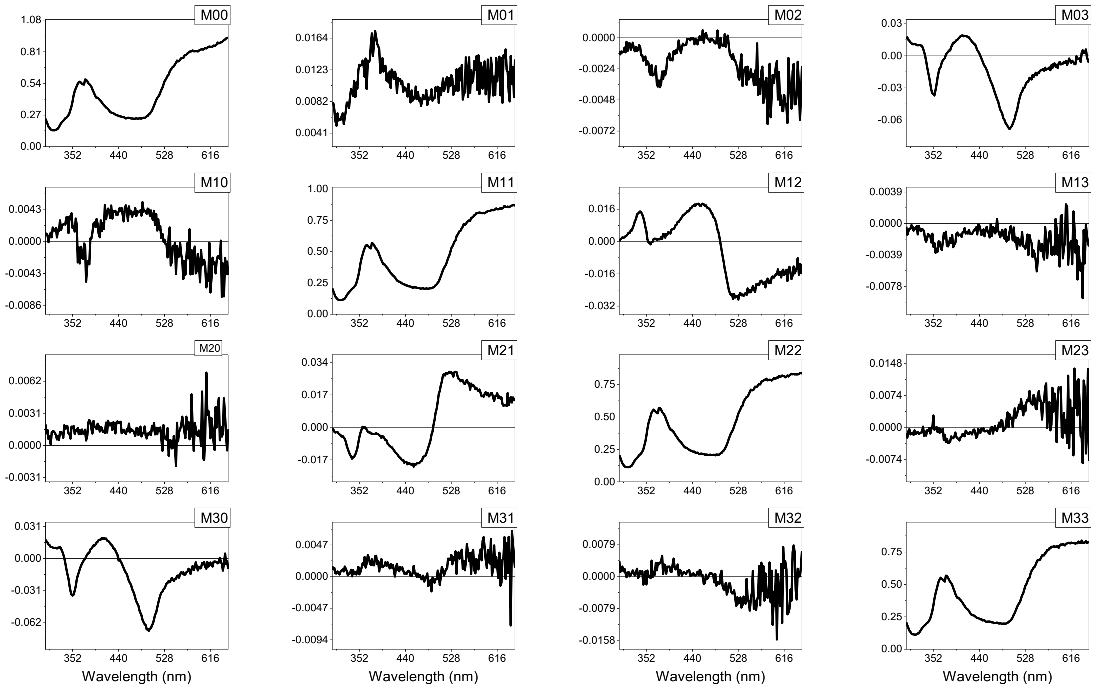

Supplementary Figure 46. Raw MMP elements spectra of F8BT thin film with 10% *M*(*R*,*R*)-7.

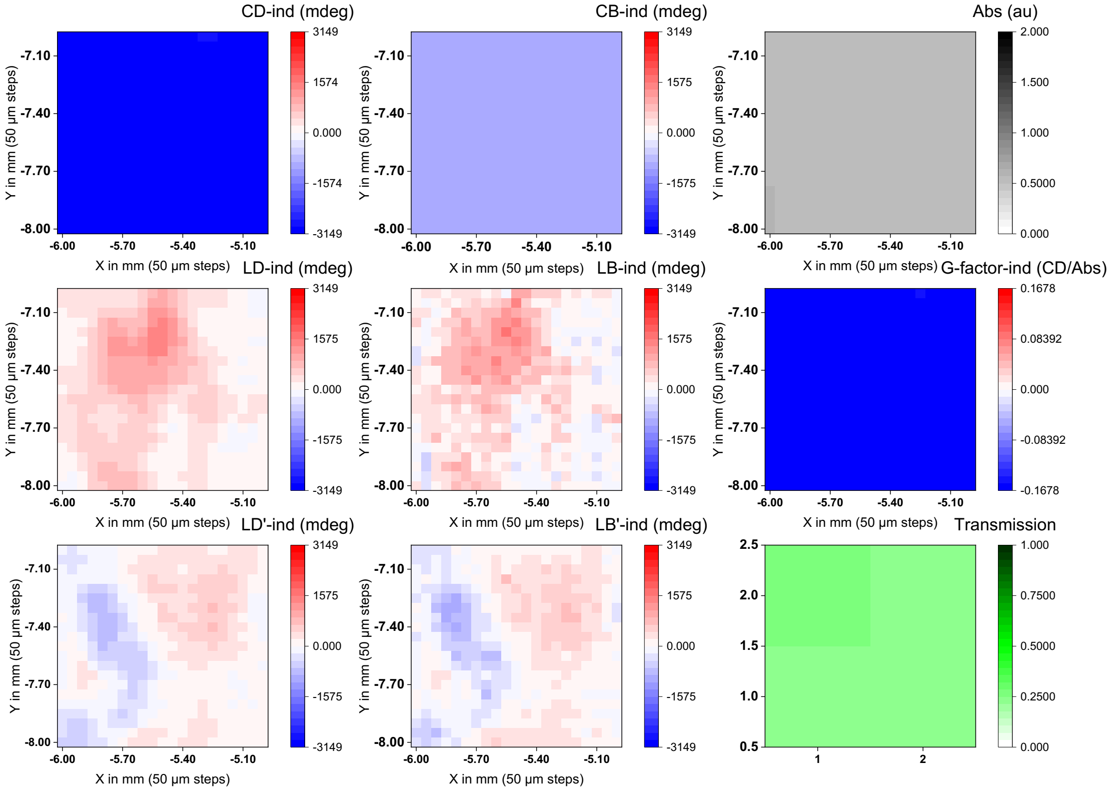

Supplementary Figure 47. CD, CB, absorption, LD, LB, g-factor, LD', LB' and transmission maps at 500 nm (1x1 mm, 50 μm spatial resolution) of F8BT thin film with 10% *M*(*R*,*R*)-7.

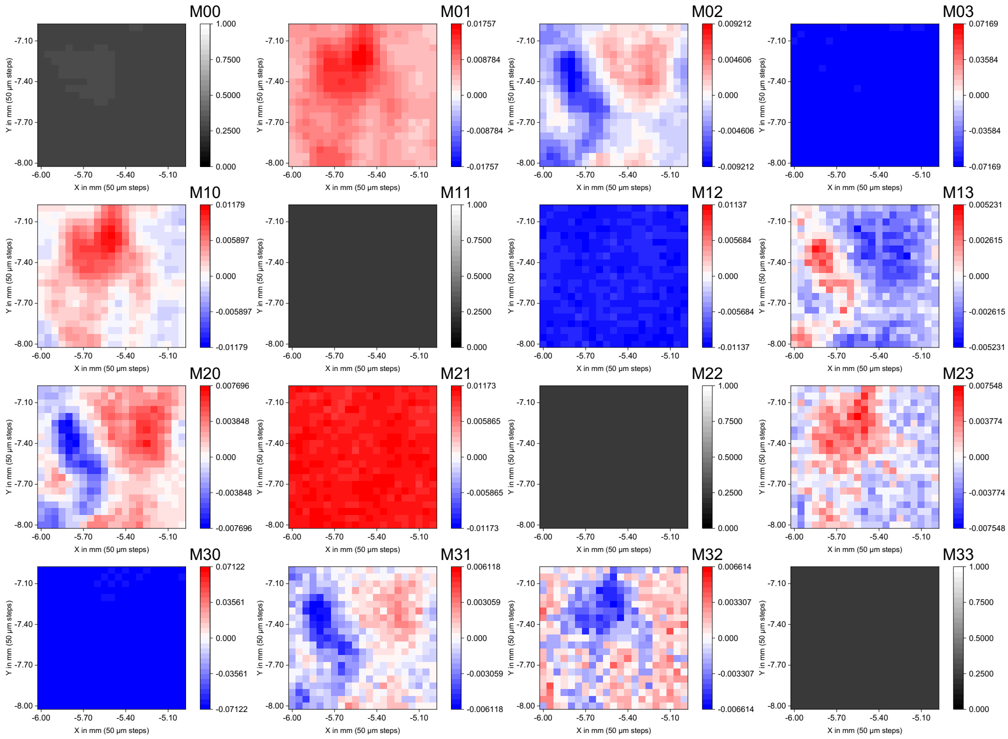

Supplementary Figure 48. Raw MMP elements maps at 500 nm (1x1 mm, 50 μm spatial resolution) of F8BT thin film with 10% *M*(*R*,*R*)-7.

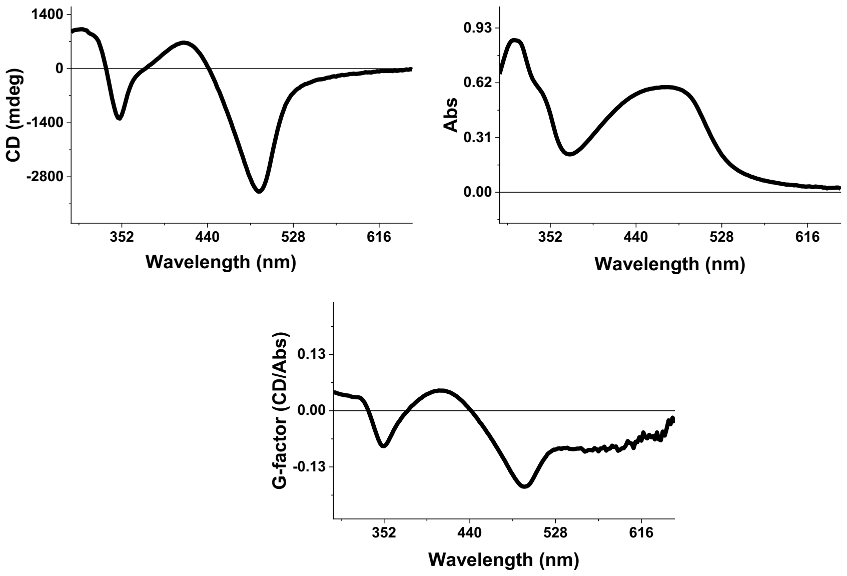

Supplementary Figure 49. CD, absorption and g-factor spectra of F8BT thin film with 10% *M*(*R*,*R*)-7 recorded using the CD-mode of the MMP instrument.

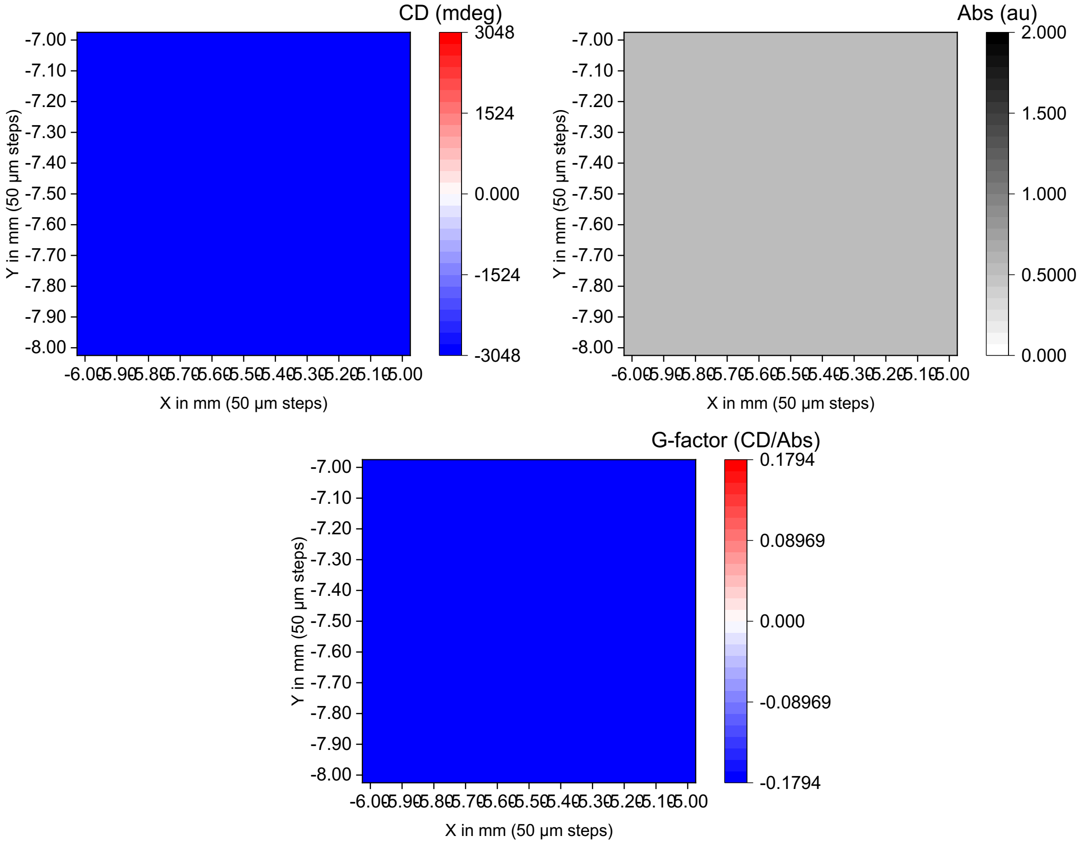

Supplementary Figure 50. CD, absorption and g-factor maps at 500 nm of F8BT thin film with 10% *M*(*R*,*R*)-7 recorded using the CD-mode of the MMP instrument.

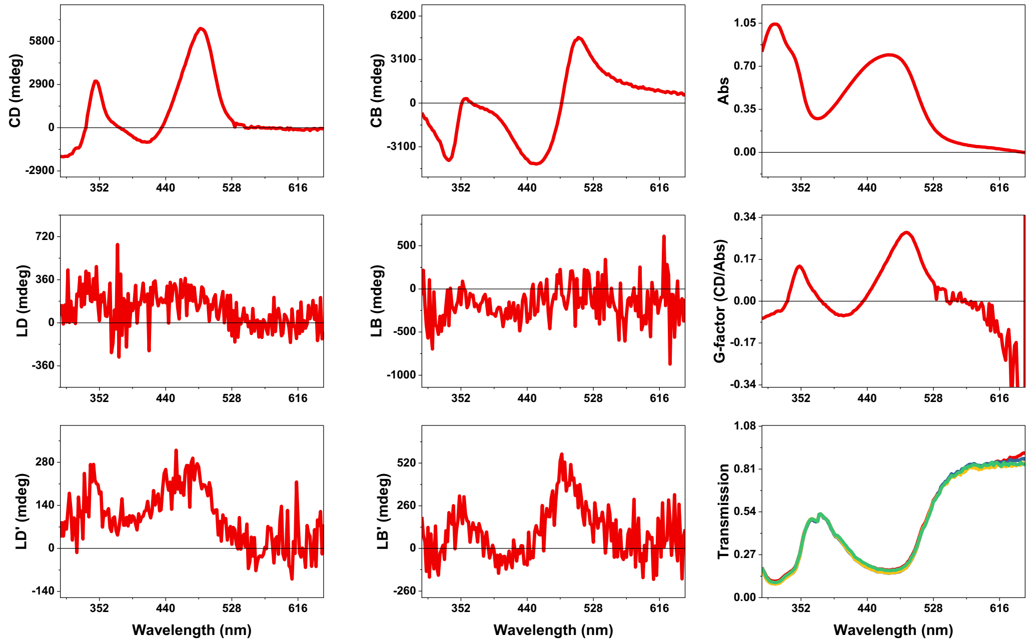

Supplementary Figure 51. CD, CB, absorption, LD, LB, g-factor, LD', LB' and transmission spectra of F8BT thin film with 10% (*P*,*P*)-8.

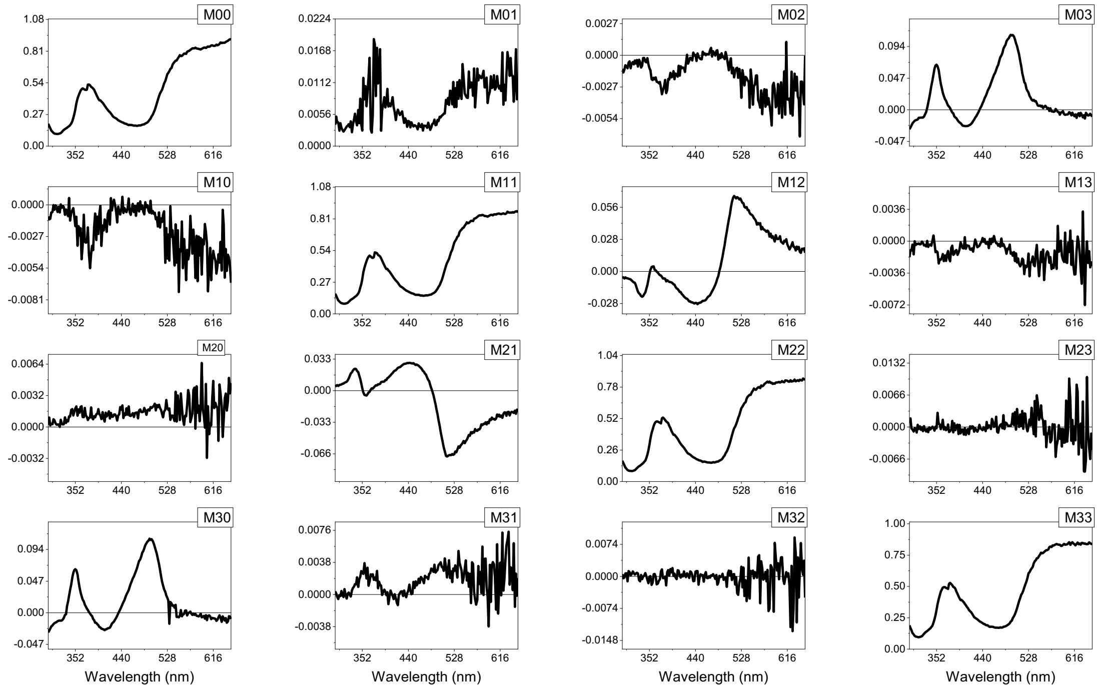

Supplementary Figure 52. Raw MMP elements spectra of F8BT thin film with 10% (*P*,*P*)-8.

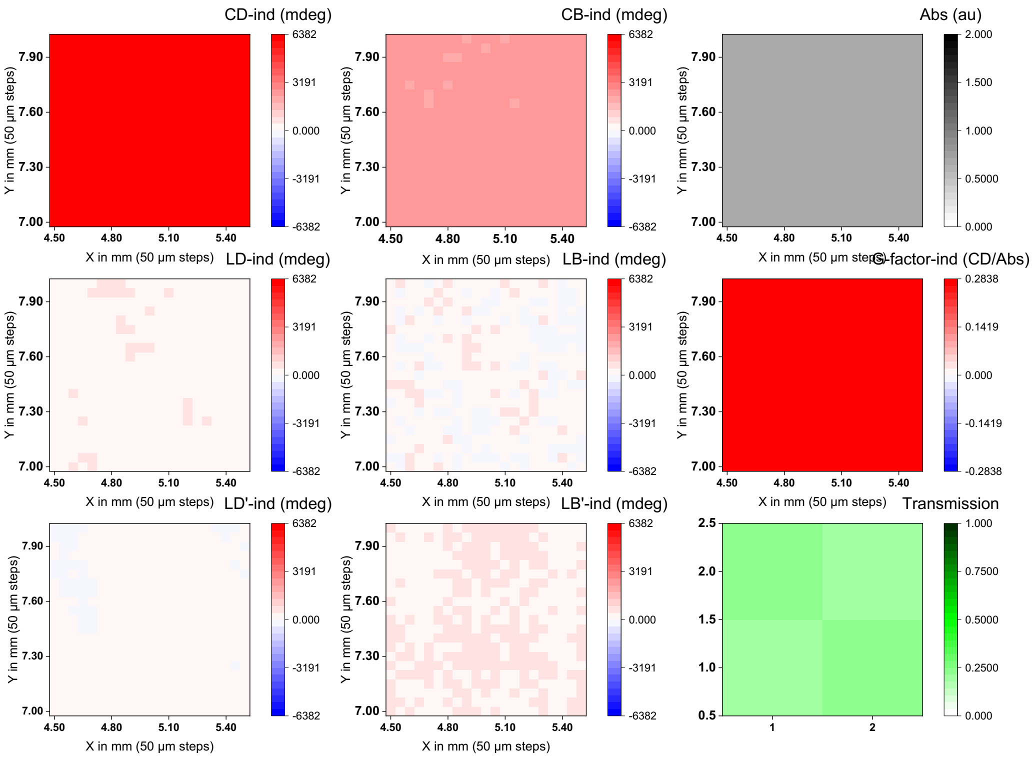

Supplementary Figure 53. CD, CB, absorption, LD, LB, g-factor, LD', LB' and transmission maps at 494 nm (1x1 mm,
50 *µ*m spatial resolution) of F8BT thin film with 10% (*P*,*P*)-8.

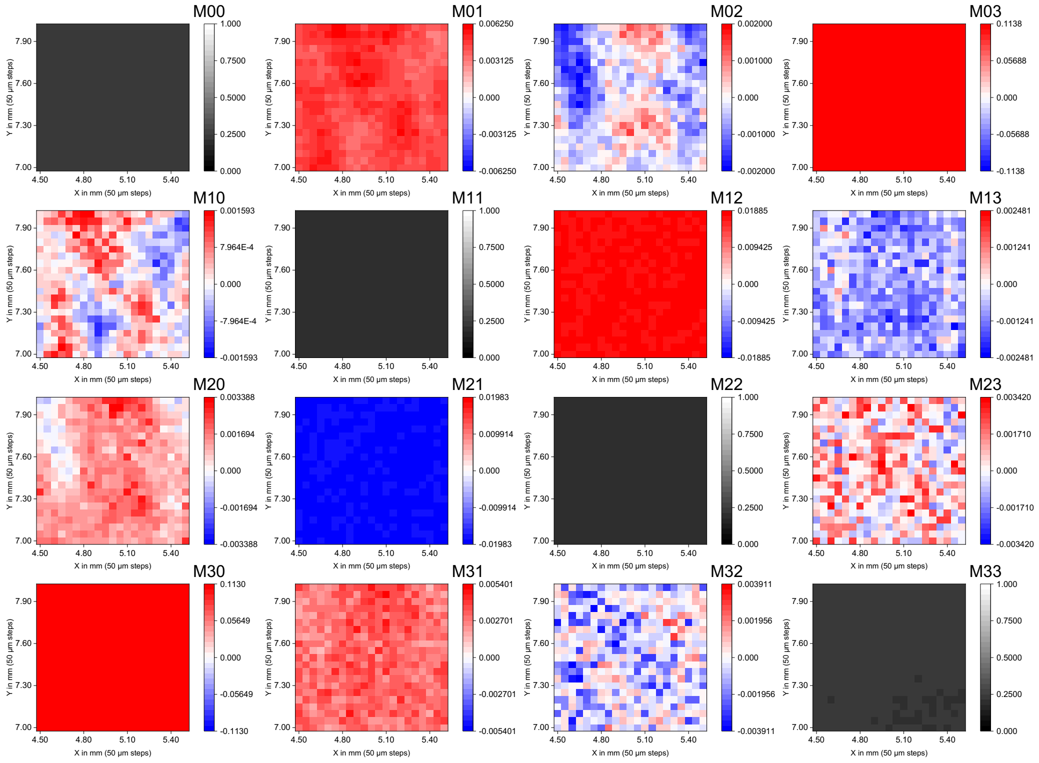

Supplementary Figure 54. Raw MMP elements maps at 494 nm (1x1 mm, 50 *µ*m spatial resolution) of F8BT thin film with 10% (*P*,*P*)-8.

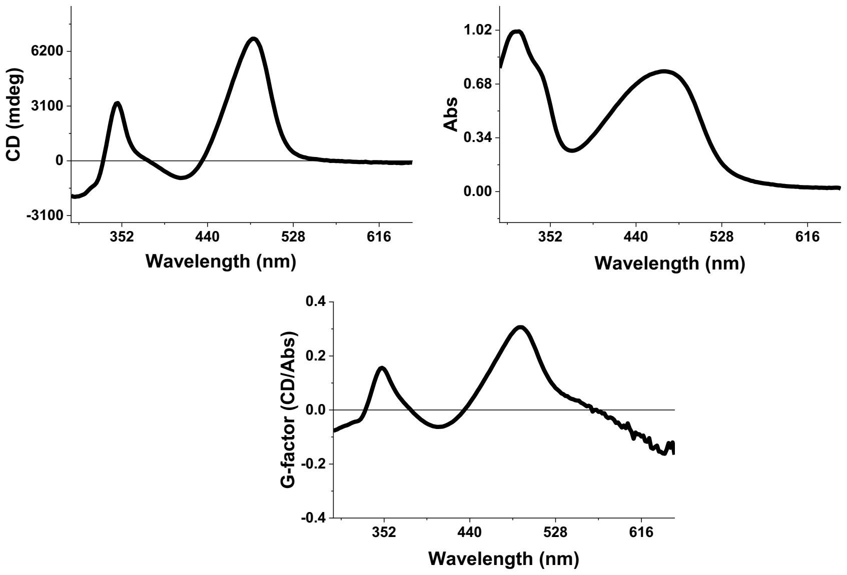

Supplementary Figure 55. CD, absorption and g-factor spectra of F8BT thin film with 10% (*P*,*P*)-8 recorded using the CD-mode of the MMP instrument.

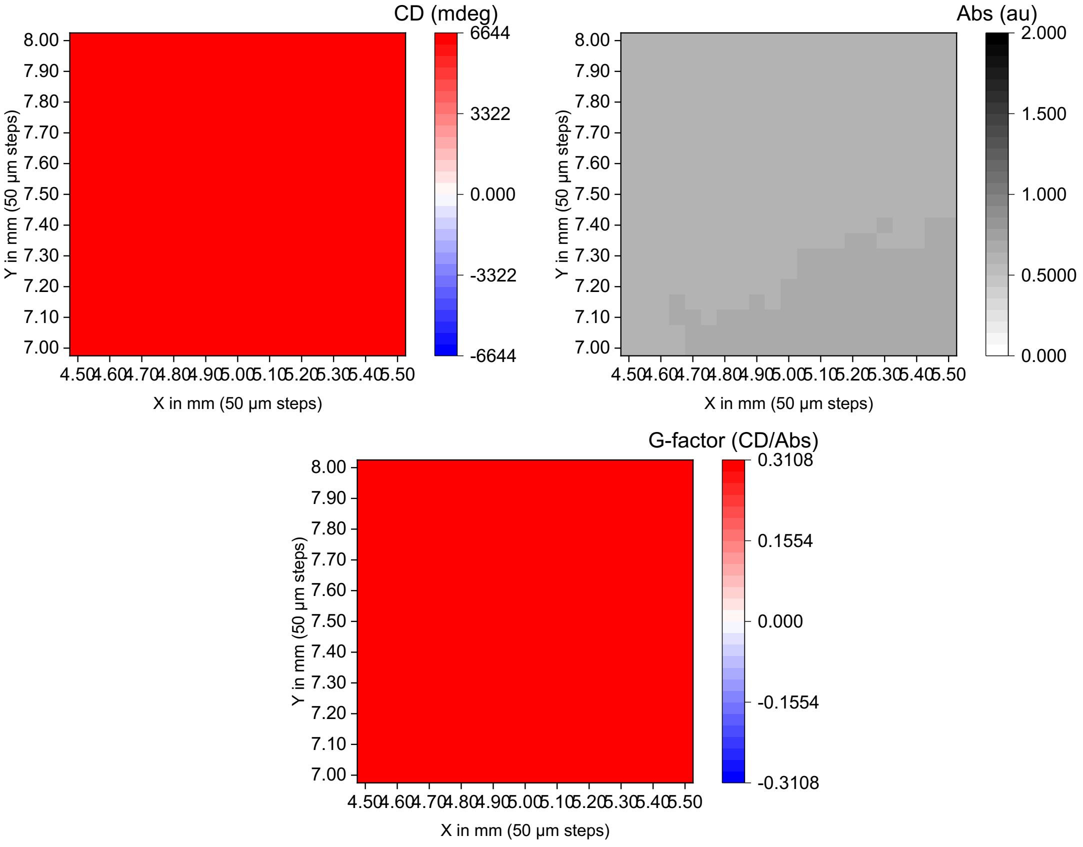

Supplementary Figure 56. CD, absorption and g-factor maps at 494 nm of F8BT thin film with 10% (*P*,*P*)-8 recorded using the CD-mode of the MMP instrument.

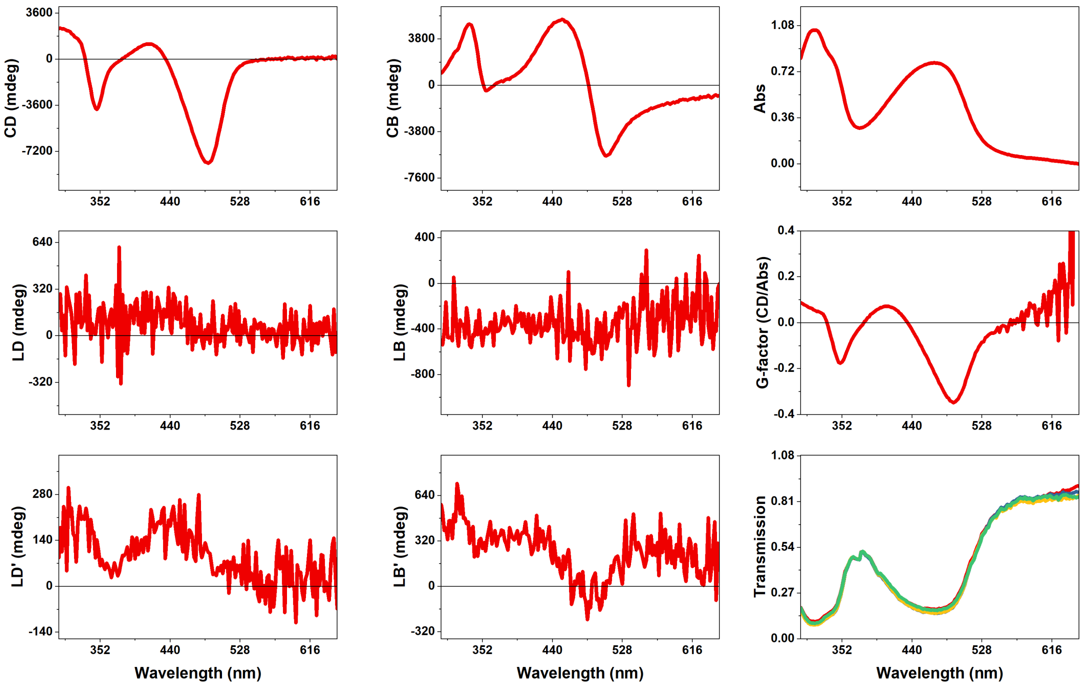

Supplementary Figure 57. CD, CB, absorption, LD, LB, g-factor, LD', LB' and transmission spectra of F8BT thin film with 10% (*M*,*M*)-8.

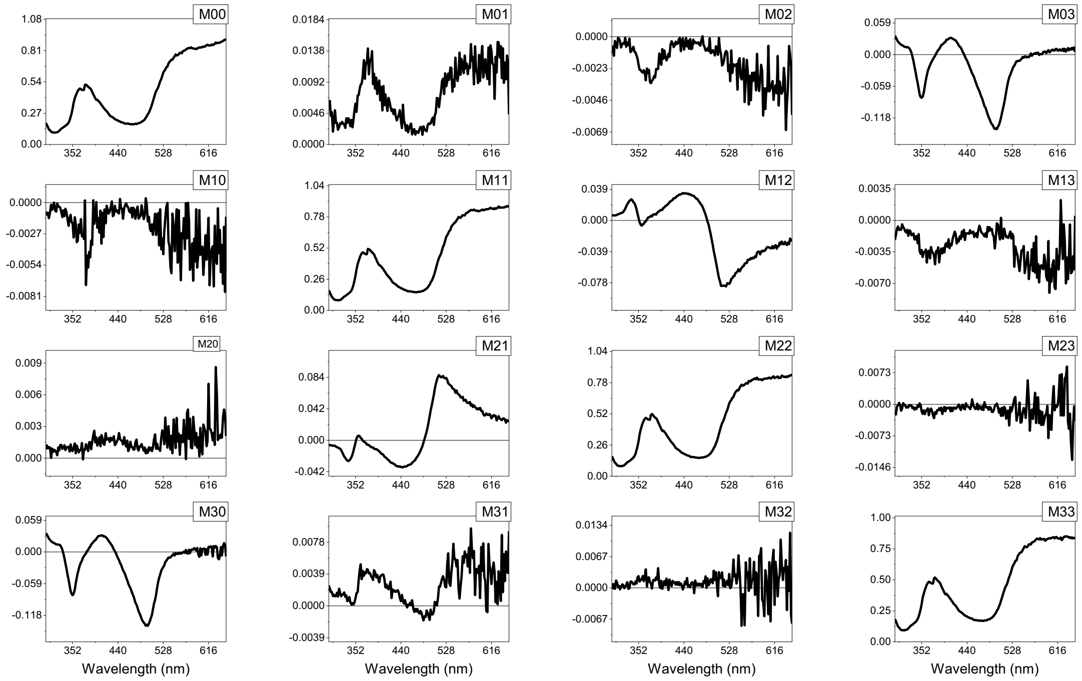

Supplementary Figure 58. Raw MMP elements spectra of F8BT thin film with 10% (*M*,*M*)-8.

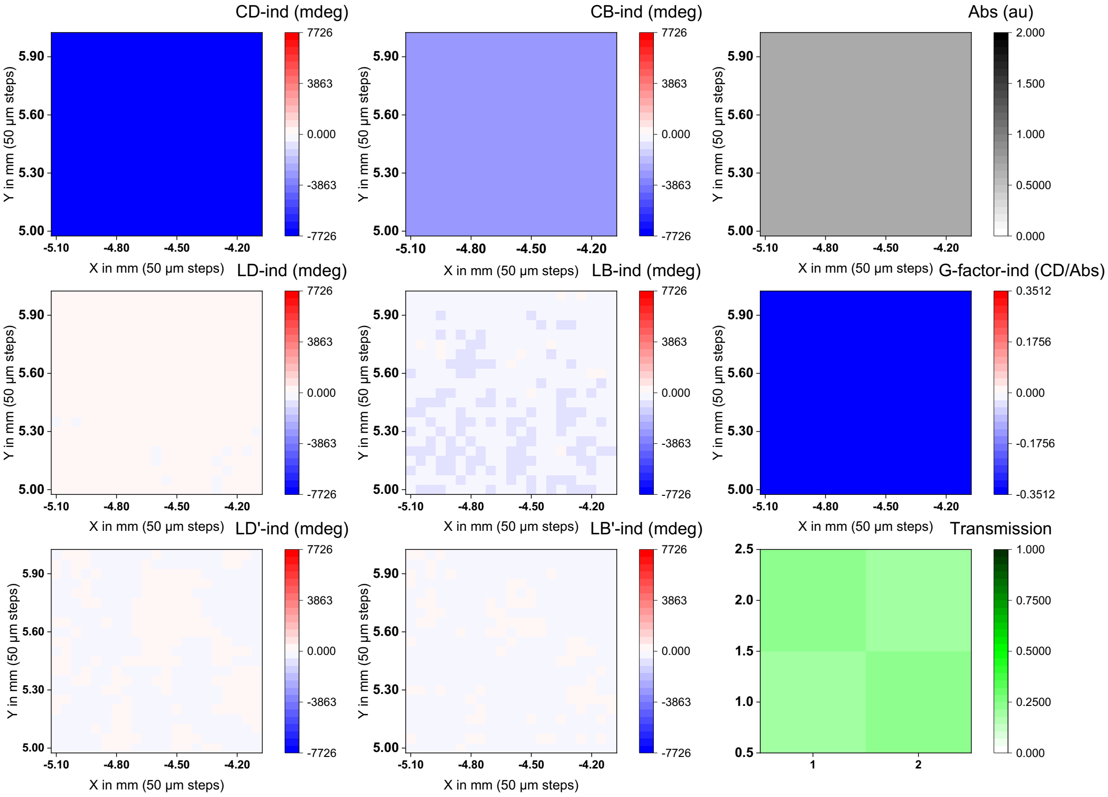

Supplementary Figure 59. CD, CB, absorption, LD, LB, g-factor, LD’, LB’ and transmission maps at 494 nm (1x1 mm,
50 μm spatial resolution) of F8BT thin film with 10% (*M,M*)-8.

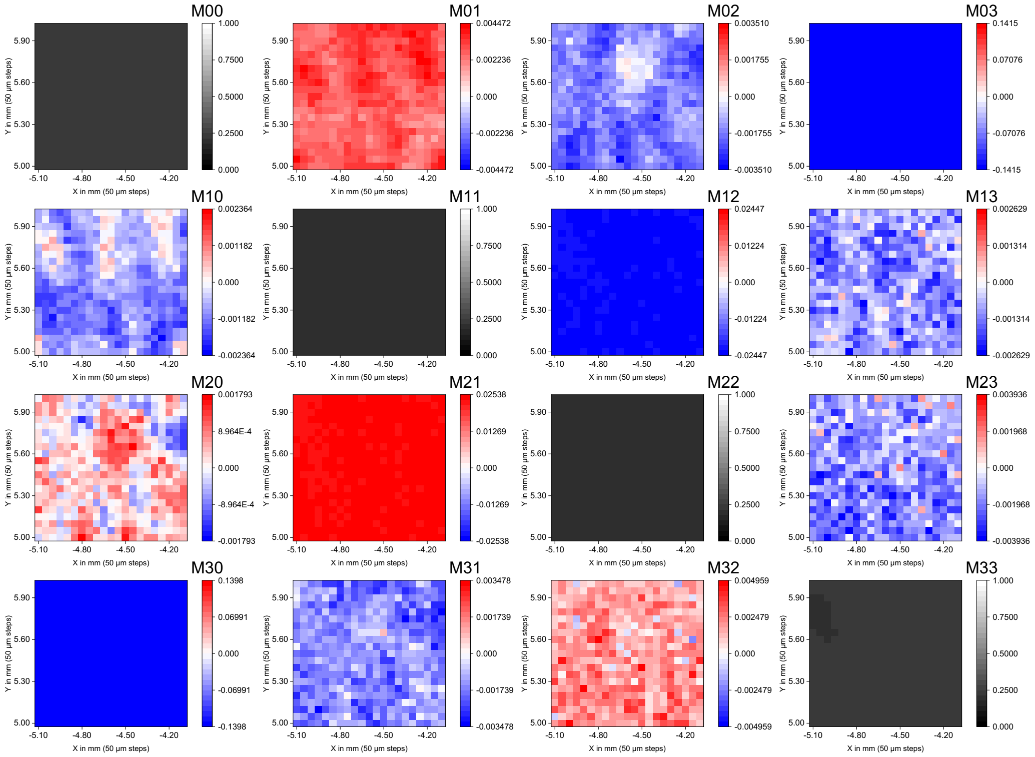

Supplementary Figure 60. Raw MMP elements maps at 494 nm (1x1 mm, 50 μm spatial resolution) of F8BT thin film with 10% (*M,M*)-8.

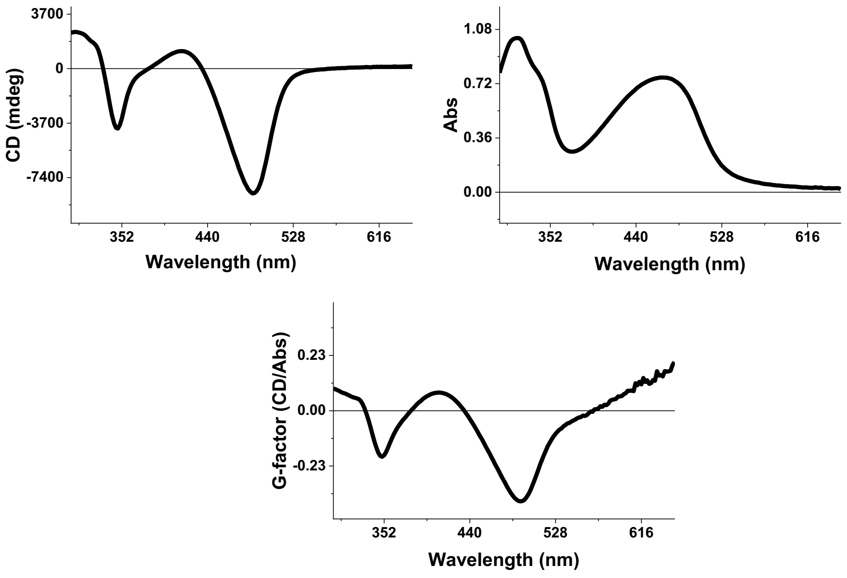

Supplementary Figure 61. CD, absorption and g-factor spectra of F8BT thin film with 10% (*M*,*M*)-8 recorded using the CD-mode of the MMP instrument.

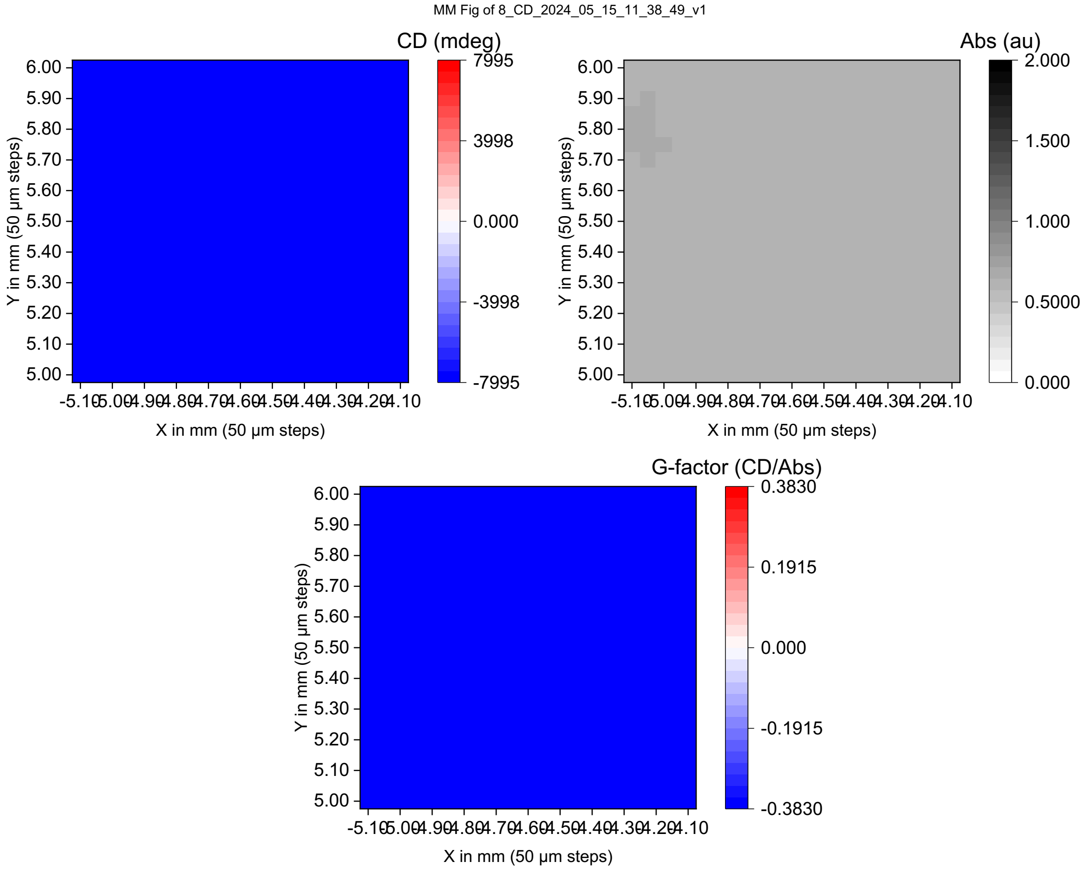

Supplementary Figure 62. CD, absorption and g-factor maps at 494 nm of F8BT thin film with 10% (*M*,*M*)-8 recorded using the CD-mode of the MMP instrument.

8. Supplementary CPL spectra

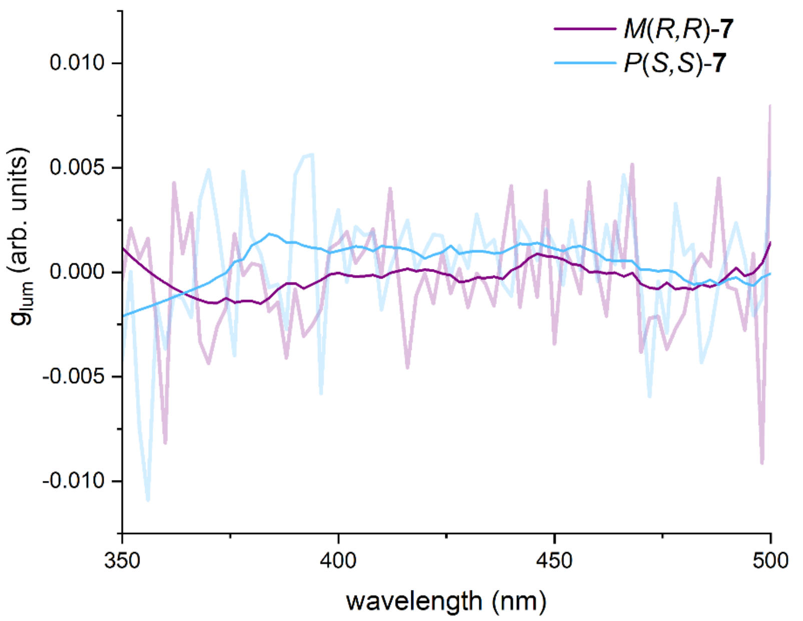

Supplementary Figure 63. CPL spectra of *M*(*R,R*)-7 (purple) and *P*(*S,S*)-*7* (light blue), $CHCl_3$ solutions. Faded lines represent the raw data and non-transparent lines represent smoothed data. The data was acquired on Chirascan CPL. The quantum yield of 7 could not be determined as it is below the detection limit of the QuantaPhi-2 integrating sphere.

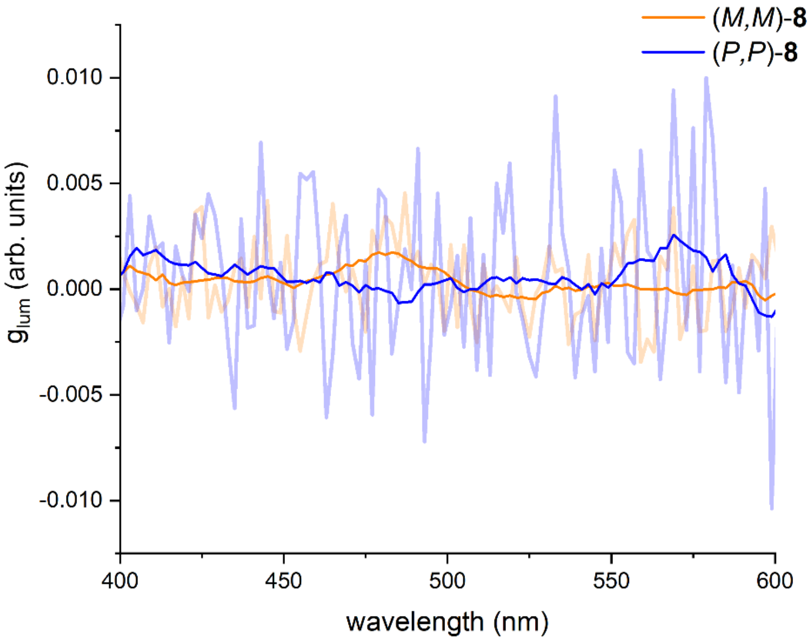

Supplementary Figure 64. g_{lum} spectra of (*M,M*)-8 (orange) and (*P,P*)-*8* (blue), $CHCl_3$ solutions. Faded lines represent the raw data and non-transparent lines represent smoothed data. The data was acquired on Chirascan CPL. The quantum yield of 8 could not be determined as it is below the detection limit of the QuantaPhi-2 integrating sphere.

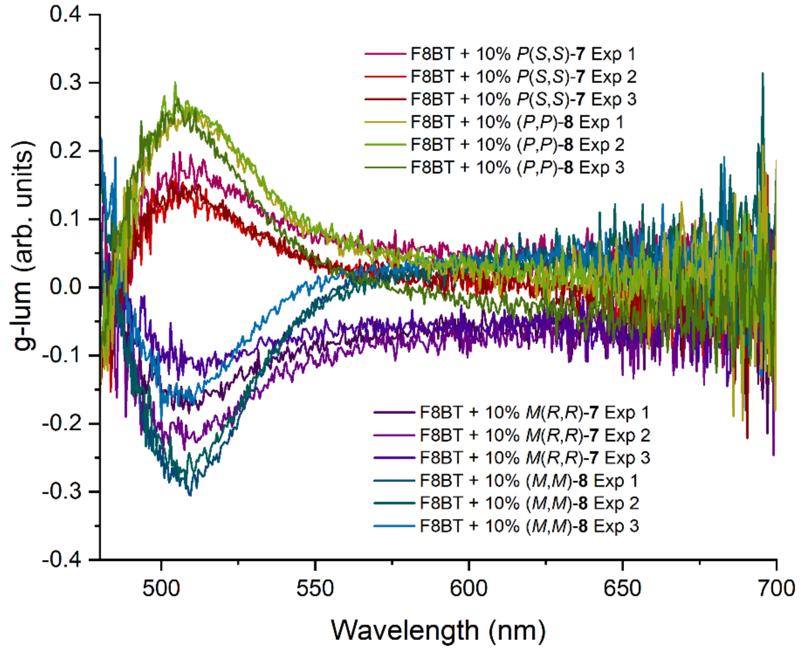

Supplementary Figure 65. g_{lum} spectra of F8BT films containing either 10% *P*(*S,S*)-7, *M*(*R,R*)-7, (*P,P*)-8 or (*M,M*)-8 as chiral dopant. Data has been recorded in triplicate.

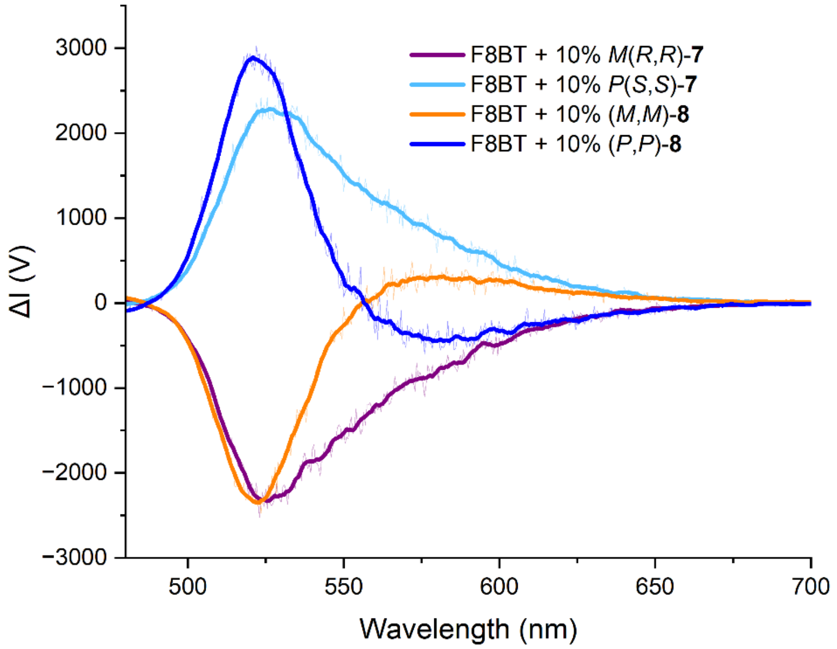

Supplementary Figure 66. delta-I spectra of F8BT films containing either 10% *P*(*S,S*)-7 (light blue), *M*(*R,R*)-7 (purple), (*P,P*)-8 (blue) or (*M,M*)-8 (orange) as chiral dopant. Faded lines represent the raw data and non-transparent lines represent smoothed data.

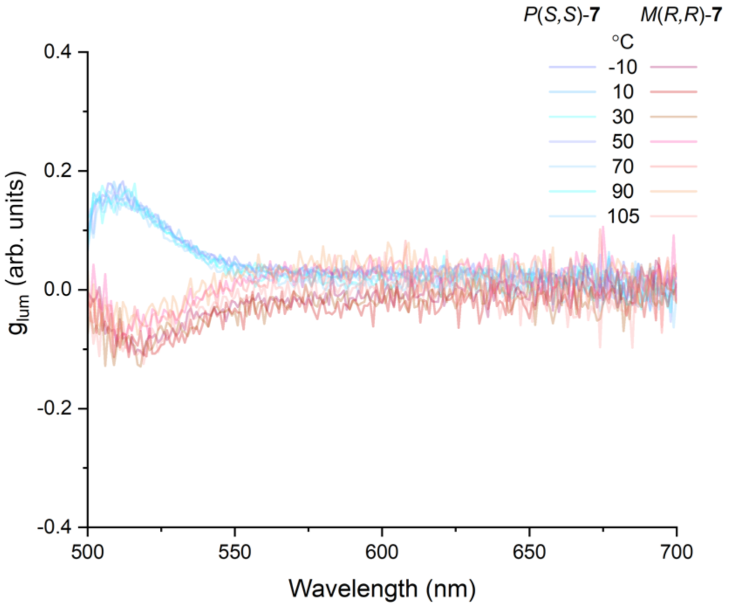

Supplementary Figure 67. Variable temperature CPL for F8BT films containing 10% *P*(*S,S*)- or *M*(*R,R*)-7 as chiral dopant, excitation @ 460 nm. The difference in g_{lum} between the films produced with enantiomeric dopants is due to the film inhomogeneity. The data was acquired on Chirascan CPL and normalized to the Olis CPL data.

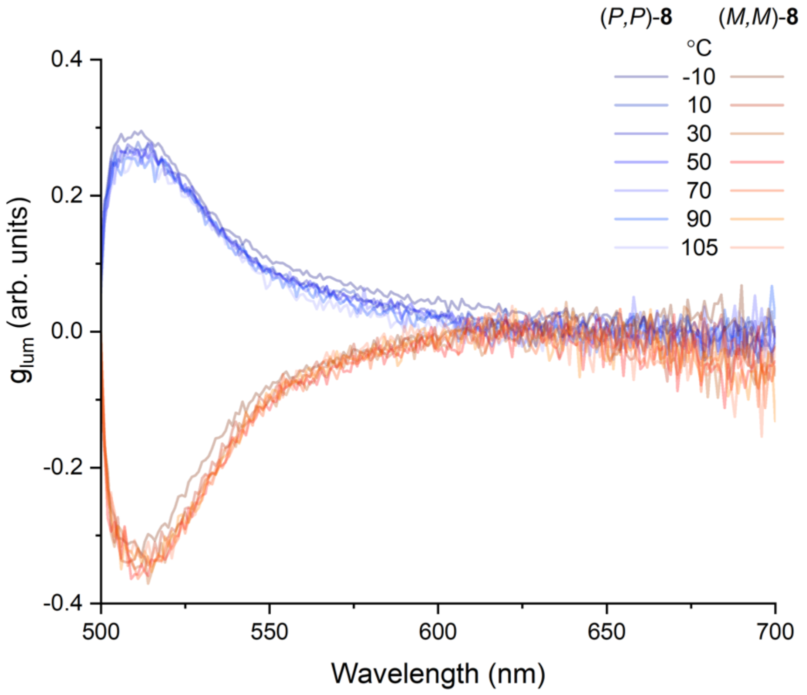

Supplementary Figure 68. Variable temperature CPL for F8BT films containing 10% (*P,P*)- or (*M,M*)-8 as chiral dopant, excitation @ 460 nm. The difference in the g_{lum} between the films produced with enantiomeric dopants is due to the film inhomogeneity. The data was acquired on Chirascan CPL and normalized to the Olis CPL data.

9. Supplementary CD and Variable Temperature CD spectra

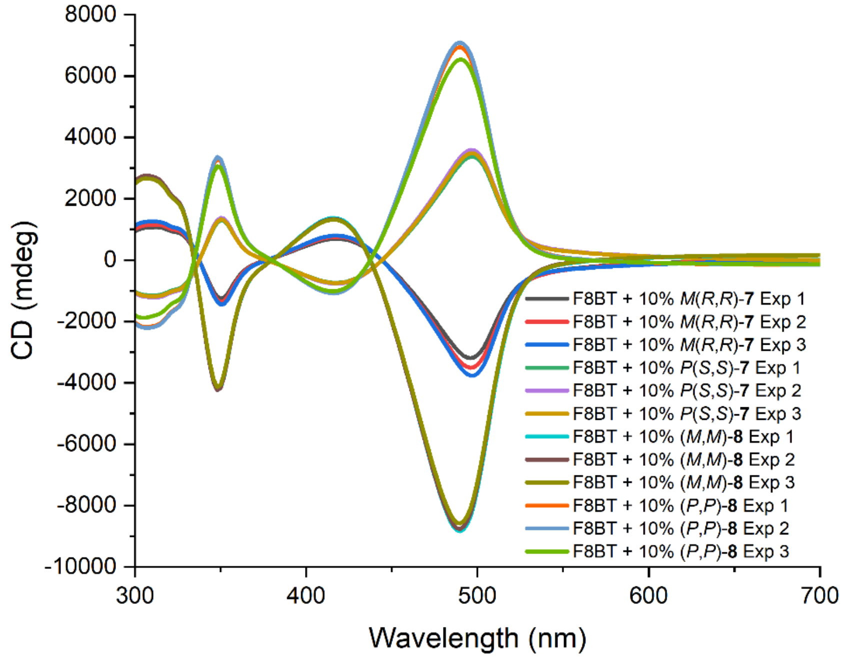

Supplementary Figure 69. CD for F8BT films containing either 10% *P*(*S,S*)-7, *M*(*R,R*)-7, (*M,M*)-8 or (*P,P*)-8 as chiral dopant. Data has been recorded in triplicate. The difference in the CD value between the films produced with enantiomeric dopants is due to the film inhomogeneity. The data was acquired on Chirascan.

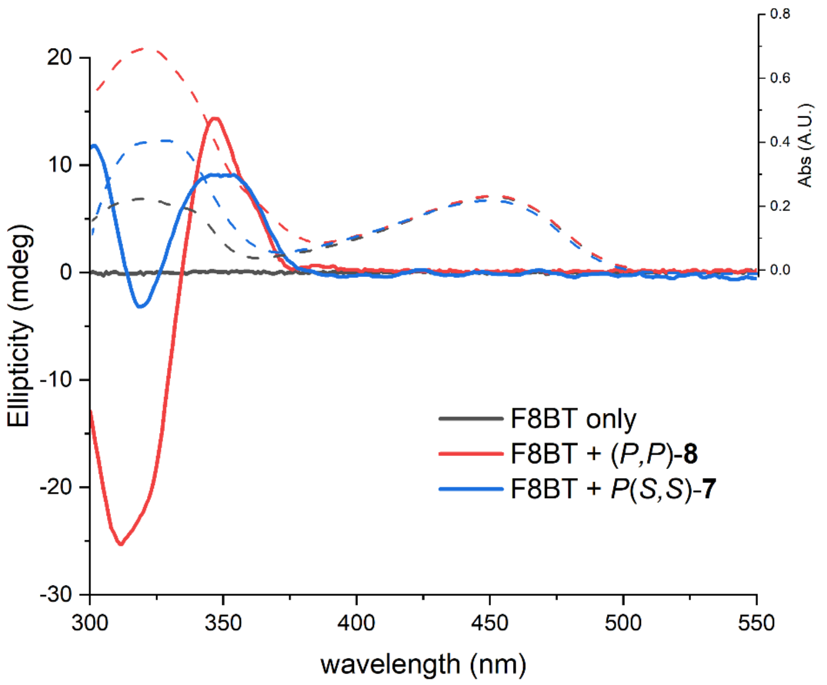

Supplementary Figure 70. CD (full lines) and Absorption (dotted lines) solution data; toluene, 20 °C. The data was acquired on Chirascan.

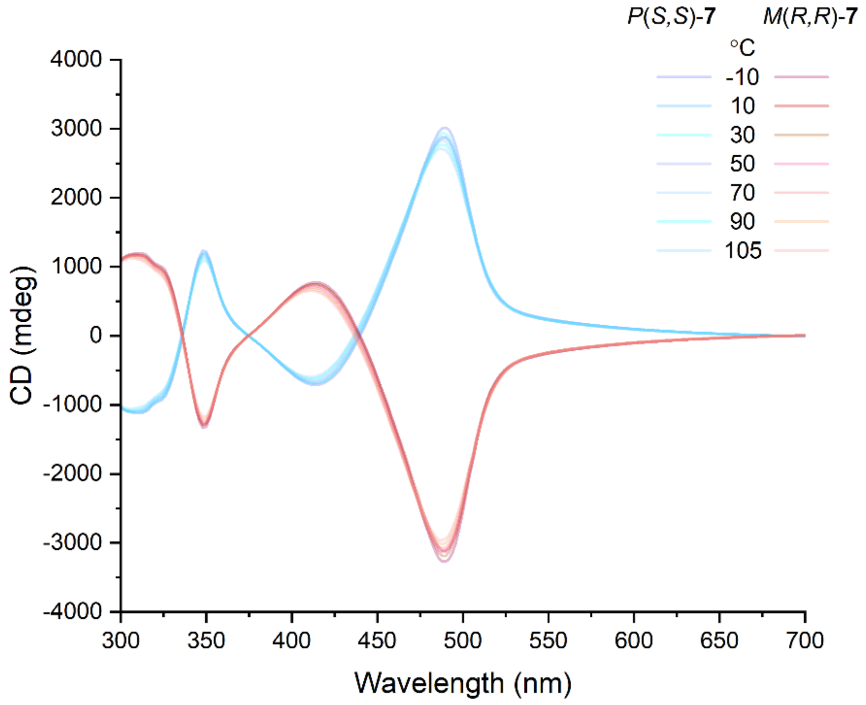

Supplementary Figure 71. Variable temperature CD for F8BT films containing 10% *P*(*S,S*)- or *M*(*R,R*)-7 as chiral dopant. The difference in the CD value between the films produced with enantiomeric dopants is due to the film inhomogeneity. The data was acquired on Chirascan.

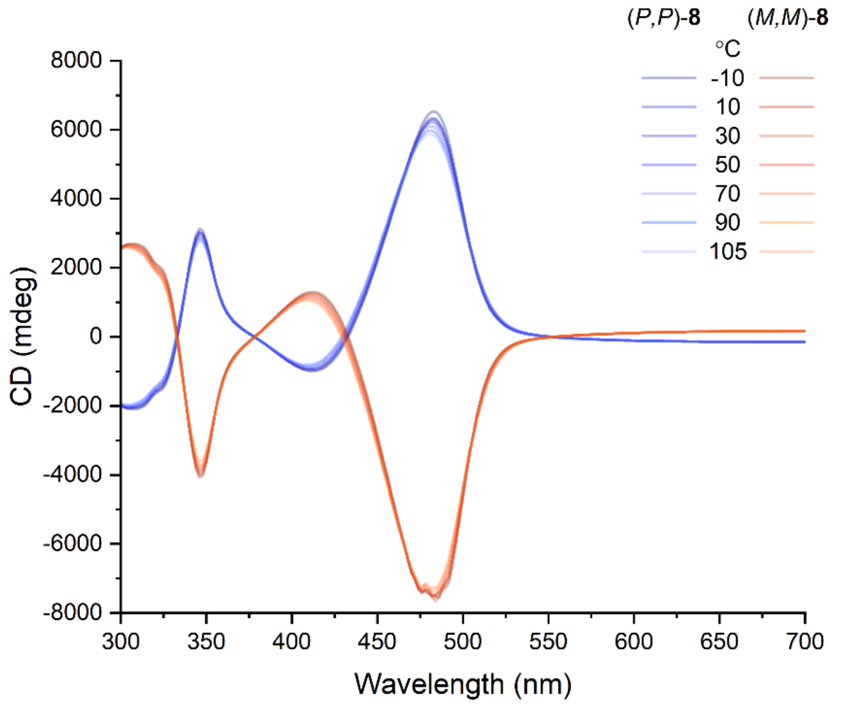

Supplementary Figure 72. Variable temperature CD for F8BT films containing 10% (*P,P*)- or (*M,M*)-8 as chiral dopant. The difference in the CD value between the films produced with enantiomeric dopants is due to the film inhomogeneity. The data was acquired on Chirascan.

10. Supplementary Emission spectra

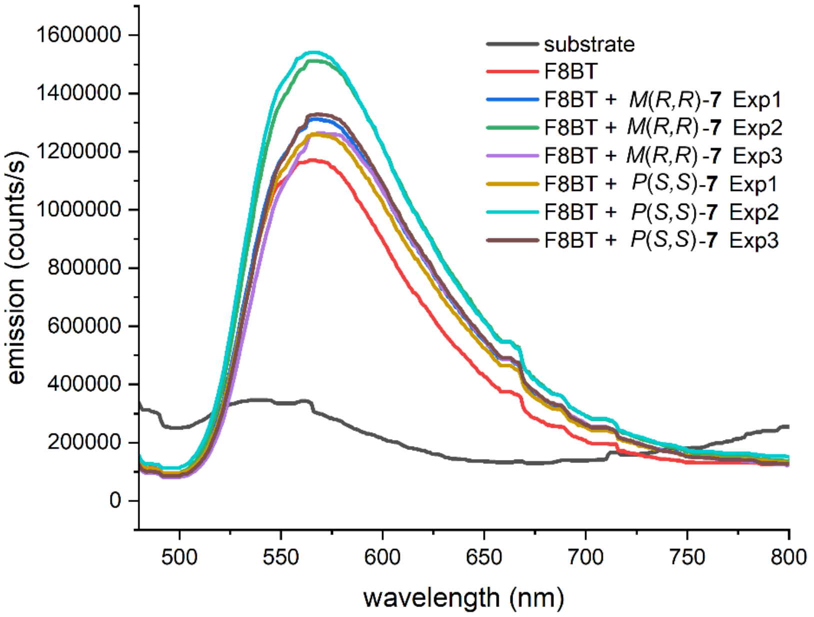

Supplementary Figure 73. Emission spectra for F8BT films containing 10% *P*(*S,S*)- or *M*(*R,R*)-7 as chiral dopant (λ_{ex} = 460 nm). Data has been smoothed to eliminate fused-silica substrate dependent scattering peaks.

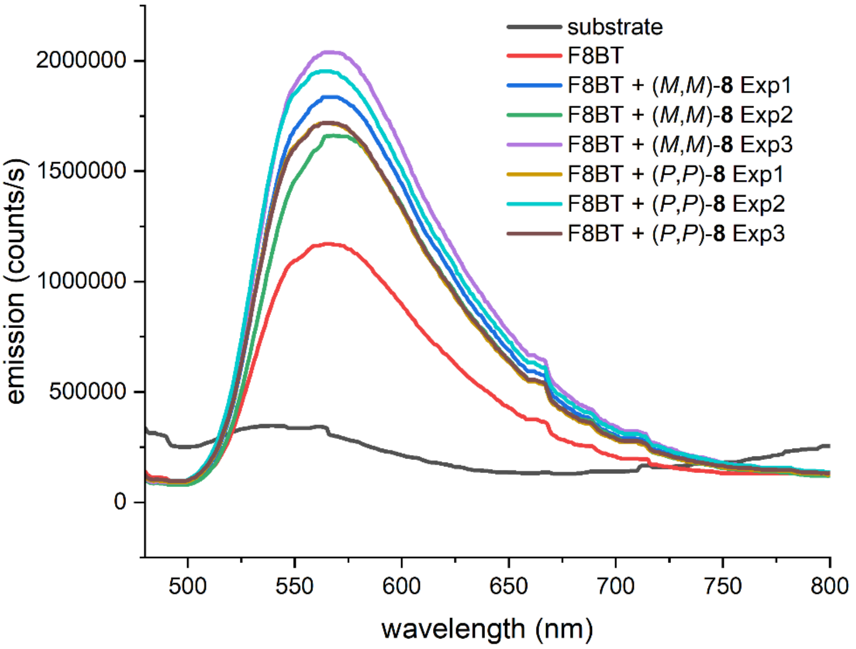

Supplementary Figure 74. Emission spectra for F8BT films containing 10% (*M,M*)- or (*P,P*)-8 as chiral dopant (λ_{ex} = 460 nm). Data has been smoothed to eliminate fused-silica substrate dependent scattering peaks.

Supplementary Table 3. Quantum yield measurements for F8BT films containing either 10% *P*(*S,S*)-7, *M*(*R,R*)-7, (*M,M*)-8 or (*P,P*)-8 as chiral dopant. Quantum yields are reported as the average of 12 measurements (2 times per point for each film, three films for each enantiomer of the dopant).

	QY (3 nm) 1s OD 3 filter	QY (3 nm) 0.1s OD 3 filter	Average
**F8BT**	0.089	0.089	0.09
**F8BT + *P*(*S,S*)-7**	0.105	0.104	0.11
	0.128	0.127	
	0.093	0.092	
**F8BT + *M*(*R,R*)-7**	0.101	0.101	0.12
	0.147	0.146	
	0.102	0.102	
**F8BT + (*M,M*)-8**	-	0.149	0.15
	0.173	0.172	
	0.127	0.126	
**F8BT + (*P,P*)-8**	0.144	0.143	0.16
	0.176	0.176	
	0.148	0.148	

11. References

[1] Crittall, M. R., Fairhurst, N. W. G. & Carbery, D. R. Point-to-helical chirality transfer for a scalable and resolution-free synthesis of a helicenoidal DMAP organocatalyst. *Chem. Commun.* **48,** 11181–11183 (2012).

[2] Neese, F. Software update: The ORCA program system—Version 5.0. *WIREs Comput. Mol. Sci.* **12,** e1606 (2022).

[3] Frisch, M. J., *et al.* Gaussian 16, Revision A.01 (Gaussian, Inc., Wallingford CT, 2016).

[4] Grimme, S., Ehrlich, S. & Goerigk, L. Effect of the damping function in dispersion corrected density functional theory. *J. Comput. Chem.* **32,** 1456–1465 (2011).

[5] Weigend, F. & Ahlrichs, R. Balanced basis sets of split valence, triple zeta valence and quadruple zeta valence quality for H to Rn: Design and assessment of accuracy. *Phys. Chem. Chem. Phys.* **7,** 3297–3305 (2005).

[6] Weigend, F. Accurate Coulomb-fitting basis sets for H to Rn. *Phys. Chem. Chem. Phys.* **8,** 1057–1065 (2006).

[7] Marenich, A. V., Cramer, C. J. & Truhlar, D. G. Universal Solvation Model Based on Solute Electron Density and on a Continuum Model of the Solvent Defined by the Bulk Dielectric Constant and Atomic Surface Tensions. *J. Phys. Chem. B* **113,** 6378–6396 (2009).

[8] Cossi, M., Rega, N., Scalmani, G. & Barone, V. Energies, structures, and electronic properties of molecules in solution with the C-PCM solvation model. *J. Comp. Chem.* **24,** 669–681 (2003).

[9] Bannwarth, C. & Grimme, S. A simplified time-dependent density functional theory approach for electronic ultraviolet and circular dichroism spectra of very large molecules. *Comp. Theor. Chem.* **1040–1041,** 45–53 (2014).

[10] White, L. E. M., *et al.* Imperial College Research Data Repository (2018–2024), DOI: 10.14469/hpc/3901.

[11] Bruhn, T., Schaumlöffel, A., Hemberger, Y., Pecitelli, G. SpecDis version 1.71, (Berlin, Germany, 2017), https:/specdis-software.jimdo.com.

[12] Nakakuki, Y., Hirose T. & Matsuda, K. Synthesis of a Helical Analogue of Kekulene: A Flexible π-Expanded Helicene with Large Helical Diameter Acting as a Soft Molecular Spring. *J. Am. Chem. Soc.* **140,** 15461–15469 (2018).
